# Accelerated degradation of cFLIP_L_ and sensitization of the TRAIL DISC-mediated apoptotic cascade by pinoresinol, a lignan isolated from *Rubia philippinensis*

**DOI:** 10.1038/s41598-019-49909-0

**Published:** 2019-09-18

**Authors:** So-Ra Lee, Khong Trong Quan, Hee Sun Byun, InWha Park, Kidong Kang, Xuezhe Piao, Eunjin Ju, Hyunju Ro, MinKyun Na, Gang Min Hur

**Affiliations:** 1 Department of Pharmacology and Department of Medical Science, College of Medicine, Chungnam National University, 266 Munhwa-ro, Daejeon, 35015 Republic of Korea; 2 College of Pharmacy, Chungnam National University, Daejeon, 34134 Republic of Korea; 3 Department of Biological Sciences, College of Biosciences and Biotechnology, Chungnam National University, Daejeon, 34134 Republic of Korea

**Keywords:** Cancer therapeutic resistance, Pharmacodynamics, Apoptosis

## Abstract

Plant-derived lignans have numerous biological effects including anti-tumor and anti-inflammatory activities. Screening of purified constituents of *Rubia philippinensis* from human glioblastoma cells resistant to TNF-related apoptosis-inducing ligand (TRAIL) has suggested that the lignan pinoresinol was a highly active TRAIL sensitizer. Here we show that treatment with nontoxic doses of pinoresinol in combination with TRAIL induced rapid apoptosis and caspase activation in many types of glioblastoma cells, but not in normal astrocytes. Analyses of apoptotic signaling events revealed that pinoresinol enhanced the formation of TRAIL-mediated death-inducing signaling complex (DISC) and complete processing of procaspase-8 within the DISC in glioblastoma cells, in which caspase-8 was inactivated. Mechanistically, pinoresinol downregulated the expression of cellular FLICE-inhibitory protein (cFLIP_L_) and survivin through proteasome-mediated degradation, without affecting death receptors or downstream intracellular apoptosis-related proteins. Furthermore, the sensitization of TRAIL-mediated apoptosis by pinoresinol strictly depended on the expression level of cFLIP_L_, which was regulated through *de novo* protein synthesis, rather than by NF-κB or p53 signaling. Taken together, our results indicate that pinoresinol facilitates DISC-mediated caspase-8 activation by targeting cFLIP_L_ in an early event in apoptotic signaling, which provides a potential therapeutic module for TRAIL-based chemotherapy.

## Introduction

The use of TNF-related apoptosis-inducing ligand (TRAIL) in cancer therapy has long been thought as an attractive strategy because it can selectively target cancer cells without affecting the majority of normal human cells^1^. The anti-cancer activity of TRAIL is attributable to its ability to elicit apoptosis through binding of its functional receptors, death receptors 4 and 5 (DR4 and DR5), and subsequent association with an adaptor protein, Fas-associated death domain (FADD)^2^. During apoptosis, FADD recruits the initiator caspases (procaspase-8 and/or procaspase-10) for the assembly of a death-inducing signaling complex (DISC). Within the DISC, the oligomerization and cleavage of the initiator caspases are the critical upstream events for activation of either the executioner caspase-3 cascade or mitochondrial-dependent apoptotic pathway via Bid cleavage, leading to sufficient apoptosis upon TRAIL treatment, depending on the specific cell type^3,4^. Importantly, genetic lesions in the components of TRAIL signaling have been found in human malignant cancers, suggesting that TRAIL functions in immune surveillance against developing cancers and metastasis^5–7^. Indeed, mice null of TRAIL receptor were shown to susceptible to inflammation and tumorigenesis with apoptotic defects^8^. Consistent with this possibility, currently evaluated TRAIL agonistic antibodies have demonstrated a significant therapeutic efficacy in a number of preclinical studies^9,10^. However, therapeutic benefits in clinical trials have been rather limited due to the appearance of cancer cells that escape the cytotoxicity induced by TRAIL-targeted therapy^11,12^. Thus, the discovery of a therapeutic strategy module that can eradicate cancer cells without restoring resistance has been pending in the field of TRAIL-based chemotherapy.

The genus *Rubia* (family Rubiaceae), a perennial herb, is widely distributed worldwide. It is one of the most attractive plant resources because of its potent and wide spectrum of *in vivo* and *in vitro* biological activities, which include anti-cancer, anti-inflammatory, and anti-angiogenic effects^13–15^. In a recent phytochemical study of *Rubia philippinensis Elmer*, we isolated several compounds, including derivatives of anthraquinones, pentacyclic triterpenoids, cyclopeptides, and lignans^16–18^. Although several studies have reported the anti-cancer effects of *Rubia* species, the effects of the principle constituents of *R*. *philippinensis* on DR-mediated cell death, particularly during TRAIL sensitization, have not yet been determined. As part of our ongoing search to identify potential therapeutic approaches for sensitizing TRAIL-mediated cell death, we tested 33 compounds isolated from *R*. *philippinensis* and found that nontoxic doses of pinoresinol, a lignan, drastically sensitized cancer cells against TRAIL-induced apoptosis. Pinoresinol facilitated DISC formation to trigger a caspase-8-dependent apoptotic cascade activation in TRAIL-resistant glioblastoma cells. Moreover, our findings revealed novel evidence that the prominent sensitizing effects of pinoresinol against TRAIL-mediated apoptosis involved the downregulation of levels of cellular FLICE-inhibitory protein (cFLIP_L_) by a mechanism involving *de novo* protein synthesis.

## Results

### IIdentification of pinoresinol from *R*. *philippinensis* as a TRAIL sensitizer in TRAIL resistant glioma cells

**Figure 1:**
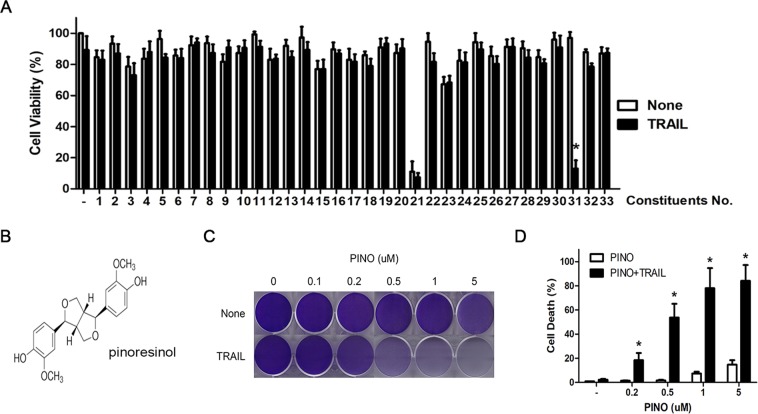
Identification of pinoresinol as a potent TRAIL sensitizer from the constituents of *R. philippinensis*. (**A**) Screening of the principle constituents of *R. philippinensis* for potential cytotoxic enhancer in TRAIL resistant glioblastoma cells. LN428 cells were pretreated with a series of constituents (5 μM) for 30 min, followed by 50 ng/ml TRAIL for 24 h. Cell death was quantified by using Cell Titer-glo Luminescent cell viability assay kit as described as Methods. (**B**) Chemical structure of pinoresinol (PINO). (**C**,**D**) LN428 cells were pretreated with indicated concentrations of PINO, followed by 50 ng/ml TRAIL. After 24 h, cells were fixed, stained and photographed. (**C**) Cell death was quantified as in A. (**D**) Data were normalized to the rate of spontaneous cell death occurring in untreated cells. Data represents the mean ± SE of three independent experiments. Statistical difference (*p < 0.05) compared with the PINO only-treated group are indicated.

We characterized a set of major compounds obtained from *R*. *philippinenesis* to identify active constituents that synergistically sensitized the cytotoxic effects of TRAIL in TRAIL-resistant glioblastoma cells (Supplementary Table S1, Supplementary Figs 1–33). Treatment of LN428 cells with 50–200 ng/mL TRAIL alone induced a limited number of cell deaths (<5%) over 24 h (data not shown). In the screening assay, LN428 cells were sequentially treated with the purified compounds and 50 ng/mL TRAIL, followed by an ATP-based cell viability assay. In parallel, we tested the cytotoxicty of each compound on LN428 cells as single agents. Of the compounds screened, the lignin pinoresinol was a potent sensitizer of TRAIL-mediated cytotoxicity (Fig. 1A,B). It eliminated the survival of LN428 cells but only in the presence of TRAIL; it had only marginal growth inhibitory effects as a single agent (Fig. 1C). Consistently, no cell death was observed when cells were treated with pinoresinol alone at concentrations up to 1 μM over 24 h. By contrast, combined treatment with the same concentrations of pinoresinol and TRAIL resulted in a drastic increase in cell death (Fig. 1D), thus confirming that this combination resulted in extensive cell death at low concentrations (0.2–1 μM) of pinoresinol.

### Sensitization of TRAIL-induced killing by pinoresinol is associated with a caspase-8-dependent apoptotic cascade in glioma cells

**Figure 2:**
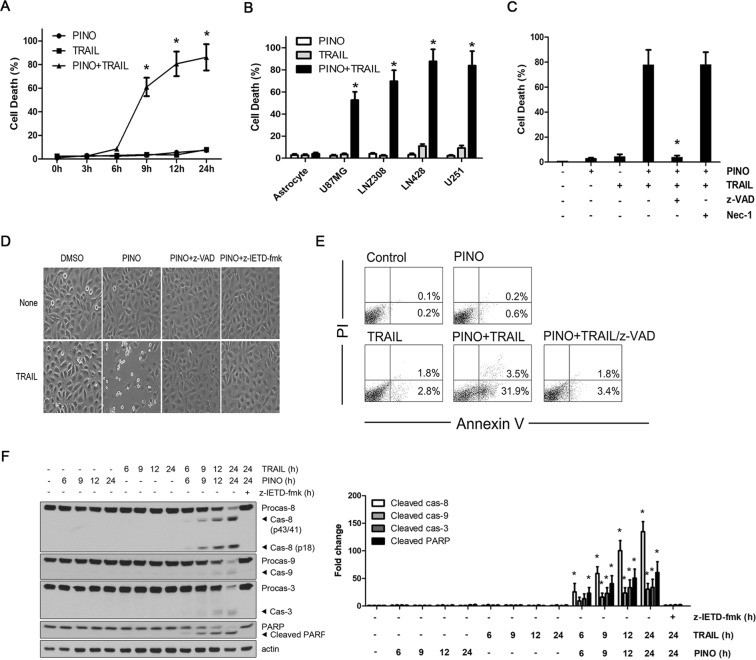
Sensitization of TRAIL-induced apoptosis by non-toxic dose of pinoresinol requires caspase-8 activation in various glioblastoma cells, but not in normal astrocytes. (**A**) LN428 cells were treated with PINO (0.5 μM), TRAIL (50 ng/ml) and TRAIL (50 ng/ml) plus PINO (0.5 μM) for the indicated times. (**B**) TRAIL resistant glioblastoma cells (U87MG, LNZ308, LN428, and U251) and normal primary astrocytes were treated with PINO, TRAIL and TRAIL plus PINO for 24 h. Cell death was quantified as in Fig. 1A. Data were normalized to the rate of spontaneous cell death occurring in untreated cells. Data represents the mean ± SE of three independent experiments. *p < 0.05, compared with the TRAIL only-treated group. (**C**,**D**) LN428 cells were treated with PINO, TRAIL and TRAIL plus PINO for 24 h in the absence or presence of caspase or necroptosis inhibitor z-VAD-fmk (20 μM)/z-IETD-fmk (50 μM) or Nec-1 (30 μM). (C) Cell death was quantified as in A. Data represents the mean ± SE of three independent experiments. *p < 0.05, compared with the PINO/TRAIL-treated group. (**D**) Cells were visualized using an inverted microscope. (**E**) LN428 cells were treated with PINO, TRAIL and TRAIL plus PINO for 24 h in the absence or presence of z-VAD-fmk. Cells were subjected to Annexin V/PI staining, and then analyzed by flow cytometry. (**F**) LN428 cells were treated with PINO (0.5 μM), TRAIL (50 ng/ml) and TRAIL plus PINO for indicated times. Whole cell lysates were subjected to immunoblotting with the indicated antibodies (left) and densitometry analysis of the bands from the relevant proteins was performed (right). Data represents the mean ± SE of three independent experiments. *p < 0.05, compared with the TRAIL only-treated group.

Next, to validate the above screening results, we performed kinetic experiments to evaluate the synergistic induction of cell death using a nontoxic concentration (0.5 μM) of pinoresinol. TRAIL-mediated cytotoxicity began to appear from 9 h after pinoresinol co-treatment, and rapidly increased up to 24 h (Fig. 2A). In addition, pinoresinol had a similar synergistic efficacy against TRAIL-mediated cytotoxicity in three other glioblastoma cell lines (U87MG, LNZ308, and U251). By contrast, normal astrocytes were not sensitive to TRAIL-induced killing when treated with pinoresinol (Fig. 2B). These results suggest that the TRAIL-sensitizing effects of pinoresinol might apply to a wide spectrum of glioblastoma cell lines that could be limited to cancer cells. Although TRAIL-induced cancer cell death was mainly apoptotic, it might also induce non-apoptotic cell death via non-canonical TRAIL signaling depending on the cellular context^19–21^. Thus, next, we examined whether cell death caused by pinoresinol plus TRAIL was associated with caspase-dependent apoptosis. As expected, pretreatment of LN428 cells with pancaspase and the irreversible caspase 8-inhibitors z-VAD-FMK and z-IETD-fmk completely abrogated the cytotoxicity induced by pinoresinol plus TRAIL (Fig. 2C,D). However, necrostain-1, an inhibitor of programmed necrosis, failed to protect against cell death, indicating that pinoresinol predominantly triggered apoptotic, rather than necrotic, cell death. To confirm the mode of TRAIL-mediated cell death sensitized by pinoresinol, cell death was analyzed by Annexin V and propidium iodide (PI) staining followed by flow cytometry. Consistently, treatment of pinoresinol plus TRAIL drastically increased the population of an early phase of apoptosis (Annexin V^+^), whereas very few cells were stained exclusively with PI, and such increased apoptotic population was prevented by co-treatment with z-VAD-FMK (Fig. 2E). To get more insights into the mechanisms underlying TRAIL-sensitized apoptosis, we sequentially analyzed the activation of processes of caspase signaling cascade, including those of initiator caspase (caspase-8) and as executor caspases (caspase-3 or −9) and the resultant PARP cleavage. In the kinetic analysis, we found that the treatment of pinoresinol plus TRAIL caused an activation of caspase-8 and −3, and PARP cleavage from 6 h onwards (Fig. 2F). Furthermore, pretreatment with z-IETD-fmk completely inhibited the activation of caspase cascade induced by pinoresinol plus TRAIL treatment. These results clearly indicate that casapse-8 activation is essential in the sensitization of pinoresinol-induced apoptosis in TRAIL-resistant glioblastoma cells.

### Sensitizing efficacy of pinoresinol on TRAIL-mediated apoptosis is not associated with either NF-κB or p53

**Figure 3:**
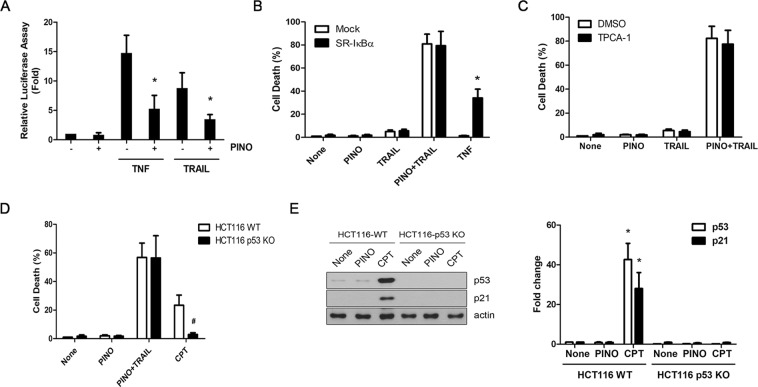
Sensitizing effect of pinoresinol against TRAIL-mediated apoptosis is not associated with either NF-κB or p53 signaling pathway. (**A**) LN428 cells were transfected with an NF-κB-responsive reporter plasmid (p2xNF-B-Luc) and pRSV-β-gal. After 24 h, cells were treated with PINO (0.5 μM) alone for 6 h, or pretreated with PINO (0.5 μM) for 30 min, followed by TNF (30 ng/ml) or TRAIL (50 ng/ml) for additional 6 h. The luciferase assays were performed as describe in Methods, and the activity of each sample was normalized according to β-galactosidase activity. Each column shows the mean ± SE of three independent experiments. *p < 0.05, compared with the TNF or TRAIL only-treated group. (**B**) LN428 cells were transfected with mock or IκBα super-repressor (SR-IκBα) plasmid. After 24 h, cells were treated with PINO, TNF, TRAIL or TRAIL plus PINO for additional 24 h. (**C**) LN428 cells were pretreated with IKKα/β inhibitor TPCA-1 (0.5 μM) for 30 min, followed by PINO, TRAIL or TRAIL plus PINO for additional 24 h. (**D**) Wild-type and p53 null HCT116 cells were treated with PINO, TRAIL plus PINO and campthothecin (Cpt, 100 μM) for 24 h. (**B**–**D**) Cell death was quantified as in Fig. 1A. Data were normalized to the rate of spontaneous cell death occurring in untreated cells. Data represents the mean ± SE of three independent experiments. *p < 0.05, compared with the mock-transfected group. ^#^*p* < 0.05, compared with the wild-type HCT116 cells. (**E**) Wild-type and p53 null HCT116 cells were treated with PINO and campthothecin for 24 h. Whole cell lysates were subjected to immunoblotting with the indicated antibodies (left) and densitometry analysis of the bands from the relevant proteins was performed (right). *p < 0.05, compared with the none-treated group.

Pinoresinol exhibits anti-inflammatory properties via blockade of the NF-κB pathway in several immune and cancer cells^22–24^. Given the well-established ability of NF-κB to regulate TRAIL resistance through induction of its anti-apoptotic genes^25^, it was hypothesized that the anti-NF-κB effects of pinoresinol might contribute to sensitization against TRAIL-induced apoptosis. Consistent with previous studies^22–24^, pretreatment of LN428 cells with pinoresinol significantly decreased the transcriptional activity of NF-κB induced by either TNF or TRAIL, while pinoresinol alone did not affect the basal level of NF-κB activity (Fig. 3A). However, unexpectedly, LN428 cells with prevention of NF-κB activation by overexpression of the IκBα super-repressor (SR-IκBα), which could not be phosphorylated due to substitutions of serine 32 and serine 36 by alanine, were found to have a similar extent of cell death after TRAIL or pinoresinol plus TRAIL treatment, although TNF-induced cell death was drastically enhanced (Fig. 3B). In addition, pretreatment with the NF-κB inhibitor TPCA failed to affect cell death upon TRAIL or pinoresinol plus TRAIL treatment (Fig. 3C). These results suggest that the TRAIL-sensitizing efficacy conferred by pinoresinol was unlikely to be a result of NF-κB inhibition. Although p53 activation plays an important role in TRAIL sensitization^1,26^, the involvement of p53 in pinoresinol-induced TRAIL sensitization was excluded because LN428 cells retained mutant-p53^27^. Consistent with this possibility, we found that pinoresinol treatment also sensitized TRAIL-induced cell death in p53 null HCT116 cells to a similar extent as it did with wild type (WT) cells, despite the remarkable differences in cytotoxicity after camptothecin treatment between these two cell types (Fig. 3D). Moreover, no detectable induction of p53 and its target p21 upon pinoresinol alone or pinoresinol plus TRAIL treatment in WT-HCT116 cells, despite the fact that the substantial amounts of p53 and p21 were induced by a DNA damaging agent, camptothecin in WT-HCT116 cells but not in p53- null HCT116 cells (Fig. 3E). Such findings thus indicate that pinoresinol’s effect on TRAIL-mediated cell death is independent of p53.

### Pinoresinol accelerates DISC formation by down-regulating cFLIP_L_ expression

**Figure 4:**
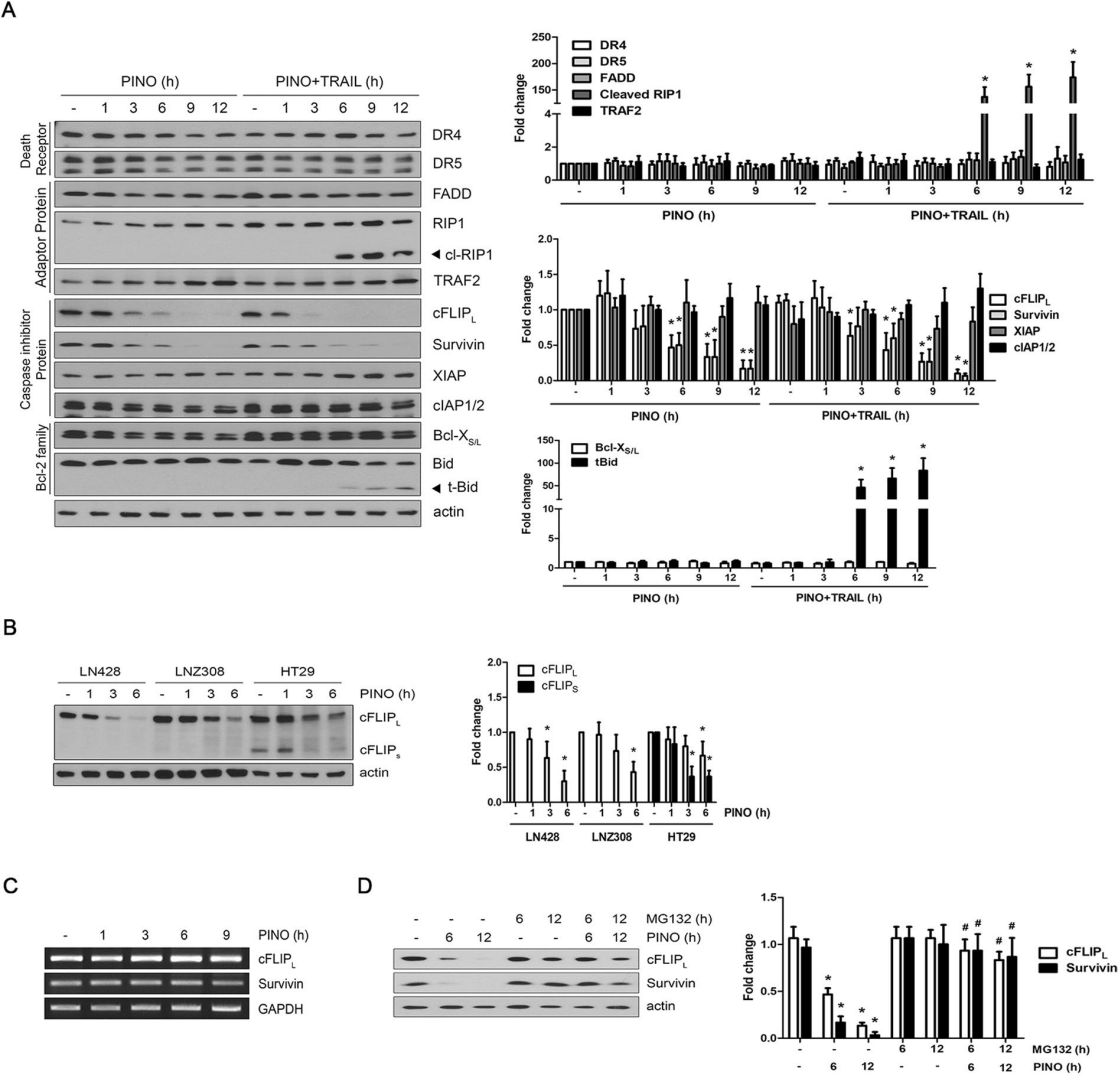
Pinoresinol down-regulates the expression of cFLIP_L_ and survivin at the post-translational levels via proteasome-mediated degradation. (**A**) LN428 cells were treated with PINO (0.5 μM) in the absence or presence of TRAIL (50 ng/ml) for the indicated times. (**B**) LN428, LNZ308 and HT29 cells were treated with PINO (0.5 μM) for the indicated times. Whole cell extracts were subjected to immunoblotting with the indicated antibodies (left). Densitometry analysis of the bands from the relevant proteins was performed (right). Data represents the mean ± SE of three independent experiments. *p < 0.05, compared with none-treated group. (**C**) Total RNA was prepared at the indicated times after PINO (0.5 μM) treatment in LN428 cells, and RT-PCR was performed with the primers specific to human cFLIP_L_, survivin and GAPDH. After PCR amplification, the products were separated by agarose gel electrophoresis and visualized using ethidium bromide staining. (**D**) LN428 cells were treated with PINO (0.5 μM) for the indicated times in the absence or presence of proteasome inhibitor MG-132 (10 μM). Whole cell extracts were subjected to immunoblotting with the indicated antibodies (left), and densitometry analysis of the bands from the relevant proteins was performed (right). Data represents the mean ± SE of three independent experiments. *p < 0.05, compared with none-treated group. ^#^*p* < 0.05, compared with PINO only-treated group.

To characterize the underlying mechanism involved in pinoresinol-induced sensitization of glioma cells against TRAIL-mediated apoptosis, we determined the expression levels of several apoptosis-related proteins in the death receptor signaling pathway after exposure to pinoresinol for different times in LN428 cells. Notably, the protein expression levels of the long isoform of cellular FLIP (cFLIP_L_) and survivin were drastically decreased in a time-dependent manner in LN428 cells with pinoresinol (Fig. 4A, panels 6,7). Reductions of cFLIP_L_ and survivin were accompanied by increased levels of cleaved-RIP1 and truncated-Bid (t-Bid) in cells upon pinoresinol plus TRAIL treatment (Fig. 4A, panels 4,11). Furthermore, we observed that pinoresinol was also able to down-regulate cFLIP_S_ expression with a similar kinetics with cFLIP_L_ in HT-29 cells, though the expression level of cFLIP_S_ was extremely low or nondetectable in glioblastoma cells including LN428 cells and LNZ308 cells (Fig. 4B). These results suggest that expression of cFLIP isoforms is highly cell type-specific and pinoresinol-induced cFLIP downregulation, especially in cFLIP_L_, may play a predominant role in sensitizing TRAIL-mediated apoptosis in glioblastoma cells. By contrast, the protein levels of signaling components of TRAIL including death receptors, adaptor proteins, other inhibitor of apoptosis proteins, and Bcl-2 family proteins were not affected or only modestly affected in cells after pinoresinol treatment. The expression of cFLIP_L_ and survivin proteins are regulated by either transcriptional or post-translational modifications such as ubiquitin-mediated proteasomal degradation^28–31^. In contrast to the observed down-regulation of cFLIP_L_ and survivin protein levels, treatment with pinoresinol did not change their mRNA levels at any of the time points examined (Fig. 4C). However, pretreatment with the proteasome inhibitor MG132 sufficiently prevented the down-regulation of cFLIP_L_ and survivin expression by pinoresinol (Fig. 4D), suggesting that pinoresinol might reduce the protein levels of cFLIP_L_ and survivin via proteasome-mediated degradation rather than through transcriptional control.

**Figure 5:**
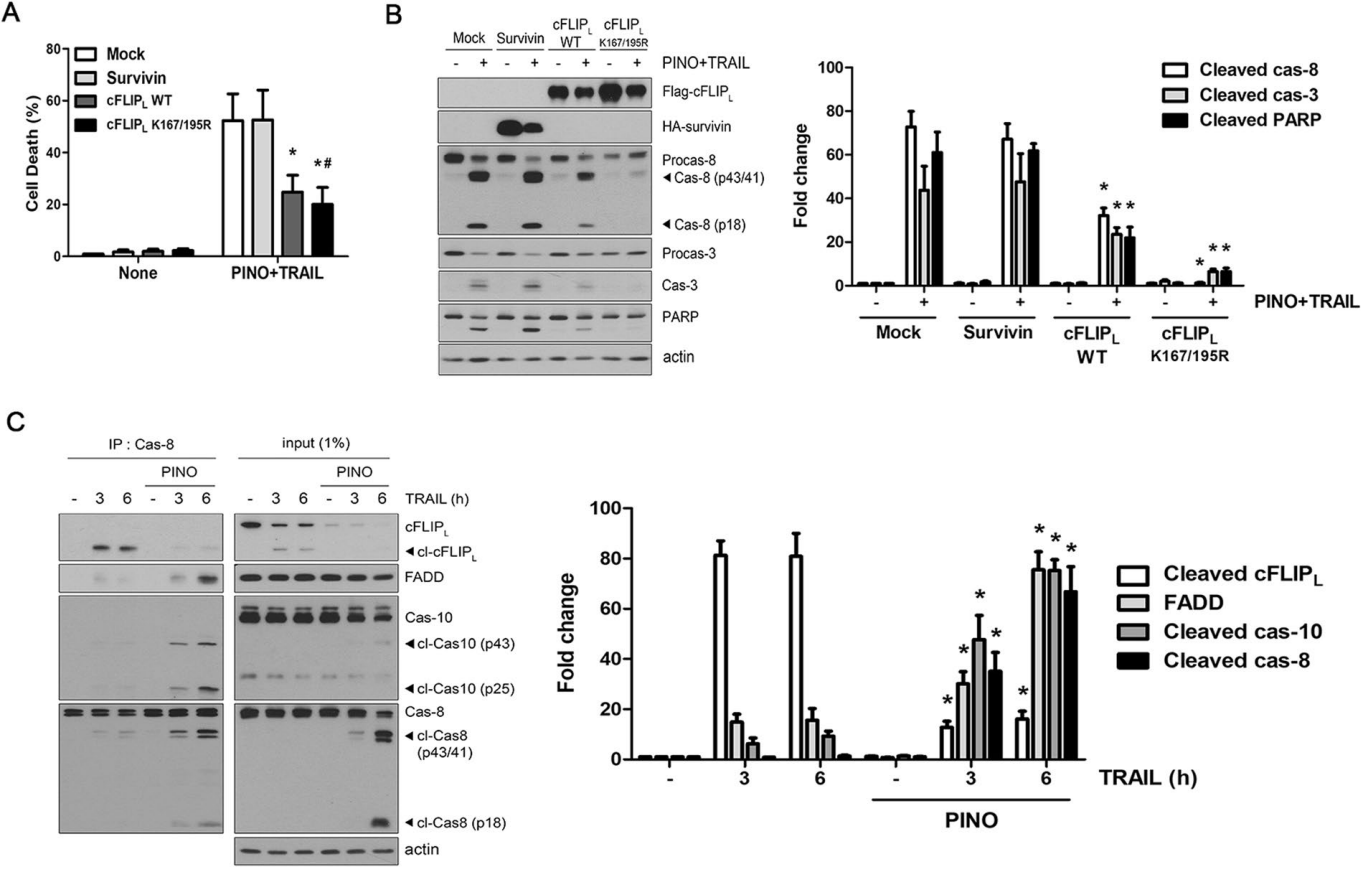
Pinoresinol-mediated down-regulation of cFLIP_L_ accelerates TRAIL DISC formation. (**A**,**B**) LN428 cells were transfected with plasmids expressing flag-tagged FLIP_L_ (wild type; WT or K167/195 R) and WT-survivn plasmids for 24 h, and followed by TRAIL (50 ng/ml) plus PINO (1 μM) for 12 h. (**A**) Cell death was quantified as in Fig. 1A. Data were normalized to the rate of spontaneous cell death occurring in untreated cells. Data represents the mean ± SE of three independent experiments. *p < 0.05, compared with the mock-transfected group. ^#^*p* < 0.05, compared with the wild-type FLIP_L_-transfected group. (**B**) Whole cell extracts were subjected to immunoblotting with the indicated antibodies (left), and densitometry analysis of the bands from the relevant proteins was performed (right). Data represents the mean ± SE of three independent experiments. *p < 0.05, compared with mock-transfected group. (**C**) LN428 cells were treated with TRAIL (50 ng/ml) in the absence or presence of PINO (0.5 μM) for the indicated times. Cell extracts from each sample were subjected to immunoprecipitation with anti-caspase-8 antibody. Immunoprecipitants were analyzed by immunoblotting with indicated antibodies. A total of 1% of the cell extract volume from each sample was used as input control (left). Densitometry analysis of the bands from the relevant proteins was performed (right). Data represents the mean ± SE of three independent experiments. *p < 0.05, compared with none-treated group.

Next, we examined whether downregulation of cFLIP_L_ and survivin by pinoresinol affected the facilitated TRAIL-mediated cytotoxicity by overexpressing these genes in LN428 cells. Consistent with its critical function to antagonize caspase-8, overexpression of WT cFLIP_L_ resulted in a significant decrease in cell death and caspase cascade activation induced by pinoresinol plus TRAIL treatment (Fig. 5A,B). We also found that a cFLIP_L_ mutant (cFLIP_L_-K167/195R containing modified major ubiquitin acceptor sites) more profoundly abrogated pinoresinol plus TRAIL-induced caspase-dependent apoptosis, compared to that of WT cFLIP_L_. However, the cell death induced by pinoresinol plus TRAIL was not affected by the overexpression of survivin, suggesting that downregulation of upstream anti-apoptotic protein anti-cFLIP_L_, rather than survivin, contributed to an important mechanism involving pinoresinol sensitization of TRAIL-induced cell death.

The cFLIP isoforms compete directly with procaspase-8 for binding to FADD in a TRAIL-dependent fashion, thus inhibiting procaspase-8/-10 recruitment to form the DISC^31,32^. It is therefore possible that downregulation of cFLIP_L_ by pinoresinol might directly affect the formation of the DISC, an early signaling event in TRAIL-induced apoptosis. An immunoprecipitation assay using an anti-caspase-8 antibody revealed that treatment of LN428 cells with TRAIL in the absence of pinoresinol led to efficient recruitment of cleaved cFLIP_L_ to the isolated DISC, whereas DISC-bound FADD and caspase-8/-10 were only weakly detected (Fig. 5C, left panel, rows 1–3). These results suggest that in LN428 cells, the TRAIL-induced recruitment of cFLIP_L_ into the DISC was an important step before caspase-8 activation to exhibit resistance against TRAIL cytotoxicity. However, pretreatment with pinoresinol promoted an increase in TRAIL-mediated DISC formation and procaspase-8/10 processing, concomitant with decreasing amounts of DISC-bound cFLIP_L_ (Fig. 5C, left panel, rows 4–6). More importantly, in pinoresinol-pretreated cells, activation of procaspase-8 processing within the DISC following TRAIL treatment proceeded to completion, as shown by the appearance of the active p18 subunit of mature caspase-8. Taken together, these results strongly suggest that a reduced amount of DISC-bound cFLIP_L_ played a major role in TRAIL sensitization by pinoresinol.

### Pinoresinol-mediated down-regulation of cFLIP_L_ is mediated via *de novo* protein synthesis inhibition

**Figure 6:**
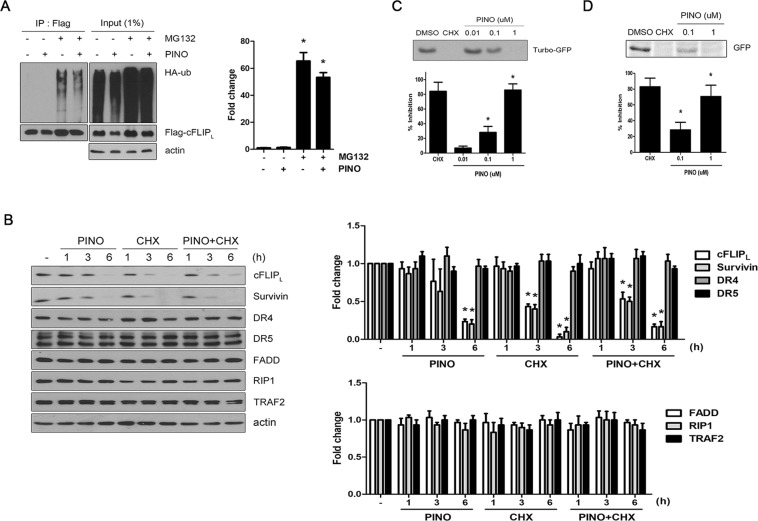
Pinoresinol-induced down-regulation of cFLIP_L_ is mediated via *de novo* protein synthesis inhibition. (**A**) LN428 cells were co-transfected with plasmids expressing flag-tagged cFLIP_L_ and HA-tagged ubiquitin plasmids for 24 h. The cells were then treated with PINO (0.5 μM) for 4 h in the absence or presence of MG-132 (10 μM). Cell extracts from each sample were subjected to immunoprecipitation with anti-flag antibody followed by immunoblotting with anti-HA antibody for detection of ubiquitinated cFLIP_L_. A total of 1% of the cell extract volume from each sample was used as input control (left). Densitometry analysis of the bands from the relevant proteins was performed (right). Data represents the mean ± SE of three independent experiments. *p < 0.05, compared with none-treated group. (**B**) LN428 cells were treated with PINO (0.5 μM), cycloheximide (CHX, 10 μM) and PINO (0.5 μM) plus CHX (10 μM) for indicated times. Whole cell extracts were subjected to immunoblotting with the indicated antibodies (left). Densitometry analysis of the bands from the relevant proteins was performed (right). Data represents the mean ± SE of three independent experiments. *p < 0.05, compared with none-treated group. (**C**) The *in vitro* coupled transcription and translation assay was performed using 1-Step Human Coupled IVT Kit as described in Methods. pCFE-GFP plasmid as template in HeLa cell lysate supplemented with indicated concentrations of CHX and PINO. (**D**) *In vitro* translation inhibition assay with increased amount of PINO. *In vitro* transcribed mRNA encoding *EGFP* was purified as described in Methods. 3 µg of EGFP mRNA each in HeLa cell lysate was incubated for 6 h with indicated concentrations of CHX and PINO. The *in vitro* translation yield of EGFP protein was monitored by immunoblotting with anti-Turbo GFP or anti-GFP antibody, respectively and quantified using a densitometry from the relevant proteins (top). Percent inhibition of protein synthesis was calculated by dividing the band intensities from each concentration of PINO over control DMSO-treated samples (bottom).

Next, we identified the underlying mechanism by which pinoresinol directly controls ubiquitin-mediated degradation of cFLIP_L._ As expected, co-immunoprecipitation analyses showed that treatment of cells with MG132 led to an increase in polyubiquitinated cFLIP_L_ with concomitant enhanced protein levels (Fig. 6A). However, we unexpectedly detected lower levels of ubiquitinated cFLIP_L_ in cells treated with pinoresinol plus MG132, compared to cells exposed to MG132 cells, indicating that the accelerated proteasomal degradation of cFLIP_L_ by pinoresinol was not achieved through direct activation of the ubiquitination process. Given that cFLIP_L_ and survivin are unstable proteins with a rapid turnover^29,33^, we addressed whether the reduced protein levels by pinoresinol were associated with *de novo* protein synthesis of cFLIP_L_ and survivin. Treatment with either pinoresinol or cycloheximide (CHX) did not influence the cellular amounts of DRs and adaptor proteins, including DR4/5, FADD, RIP1, and TRAF2 (Fig. 6B). By contrast, pinoresinol was able to down-regulate the expression levels of cFLIP_L_ and survivin with similar kinetics to that of CHX. Furthermore, the down-regulating effect by either pinoresinol or CHX was not accelerated by the combined treatment of pinoresinol and CHX. These results suggest that in a similar manner to CHX, pinoresinol inhibited *de novo* synthesis of proteins with a rapid turnover cFLIP and survivin.

To directly assess whether the down-regulation of protein expression by pinoresinol is due to the impairment of the general translational machinery, we conducted a cell-free *in vitro* transcription and translation assay. As shown in Fig. 6C, pinoresinol suppressed the production of green fluorescent protein (GFP), similar to a well-known protein translation inhibitor CHX, in a dosage dependent manner. To rule out the possibility that pinoresinol-dependent suppression of protein synthesis is caused by hampering transcriptional processes, subsequent *in vitro* translation response was assessed by using *in vitro* synthesized EGFP mRNA. Consistently, incubation with 1 μM pinoresinol completely interfered the EGFP protein production with a similar efficacy of 10 μM CHX (Fig. 6D). Taken together, these data indicate that pinoresinol directly interferes a *de novo* protein synthesis without affecting transcriptional machinery.

## Discussion

Glioblastoma is a heterogenous group of invasive malignant primary brain tumors with high mortality^34^. Although all populations of cancer cells contribute in their own way to drive tumor growth, the molecular changes disrupting the apoptotic pathway are considered a pathological hallmark of glioblastoma. Emerging evidence suggests that cells within the glioblastoma exhibit abnormalities of the cell death pathway such as overexpression of antiapoptotic proteins or silencing of key death effectors^35,36^. Importantly, genomic analyses of human glioblastomas have shown that caspase-8, an essential component of the DISC, is frequently inactivated by either gene mutations or promotor methylation^7,37–39^. Thus, resistance to DR-mediated cytotoxicty in glioblastoma cells might occur as a step of DISC assembly. Consistent with this possibility, we found that in a series of glioblastoma cells including LN428 cells treated with TRAIL, complete activation of caspase-8 and functional DISC formation were blocked. However, pinoresinol treatment resulted in cells resistant to apoptosis with an increased recruitment of both procaspase-8 and FADD to the TRAIL DISC, and complete activation of caspase-8. It is therefore possible that pinoresinol-induced TRAIL sensitization was conducted at the level of the DISC/caspase-8 axis.

Accumulating evidence presently suggests that cFLIP is a key player in the DR-mediated apoptotic pathway that retains the sublethal activation of caspase-8 at the DISC^31,32,40^. Consequently, elevated levels of cFLIP in tumor tissues from patients with a variety of cancers including lung cancer, Burkitt’s lymphoma, cervical caricinoma and colorectal carcinoma are correlated with poor clinical outcomes^41–44^, implicating the existence of a strong association between suppression of DISC-mediated apoptosis by cFLIP and tumorigenesis. An important finding from the present study is that protein levels of cFLIP_L_ were significantly reduced by pinoresinol treatment, and the ectopic overexpression of cFLIP_L_, significantly suppressed caspase-8 activation and reduced the susceptibility to cell death caused by pinoresinol/TRAIL treatment in LN428 cells. Changes in the expression levels of cFLIP_L_ by pinoresinol therefore appear to be responsible for TRAIL sensitization in glioma cells. In this regard, it is important to determine the potential mechanism involved in the pinoresinol-induced downregulation of cFLIP_L_ expression. It has previously been reported that cFLIP_L_ expression is tightly regulated at the transcriptional level by a number of stimuli, including NF-κB transcription factor^45,46^, mitogen-activated protein kinase^47^ and protein kinase B/Akt^48,49^. Previous pharmacological and biochemical studies have reported that pinoresinol exhibits anti-inflammatory and anti-cancer effects, in part through the inhibition of NF-κB^22–24^. It is therefore possible that pinoresinol suppresses cFLIP_L_ expression through NF-κB inhibition. In accordance with previous observations, we found that pinoresinol potently inhibited the NF-κB activity in LN428 cells in response to TNF and TRAIL. However, we did not observe a decrease in cFLIP_L_ mRNA expression levels in cells treated with pinoresinol concentrations of 0.5–1 μM, which caused cFLIP_L_ depletion and maximal TRAIL sensitization. Furthermore, the expressions of a subset of NF-κB-inducible genes, including *TRAF2*, *cIAP1/2*, *XIAP*, and *Bcl-X*_*L*_, were unaffected by pinoresinol treatment. These findings raise the possibility that down-regulatory effects of pinoresinol on cFLIP_L_ expression are not associated with transcriptional regulation of NF-κB. These discrepancies of transcriptional regulation between cFLIP_L_ expression and NF-κB may have resulted from different concentrations of pinoresinol. Indeed, pinoresinol must be used at a relatively high concentration (≥10 μM) to exhibit anti-NF-κB activity in several types of cells^22,23,50^, suggesting that other factors may be involved in the effects of cFLIP_L_ depletion by low concentrations of pinoresinol.

On the other hand, the expression levels of cFLIP_L_ were regulated by the ubiquitin–proteasomal pathway with a short half-life^29,51^. In this respect, it is of particular interest that, in LN428 cells, pinoresinol induces proteasomal degradation of cFLIP_L_ or ectopic expression of the cFLIP_L_ mutant (cFLIP_L_-K167/195 R), which significantly abolishes sensitization by pinoresinol to TRAIL-induced cytotoxicity. Furthermore, the results of an *in vitro* translational assay showed that pinoresinol directly inhibited *de novo* protein synthesis, which has similar efficiency with the well-known protein synthesis inhibitor CHX. These results raise the possibility that pinoresinol disrupts *de novo* protein synthesis, particularly for fast turnover proteins such as cFLIP_L_, leading to proteasomal degradation with decreased stability. Nevertheless, it is currently unclear how pinoresinol inhibits *de novo* protein synthesis. Further studies to identify the ribosomal proteins that interact with pinoresinol in the translational machinery will be critical for a complete understanding of the mechanism of action, and for the development of novel TRAIL-based chemotherapies. Earlier, it has been reported that survivin plays an essential role in cell cycle progression^52^. In this study, we found that pinoresinol induced a G2/M arrest with an increase in the G2 population (Supplementary Fig. 34), suggesting that down regulation of survivin by pinoresinol might be relevant in limiting cell division, especially in G2-M phase rather than apoptosis. In addition, it has recently been reported that pinoresinol suppresses the efflux of chemotherapeutic drugs outside by interacting with P-glycoprotein (P-gp) encoded by multidrug resistant-1 (MDR-1) gene^53,54^. Since P-gp efflux function was shown to contribute to TRAIL resistance via controlling the endogenous level of TRAIL in certain types of cancer cells^55,56^, such an anti-Pgp activity of pinoresinol might constitute another mechanism in enhancing TRAIL efficacy in TRAIL-resistant cancers including glioblastoma. Thus, future bioavailability study of TRAIL and pinoresinol using *in vivo* preclinical models are required to further validate TRAIL and pinoresinol-based therapeutic development for glioblastoma.

## Methods

### Isolation of pinoresinol

Pinoresinol was isolated from our chemical study on *Rubia philippinensis*, as described previously^17^. Briefly, a methylene chloride (CH_2_Cl_2_)-soluble fraction (50 g) was prepared from solvent extraction of *R. philippinensis* extract (150 g) using CH_2_Cl_2_ and water. Vacuum liquid chromatography of CH_2_Cl_2_-soluble fraction on silica gel column (20 × 20 cm) eluting with *n-*hexanes-EtOAc (20:1, 10:1, 5:1, 3:1, 2:1) and CHCl_3_-MeOH (8:1) resulted in the preparation of six column fractions (D-1 → D-6). Fraction D-6 (10 g) was separated by reversed-phase C18 column chromatography [column: SNAP cartridge KP-C18-HS (400 g), mobile phase: MeOH-H_2_O (10:90 → 100:0, 7 L)] and 11 subfractions (D-6-1 → D-6-11) were obtained. Pinoresinol (t_*R*_ 34.0 min, 40 mg) was purified from D-6-4 (360 mg) by preparative HPLC [column: Phenomenex C_18_ (250 × 21.2 mm), mobile phase: MeOH-H_2_O (50:50, 4 mL/min)]. Pinoresinol: brownish amorphous powder; ESIMS *m/z* 359.2 [M + H]^+^, 381.1 [M + Na]^+^, 357.1 [M-H]^−^, 393.1 [M + Cl]^−^;^1^H NMR (300 MHz, methanol-*d*_4_): 3.13 (2H, m, H-8, H-8′), 3.80 (2H, Ha-9, Ha-9′), 3.85 (6H, s, OCH_3_-3, OCH_3_-3′), 4.22 (2H, Hb-9, Hb-9′), 4.70 (2H, d, *J* = 2.7, H-7, H-7′), 6.77 (2H, overlapped, H-5, H-5′), 6.79 (2H, overlapped, H-6, H-6′), 6.95 (2H, H-2, H-2′); ^13^C NMR (75 MHz, methanol-*d*_4_): 133.8 (C-1, C-1′), 111.0 (C-2, C-2′), 149.1 (C-3, C-3′), 147.3 (C-4, C-4′), 116.1 (C-5, C-5′), 120.0 (C-6, C-6′), 87.5 (C-7, C-7′), 55.3 (C-8, C-8′), 72.6 (C-9, C-9′), 56.4 (OCH_3_-3, OCH_3_-3′).

### Structure determination of pinoresinol

The molecular formula of purified compound was deduced as C_20_H_22_O_6_ based on the ESIMS protonated ion at *m/z* 359.2, the sodium-adduct ion at *m/z* 381.1, the deprotonated ion at *m/z* 357.1, and the chloride-adduct ion at *m/z* 393.1 (calcd. for C_20_H_22_O_6_, *m/z* 358.1). The ^1^H NMR data displayed a pair of symmetric benzene ring system at *δ*_H_ 6.77 (2H, overlapped, H-5, H-5′), 6.79 (2H, overlapped, H-6, H-6′), 6.95 (2H, H-2, H-2′). The proton signals at *δ*_H_ 3.13 (2H, m, H-8, H-8′), 3.80 (2H, Ha-9, Ha-9′), 4.22 (2H, Hb-9, Hb-9′), 4.70 (2 H, d, *J* = 2.7, H-7, H-7′) indicated two symmetrical tetrahydrofuran substructure of lignan. In the ^13^C NMR spectroscopic data, six benzene signals at *δ*_C_ 111.0~149.1, two oxygenated carbons at *δ*_C_ 87.5 and 72.6, one methine at *δ*_C_ 55.3, and a methoxy group at *δ*_C_ 56.4 supported the symmetric structure of tetrahydrofuran-type lignan. The chemical structure was identified as pinoresinol by NMR spectroscopic and LC-MS data analyses^57^.

### Antibodies and chemicals

The antibodies and chemicals were obtained from the following resources; anti-PARP (#556362), anti-XIAP (#610716), anti-FADD (#610399), anti-RIP1 (#610459), anti-p53 (#554147) and anti-p21 (#556430) antibodies (BD Biosciences, San Diego, CA, USA); anti-caspase-3 (#9662), anti-caspase-8 (#9746), anti-caspase-9 (#9508) and anti-Bid (#2002) antibodies (Cell signalling Technology, Beverly, MA, USA); anti-caspase-10 (M059-3) antibody (MBL, WOBURN, MA, USA); anti-Bcl-X_S/L_ (sc-271121), anti-survivin (sc-17779), anti-TRAF2 (sc-876), anti-GFP (sc-9996) and anti-HA (sc-805) antibodies (Santa Cruz, CA, USA); anti-c-FLIP (ALX-804-961) antibody (Enzo Life Sciences, Farmingdale, NY, USA); anti-cIAP1/2 (#07-759) antibody (Upstate Biotech, Waltham, MA, USA); anti-DR5 (#ab181846) antibody (Abcam, Cambridge, UK); anti-DR4 (NB100-56528) antibody (Novus, Centennial, CO, USA); anti-TurboGFP (PA5-22688) antibody (Thermo scientific, Waltham, Massachusetts, USA); anti-actin (A2066), anti-flag (F3165, 1:2,000 dilution) antibodies (Sigma-Aldrich, St. Louis, MO, USA). the pan caspase inhibitor Z-VAD-FMK, MG-132, TPCA-1 (Calbiochem, San Diego, CA, USA); recombinant TNF (R & D Systems, Minneapolis, MN, USA); recombinant human TRAIL/Apo2 ligand (Peprotech, Rocky Hill, NJ, USA).

### Cell culture and primary astrocytes preparation

Human malignant glioblastoma cells (LN428, LNZ308, U87MG, and U251MG) were kindly provided by Dr. Yongwan Kim (Dongsung Cancer Center, Daegue, Korea). HCT 116, a human colorectal cancer cells and its p53-knockout derivates were kindly provided by Dr. Deug Y. Shin (Dankook University, Cheonan, Korea). Cells were cultured in Dulbecco’s modified Eagle’s medium (DMEM) including 10% fetal bovine serum, 2 mmol/L glutamine and 100 U/mL penicillin/streptomycin. The normal primary astrocytes were prepared from the neonatal rats, as described previously^58^, and cultured in Minimum Essential Media (MEM) with 10% FBS, 2 mmol/L glutamine and 100 U/mL penicillin/ streptomycin.

### Plasmids, transfection and luciferase assay

The expression plasmids of survivn (pCA-flag-survivn) and cFLIP_L_ (pCA-flag- cFLIP_L_) were gift from Dr. Taeg K. Kwon (Keimyung University, Daegu, Korea). The point-mutant of cFLIP_L_ at lysine 167/195 (cFLIP_L_-K167/195 R) was generated by a Quickchange^TM^ Site-Directed Mutagenesis kit as previously described^59^. For luciferase assay, LN428 cells were transiently co-transfected with p2xNF-kB-Luc and pRSV-β-galactosidase into 1 × 10^6^ cells per well in 6-well plates for 24 h using Lipofectamine reagent (Invitrogen). Cell were then treated with TNF (30 ng/ml) or TRAIL (50 ng/ml) for an additional 6 h, and the luciferase activities were measured using a luciferase assay kit (Promega, Madison, CA, USA) according to the manufacturer’s instructions. Luciferase activity obtained were normalized to β-galactosidase activity of each sample.

### Cell death assessment

Cell death was determined using Cell Titer-glo Luminescent Cell Viability Assay kit (Promega Co., Fitchburg, WI, USA), according to the manufacturer’s instructions. Luminescent signals were measured by Tecan Infinite Plate reader (Tecan group Ltd., Männedorf, Switzerland), and Viability rates were calculated following the formula: viability rates = (1 − medicating/control) × 100%. Representative images were also taken by an inverted microscope.

### Flow cytometry analysis

After LN428 cells were treated with pinoresinol, as described in the figure legends, the cells were harvested and examined for the mode of apoptotic and necrotic cell death by double staining with FITC-conjugated annexin V and propidium iodide (PI) in 10 mM HEPES buffer, pH 7.4, according to the manufacturer’s instructions (BD FITC Annexin V kit). For cell cycle analysis, cells were suspended with ice-cold phosphate buffered saline (PBS) and fixed in 70% ethanol. Cells were washed with PBS and added 100 μg/ml PI solution including 50 μg/ml RNase in PBS for 30 min at room temperature. The cells were analyzed with a FACScan flow cytometer (BD Biosciences).

### Immnunoblotting and immunoprecipitation

Upon treatment, cells were lysed in ice-cold M2 buffer (20 mM Tris, pH 7.6, 0.5% NP-40, 250 mM NaCl, 3 mM EDTA, 3 mM EGTA, 2 mM dithiothreitol, 0.5 mM PMSF, 20 mM β-glycerol phosphate, 1 mM sodium vanadate, and 1 µg/ml leupeptin). For immunoblot analysis, cell lysates were resolved on 8–12% SDS-polyacrylamide gel (PAGE), transferred onto the PVDF membrane (GE Healthcare Life Sciences). Membranes were serially incubated with specific primary antibodies (1:1,000 dilution) and secondary antibody (1:2,000 dilution), and immunoblots were visualized by enhanced chemiluminescence (ECL) kit (EMD Millipore). For immunopreciptation assays, the cell lysates were incubated with anti-caspase-8 antibody and protein A-agarose beads at 4 °C overnight. The immunoprecipitants were washed three times with M2 buffer and analyzed by immunoblotting.

### RT-PCR analysis

Total RNA was isolated using a ReliaPrep RNA Miniprep kit (Promega), and cDNA was prepared using a M-MLV reverse transcriptase (Promega), according to the manufacturer’s instructions. PCR amplification was carried out using the primer pairs specific to each human genes (cFLIP_L_, 5′-CTGGTTGCCCCAGATCAACT-3′ and 5′-CCCAGGGAAGTGAAGGTGTC-3′; Survivin, 5′-TGACGACCCCATAGAGGAACA-3′ and 5′-TCAATCCATGGCAGCCAGC-3′; GAPDH, 5′-CACCATCTTCCAGGAGCGAG-3′and 5′-GATGGCATGGACTGTGGTCA-3′).

### ***In vitro*** translation analysis

*In vitro* coupled transcription and translation analysis was performed with the 1-Step Human Coupled IVT Kit ± DNA (ThermoFisher Scientific) following the manufacturer’s instructions. Briefly, the translation reaction was assembled with pre-incubation of HeLa cell lysates with 1 μg of circular DNA (pCFE-GFP) as a template. The reaction mixtures were incubated for 12 hours at 30 °C and were stopped with loading buffer for SDS-PAGE. The expression levels of EGFP proteins were examined by immunoblot analysis using anti-TurboGFP antibody. For *in vitro* translational analysis, EGFP mRNA was synthesized after digesting pcGlobin2-EGFP with XhoI, as described previously^60^. Synthetic EGFP mRNA was generated by using a mMESSAGE mMACHINE T7 Transcription kit (ThermoFisher Scientific) following manufacturer’s instructions. Each 3 µg of purified EGFP mRNA was incubated with HeLa cell lysates for 6 hours at 30 °C with indicated concentrations of PINO and CHX as described in figure legends. The expression level of EGFP was measured through immunoblotting with anti-GFP antibody and then quantified by densitometry using an image J software.

### Statistical analysis

Data are expressed as the mean ± S.E. from at least three separate experiments performed triplicate. Statistical analysis was performed using one-way analysis of variance (ANOVA), followed by the Bonferroni *t*-test for multi-group comparison tests. Student’s *t*-test was used to compare the mean values from the two groups. P < 0.05 was considered as statistically significant.

## Supplementary information

Supplementary Informarion
