# Supplementary material for: Accelerated degradation of cFLIPL and sensitization of the TRAIL DISC-mediated apoptotic cascade by pinoresinol, a lignan isolated from Rubia philippinensis
Source: Sci Rep. 2019 Sep 18;9:13505. doi: 10.1038/s41598-019-49909-0 (PMC6751165; doi:10.1038/s41598-019-49909-0)

Accelerated degradation of cFLIP$_L$ and the sensitization of TRAIL DISC-mediated apoptotic cascade by pinoresinol, a lignan isolated from *Rubia philippinensis*

So-Ra Lee[1], KhongTrong Quan[2], Hee Sun Byun[1], InWha Park[2], Kidong Kang[1], Xuezhe Piao[1], Eunjin Ju[1], Hyunju Ro[3], MinKyun Na[2*], Gang Min Hur[1*]

[1]*Department of Pharmacology and Department of Medical Science, College of Medicine, Chungnam National University, 266 Munhwa-ro, Daejeon 35015, Republic of Korea*

[2]*College of Pharmacy, Chungnam National University, Daejeon 34134, Republic of Korea*

[3]*Department of Biological Sciences, College of Biosciences and Biotechnology, Chungnam National University, Daejeon 34134, Republic of Korea*

Supplementary Table S1. Phytochemicals 1-33 derived from *R. philippinensis*

No	Name of the compound	Mass m/z (molecular formula)
1	2-methyl-1,3,6-trihydroxy-9,10-anhthraquinone 3-*O*-(6'-*O*-acetyl)-*α*-rhamnosyl (1→2)-*β*-glucoside	620.2 ($C_{29}H_{32}O_{15}$)
2	2-Methyl-1,3,6-trihydroxy-9,10-anhthraquinone 3-*O*-*α*-rhamnosyl(1→2)-*β*-glucoside	578.2 ($C_{27}H_{30}O_{14}$)
3	Munjistine	284.0 ($C_{15}H_{18}O_6$)
4	2-Methyl-1,3,6-trihydroxy-9,10-anhthraquinone 3-*O*-(6'-*O*-acetyl)-*β*-glucoside	474.1 ($C_{23}H_{22}O_{11}$)
5	2-Methyl-1,3,6-trihydroxy-9,10-anhthraquinone	270.0 ($C_{15}H_{10}O_5$)
6	3,6-Dihydroxy-2-hydroxymethyl-9,10-anthraquinone	270.0 ($C_{15}H_{10}O_5$)
7	3,6-Dihydroxy-2-methyl-9,10-anthraquinone	254.0 ($C_{15}H_{10}O_4$)
8	1,3,6-Trihydroxy-9,10-anthraquinone	256.0 ($C_{14}H_8O_5$)
9	Alizarin	240.0 ($C_{14}H_8O_4$)
10	Rubiadin	254.0 ($C_{15}H_{10}O_4$)
11	Xanthopurpurin	240.0 ($C_{14}H_8O_4$)
12	Lucidin-ω-methyl ether	284.1 ($C_{16}H_{12}O_5$)

13	Digiferrol	270.0 ($C_{15}H_{10}O_5$)
14	Lucidin-ω-ethyl ether	298.1 ($C_{17}H_{14}O_5$)
15	3-Acetylrubianol-c	558.4 ($C_{34}H_{54}O_6$)
16	28-Deoxyrubiarbonone E	454.3 ($C_{30}H_{46}O_3$)
17	19-Dedihydrorubiarbonol B	456.4 ($C_{30}H_{48}O_3$)
18	3-*O*-acetylrubiarbonol B	500.4 ($C_{32}H_{52}O_4$)
19	3-*O*-acetylrubiarbonol A	516.4 ($C_{32}H_{52}O_5$)
20	Rubiarbonol A	474.4 ($C_{30}H_{50}O_4$)
21	Rubiarbonol B	458.4 ($C_{30}H_{50}O_3$)
22	Rubiarbonone D	456.4 ($C_{30}H_{48}O_3$)
23	Rubiarbonone B	456.4 ($C_{30}H_{48}O_3$)
24	Rubiarbonone C	514.4 ($C_{32}H_{50}O_5$)
25	Rubianol C	516.4 ($C_{32}H_{52}O_5$)
26	3*β*-*O*-acetyl-16*β* -hydroxy-12-oxooleane	500.4 ($C_{32}H_{52}O_4$)
27	Maslinic acid	472.3 ($C_{30}H_{48}O_4$)
28	Isolariciresinol	360.1 ($C_{20}H_{24}O_6$)

29	Lariciresinol	360.1 ($C_{20}H_{24}O_6$)
30	Syringaresinol	418.2 ($C_{22}H_{26}O_8$)
31	Pinoresinol	358.1 ($C_{20}H_{22}O_6$)
32	Tectoquinone	222.1 ($C_{15}H_{10}O_2$)
33	1-Hydroxy-2-methyl-9,10-anthraquinone	238.1($C_{15}H_{10}O_3$)

Supplementary Figure Legends

Supplementary Figure 1. NMR and LC/MS data of 2-methyl-1,3,6-trihydroxy-9,10-anhthraquinone 3-*O*-(6'-*O*-acetyl)-*α*-rhamnosyl (1→2)-*β*-glucoside. (A) ^{1}H NMR spectrum (300 MHz, DMSO-*d6*). (B) ^{13}C NMR spectrum (150 MHz, DMSO-*d6*). (C) LC-MS analysis.

Supplementary Figure 2. NMR and LC/MS data of 2-methyl-1,3,6-trihydroxy-9,10-anhthraquinone 3-*O*-*α*-rhamnosyl(1→2)-*β*-glucoside. (A) ^{1}H NMR spectrum (600 MHz, DMSO-*d6*). (B) ^{13}C NMR spectrum (150 MHz, DMSO-*d6*). (C) LC-MS analysis.

Supplementary Figure 3. NMR and LC/MS data of munjistine. (A) ^{1}H NMR spectrum (300 MHz, DMSO-*d6*). (B) ^{13}C NMR spectrum (75 MHz, DMSO-*d6*). (C) LC-MS analysis.

Supplementary Figure 4. NMR and LC/MS data of 2-methyl-1,3,6-trihydroxy-9,10-anhthraquinone 3-*O*-(6'-*O*-acetyl)-*β*-glucoside. (A) ^{1}H NMR spectrum (300 MHz, DMSO-*d6*). (B) ^{13}C NMR spectrum (75 MHz, DMSO-*d6*). (C) LC-MS analysis.

Supplementary Figure 5. NMR and LC/MS data of 2-methyl-1,3,6-trihydroxy-9,10-anhthraquinone. (A) ^{1}H NMR spectrum (300 MHz, DMSO-*d6*). (B) ^{13}C NMR spectrum (75 MHz, DMSO-*d6*). (C) LC-MS analysis.

Supplementary Figure 6. NMR and LC/MS data of 3,6-dihydroxy-2-hydroxymethyl-9,10-anthraquinone. (A) ^{1}H NMR spectrum (300 MHz, DMSO-*d6*). (B) ^{13}C NMR spectrum (75 MHz, DMSO-*d6*). (C) LC-MS analysis.

Supplementary Figure 7. NMR data of 3,6-dihydroxy-2-methyl-9,10-anthraquinone. (A) ^{1}H NMR spectrum (300 MHz, DMSO-*d6*). (B) ^{13}C NMR spectrum (75 MHz, DMSO-*d6*).

Supplementary Figure 8. NMR and LC/MS data of 1,3,6-trihydroxy-9,10-anthraquinone. (A) ^{1}H NMR spectrum (300 MHz, DMSO-*d6*). (B) ^{13}C NMR spectrum (75 MHz, DMSO-*d6*). (C) LC-MS analysis.

Supplementary Figure 9. NMR and LC/MS data of alizarin. (A) ^{1}H NMR spectrum (300 MHz, DMSO-*d6*). (B) ^{13}C NMR spectrum (150 MHz, DMSO-*d6*). (C) LC-MS analysis.

Supplementary Figure 10. NMR data of rubiadin. (A) ^{1}H NMR spectrum (300 MHz, DMSO-*d6*). (B) ^{13}C NMR spectrum (75 MHz, DMSO-*d6*).

Supplementary Figure 11. NMR and LC/MS data of xanthopurpurin. (A) ^{1}H NMR spectrum (300 MHz, DMSO-*d6*). (B) ^{13}C NMR spectrum (75 MHz, DMSO-*d6*). (C) LC-MS analysis.

Supplementary Figure 12. NMR and LC/MS data of lucidin-ω-methyl ether. (A) ^{1}H NMR spectrum (300 MHz, $CDCl_3$). (B) ^{13}C NMR spectrum (75 MHz, $CDCl_3$). (C) LC-MS analysis.

Supplementary Figure 13. NMR and LC/MS data of digiferrol. (A) ^{1}H NMR spectrum (300 MHz, DMSO-*d6*). (B) ^{13}C NMR spectrum (75 MHz, DMSO-*d6*). (C) LC-MS analysis.

Supplementary Figure 14. NMR and LC/MS data of lucidin-ω-ethyl ether. (A) ^{1}H NMR spectrum (300 MHz, $CDCl_3$). (B) ^{13}C NMR spectrum (75 MHz, $CDCl_3$). (C) LC-MS analysis.

Supplementary Figure 15. NMR and HR-ESI-MS data of 3-acetylrubianol-c. (A) ^{1}H NMR spectrum (600 MHz, $CDCl_3$). (B) ^{13}C NMR spectrum (150 MHz, $CDCl_3$). (C) HR-ESI-MS analysis.

Supplementary Figure 16. NMR and HR-ESI-MS data of 28-deoxyrubiarbonone E. (A) ^{1}H NMR spectrum (600 MHz, $CDCl_3$). (B) ^{13}C NMR spectrum (150 MHz, $CDCl_3$). (C) HR-ESI-MS analysis.

Supplementary Figure 17. NMR and HR-ESI-MS data of 19-dedihydrorubiarbonol B. (A) ^{1}H NMR spectrum (600 MHz, $CDCl_3$). (B) ^{13}C NMR spectrum (150 MHz, $CDCl_3$). (C) HR-ESI-MS analysis.

Supplementary Figure 18. NMR and HR-ESI-MS data of 3-*O*-acetylrubiarbonol B. (A) ^{1}H NMR spectrum (600 MHz, $CDCl_3$). (B) ^{13}C NMR spectrum (150 MHz, $CDCl_3$). (C) HR-ESI-MS analysis.

Supplementary Figure 19. NMR and HR-ESI-MS data of 3-*O*-acetylrubiarbonol A. (A) ^{1}H NMR spectrum (600 MHz, C_5D_5N). (B) ^{13}C NMR spectrum (150 MHz, C_5D_5N). (C) HR-ESI-MS analysis.

Supplementary Figure 20. NMR and LC/MS data of rubiarbonol A. (A) ^{1}H NMR spectrum (600 MHz, C_5D_5N). (B) ^{13}C NMR spectrum (150 MHz, C_5D_5N). (C) LC-MS analysis.

Supplementary Figure 21. NMR data of rubiarbonol B. (A) ^{1}H NMR spectrum (300 MHz, C_5D_5N). (B) ^{13}C NMR spectrum (75 MHz, C_5D_5N).

Supplementary Figure 22. NMR and LC/MS data of rubiarbonone D. (A) ^{1}H NMR spectrum (300 MHz, $CDCl_3$). (B) ^{13}C NMR spectrum (75 MHz, $CDCl_3$). (C) LC-MS analysis.

Supplementary Figure 23. NMR and LC/MS data of rubiarbonone B. (A) ^{1}H NMR spectrum (300 MHz, MeOD). (B) ^{13}C NMR spectrum (75 MHz, MeOD). (C) LC-MS analysis.

Supplementary Figure 24. NMR and LC/MS data of rubiarbonone C. (A) ^{1}H NMR spectrum (300 MHz, MeOD). (B) ^{13}C NMR spectrum (75 MHz, MeOD). (C) LC-MS analysis.

Supplementary Figure 25. NMR and LC/MS data of rubianol C. (A) ^{1}H NMR spectrum (300 MHz, MeOD). (B) ^{13}C NMR spectrum (75 MHz, MeOD). (C) LC-MS analysis.

Supplementary Figure 26. NMR and LC/MS data of 3β-O-acetyl-16β-hydroxy-12-oxooleane. (A) ^{1}H NMR spectrum (300 MHz, $CDCl_3$). (B) ^{13}C NMR spectrum (75 MHz, $CDCl_3$). (C) LC-MS analysis.

Supplementary Figure 27. NMR and LC/MS data of maslinic acid. (A) ^{1}H NMR spectrum (300 MHz, C_5D_5N). (B) ^{13}C NMR spectrum (75 MHz, C_5D_5N). (C) LC-MS analysis.

Supplementary Figure 28. NMR and LC/MS data of isolariciresinol. (A) ^{1}H NMR spectrum (300 MHz, MeOD). (B) ^{13}C NMR spectrum (75 MHz, MeOD). (C) LC-MS analysis.

Supplementary Figure 29. NMR and LC/MS data of lariciresinol. (A) ^{1}H NMR spectrum (300 MHz, MeOD). (B) ^{13}C NMR spectrum (75 MHz, MeOD). (C) LC-MS analysis.

Supplementary Figure 30. NMR and LC/MS data of syringaresinol. (A) ^{1}H NMR spectrum (300 MHz, MeOD). (B) ^{13}C NMR spectrum (75 MHz, MeOD). (C) LC-MS analysis.

Supplementary Figure 31. NMR and LC/MS data of pinoresinol. (A) ^{1}H NMR spectrum (300 MHz, $CDCl_3$). (B) ^{13}C NMR spectrum (75 MHz, $CDCl_3$). (C) LC-MS analysis.

Supplementary Figure 32. NMR and LC/MS data of tectoquinone. (A) ^{1}H NMR spectrum (300 MHz, $CDCl_3$). (B) ^{13}C NMR spectrum (75 MHz, $CDCl_3$). (C) LC-MS analysis.

Supplementary Figure 33. NMR and LC/MS data of 1-hydroxy-2-methyl-9,10-

anthraquinone. (A) ^{1}H NMR spectrum (300 MHz, $CDCl_3$). (B) ^{13}C NMR spectrum (75 MHz, $CDCl_3$). (C) LC-MS analysis.

Supplementary Figure 34. Effects of pinoresinol on the cell cycle distribution in LN428 cells. LN428 were treated with PINO (0.5 μM) for the indicated times. Cells were stained with propidium iodide, and subjected to flow cytometry.

Supplementary Figure 35. Full-length immunoblots of Fig. 2F. (A) Pro-form and cleaved forms of caspase-8. (B) Pro-form and cleaved forms of caspase-9. (C) Pro-form and cleaved forms of caspase-3. (D) Pro-form and cleaved forms of PARP. (E) Actin.

Supplementary Figure 36. Full-length immunoblots of Fig. 3E. (A) p53. (B) p21. (C) Actin.

Supplementary Figure 37. Full-length immunoblots of Fig. 4A. (A) DR4. (B) DR5. (C) FADD. (D) Pro-form and cleaved forms of RIP1. (E) TRAF2. (F) $cFLIP_L$ (G) Survivin. (H) XIAP. (I) cIAP1/2. (J) Bcl-XS/L. (K) Pro-form and truncated forms of Bid. (L) Actin.

Supplementary Figure 38. Full-length immunoblots of Fig. 4B. (A) cFLIP. (B) Actin.

Supplementary Figure 39. Full-length immunoblots of Fig. 4D. (A) $cFLIP_L$ (B) Survivin. (C) Actin.

Supplementary Figure 40. Full-length immunoblots of Fig. 5B. (A) Flag-tagged $cFLIP_L$ (B) Flag-tagged survivin. (C) Pro-form and cleaved forms of caspase-8. (D) Pro-

form and cleaved forms of caspase-3. (E) Pro-form and cleaved forms of PARP. (F) Actin.

Supplementary Figure 41. Full-length immunoblots of Fig. 5C. (A) Pro-form and cleaved forms of $cFLIP_L$ in immunoprecipitates and whole cell lysates. (B) FADD in immunoprecipitates and whole cell lysates. (C) Pro-form and cleaved forms of caspase-10 in immunoprecipitates and whole cell lysates. (D) Pro-form and cleaved forms of caspase-8 in immunoprecipitates and whole cell lysates. (E) Actin in whole cell lysates.

Supplementary Figure 42. Full-length immunoblots of Fig. 6A. (A) HA-tagged ubiquitin in immunoprecipitates and whole cell lysates. (B) Flag-tagged $cFLIP_L$ in immunoprecipitates and whole cell lysates. (C) Actin in whole cell lysates.

Supplementary Figure 43. Full-length immunoblots of Fig. 6B. (A) $cFLIP_L$ (B) Survivin. (C) DR4. (D) DR5. (E) FADD. (F) RIP1. (G) TRAF2. (H) Actin.

Supplementary Figure 44. Full-length immunoblots of Fig. 6C.

Supplementary Figure 45. Full-length immunoblots of Fig. 6D.

Supplementary Figure 1

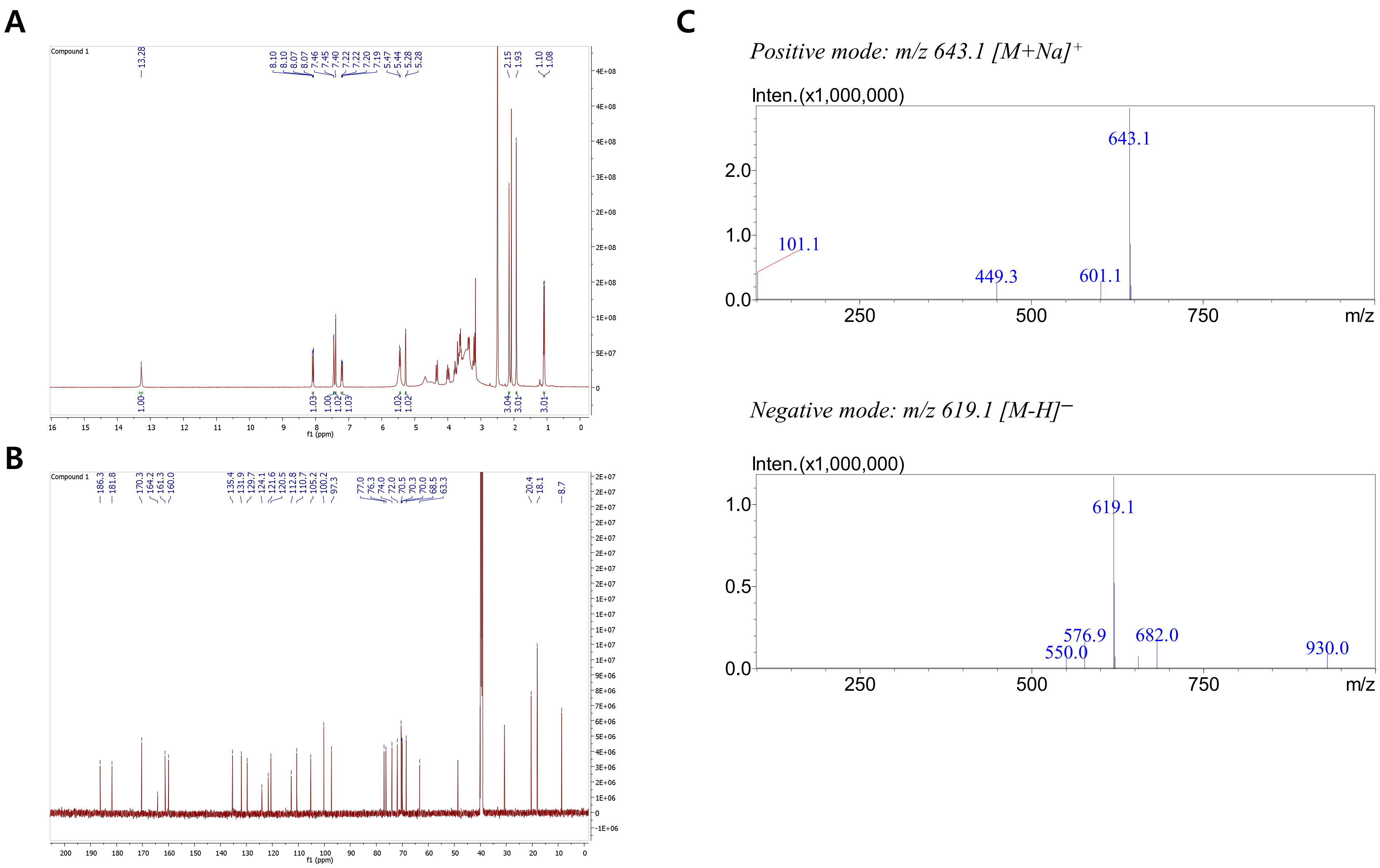

Supplementary Figure 2

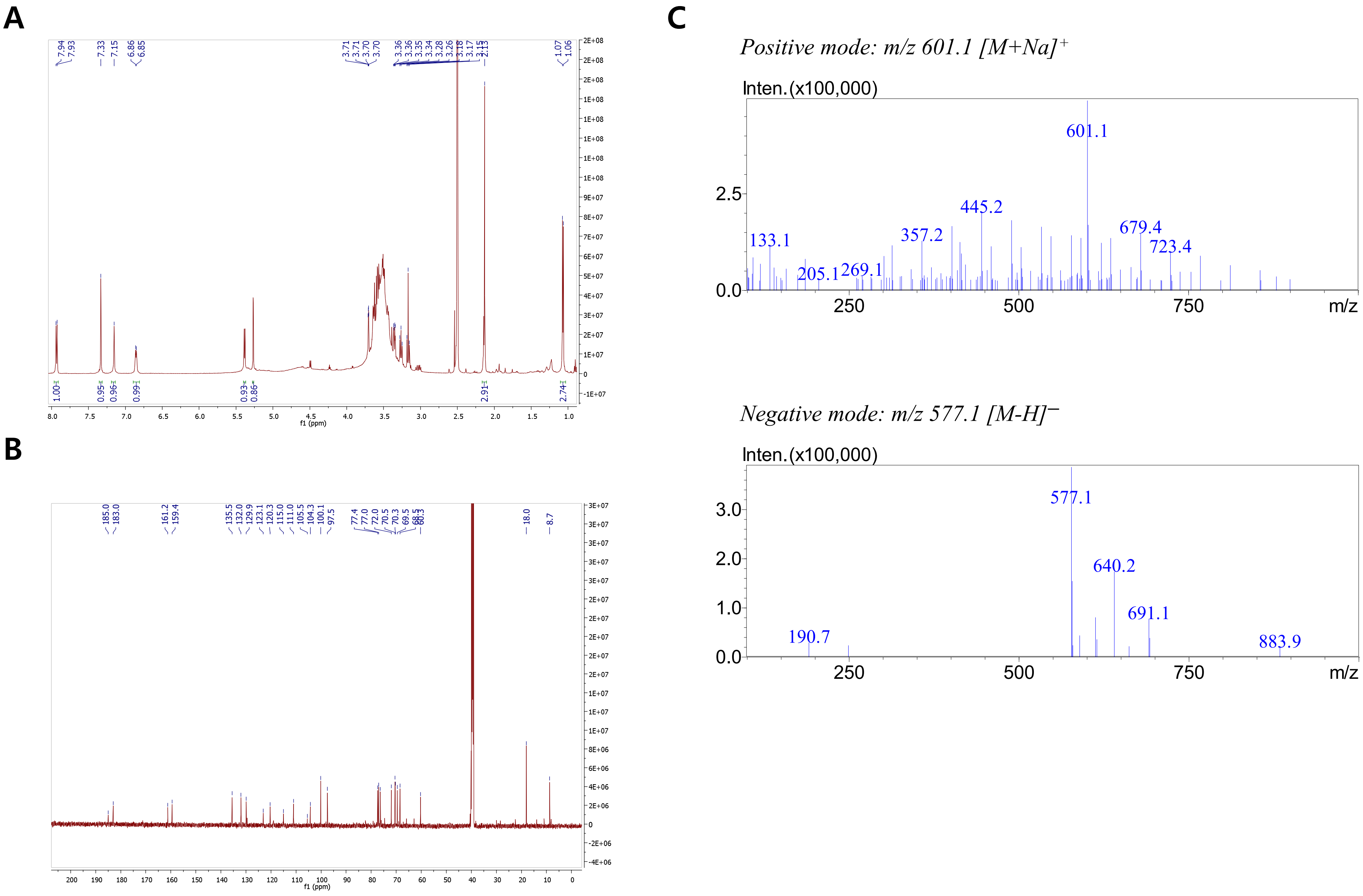

Supplementary Figure 3

A

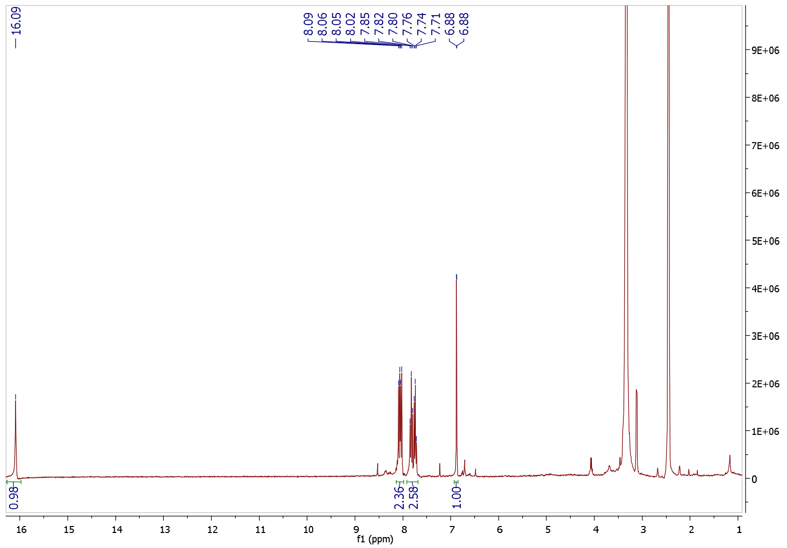

B

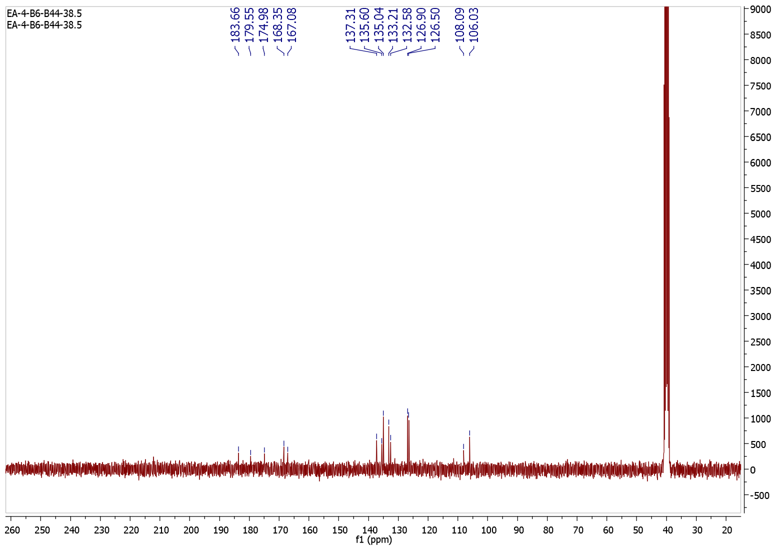

C

Positive mode: m/z 591.4 [2M+Na]$^+$

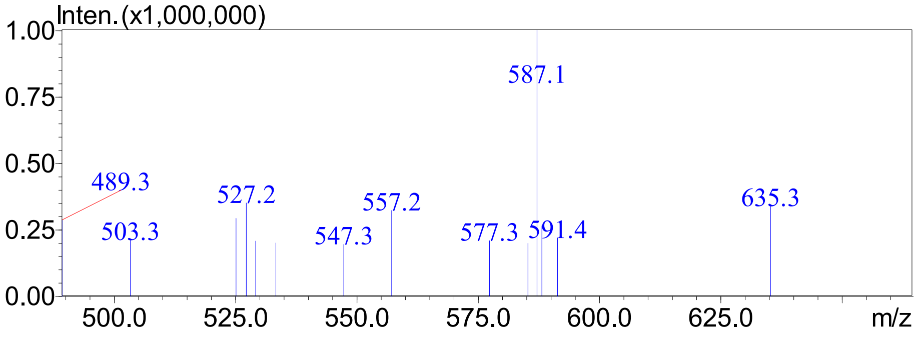

Negative mode: m/z 283.0 [M-H]$^-$

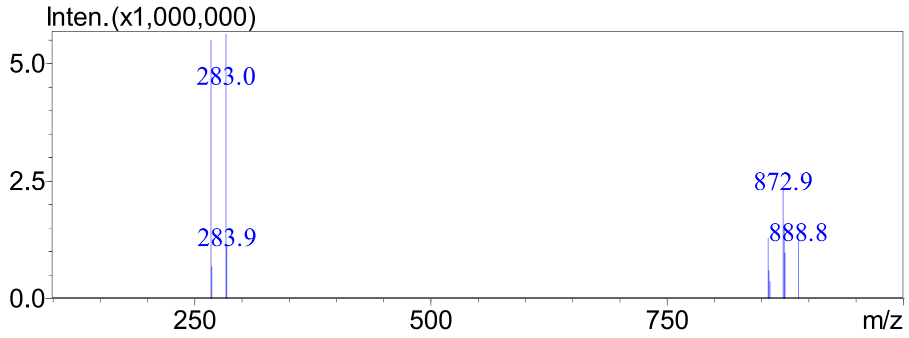

Supplementary Figure 4

A

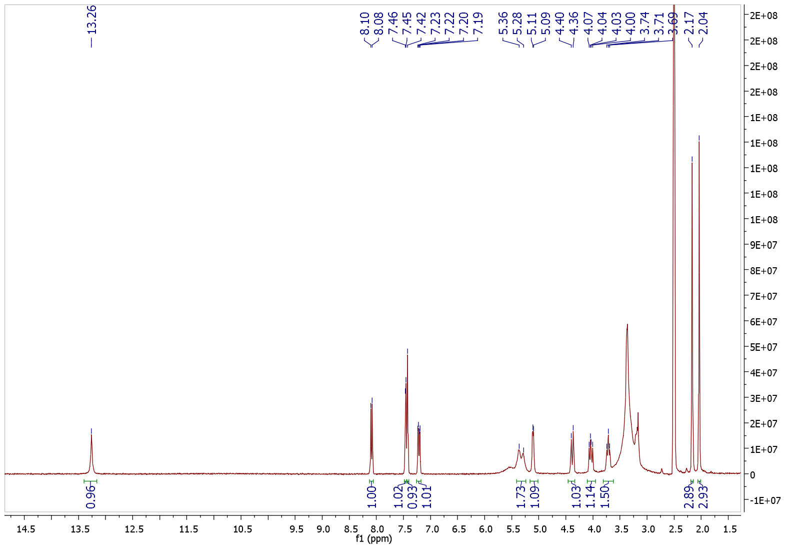

B

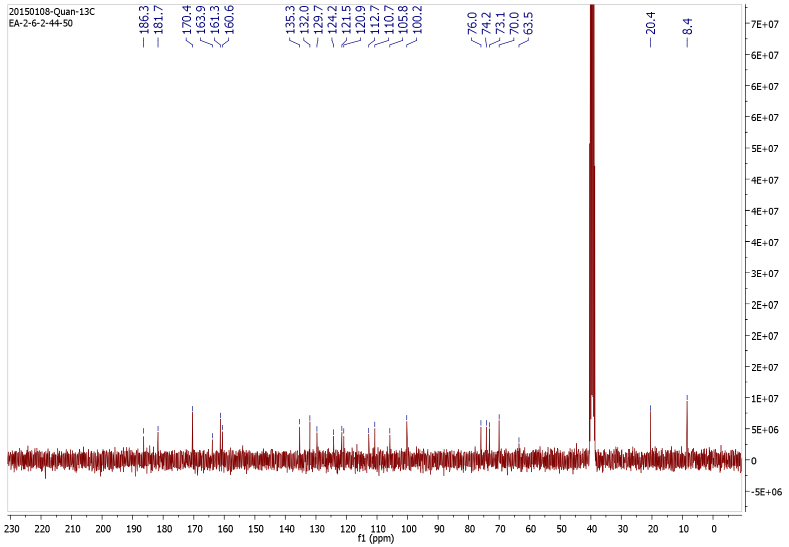

C

Positive mode: m/z 497.1 [M+Na]$^{+}$, 971.2 [2M+Na]$^{+}$

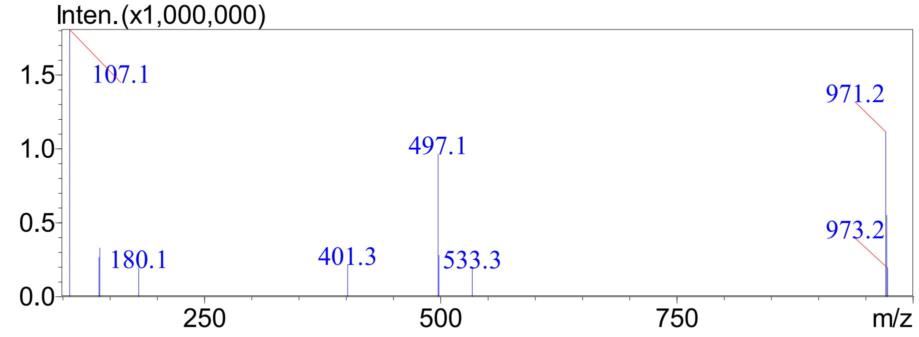

Negative mode: m/z 473.0 [M-H]$^{-}$, 983.1 [2M+Cl]$^{-}$

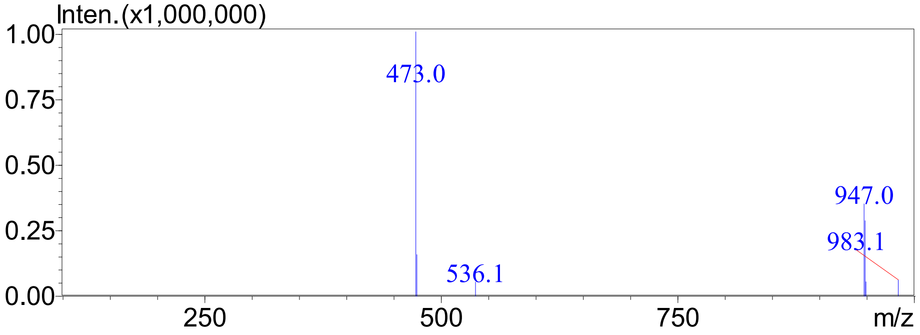

Supplementary Figure 5

A

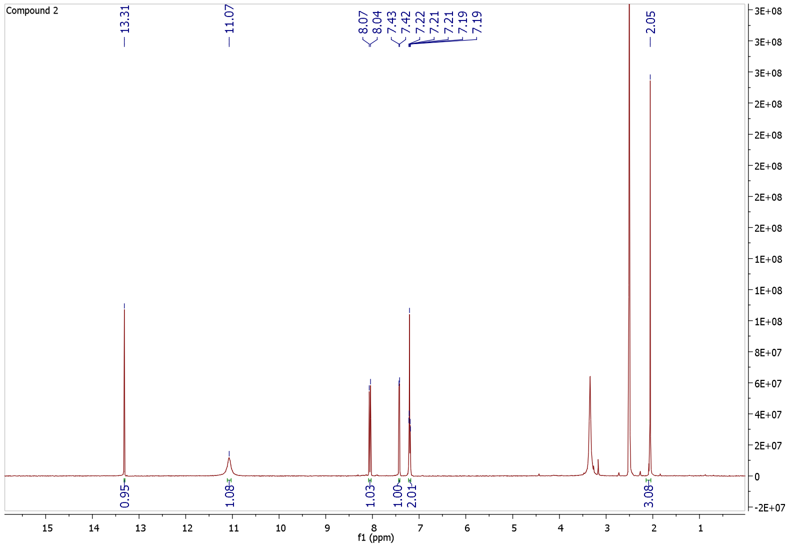

B

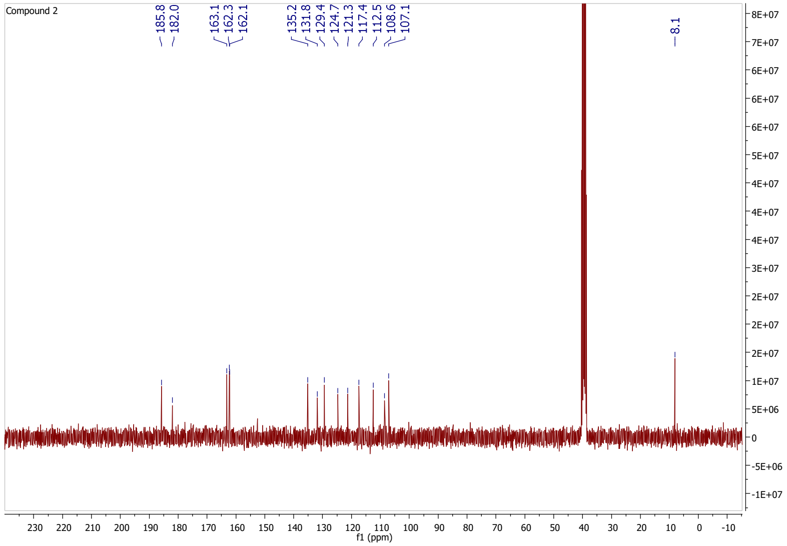

C

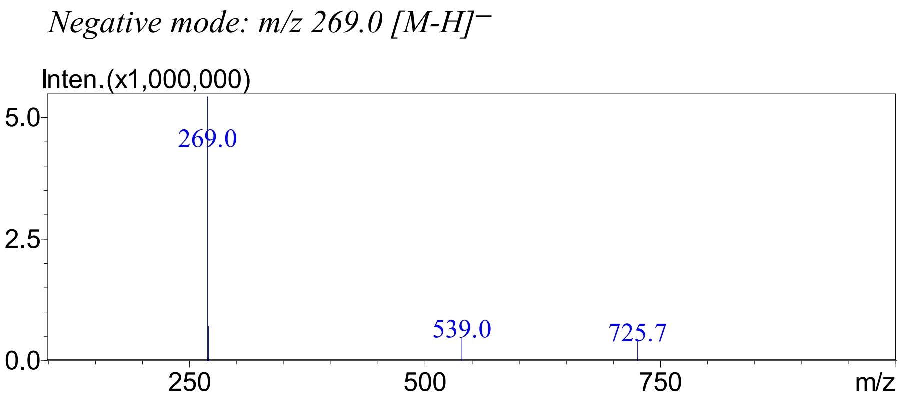

Supplementary Figure 6

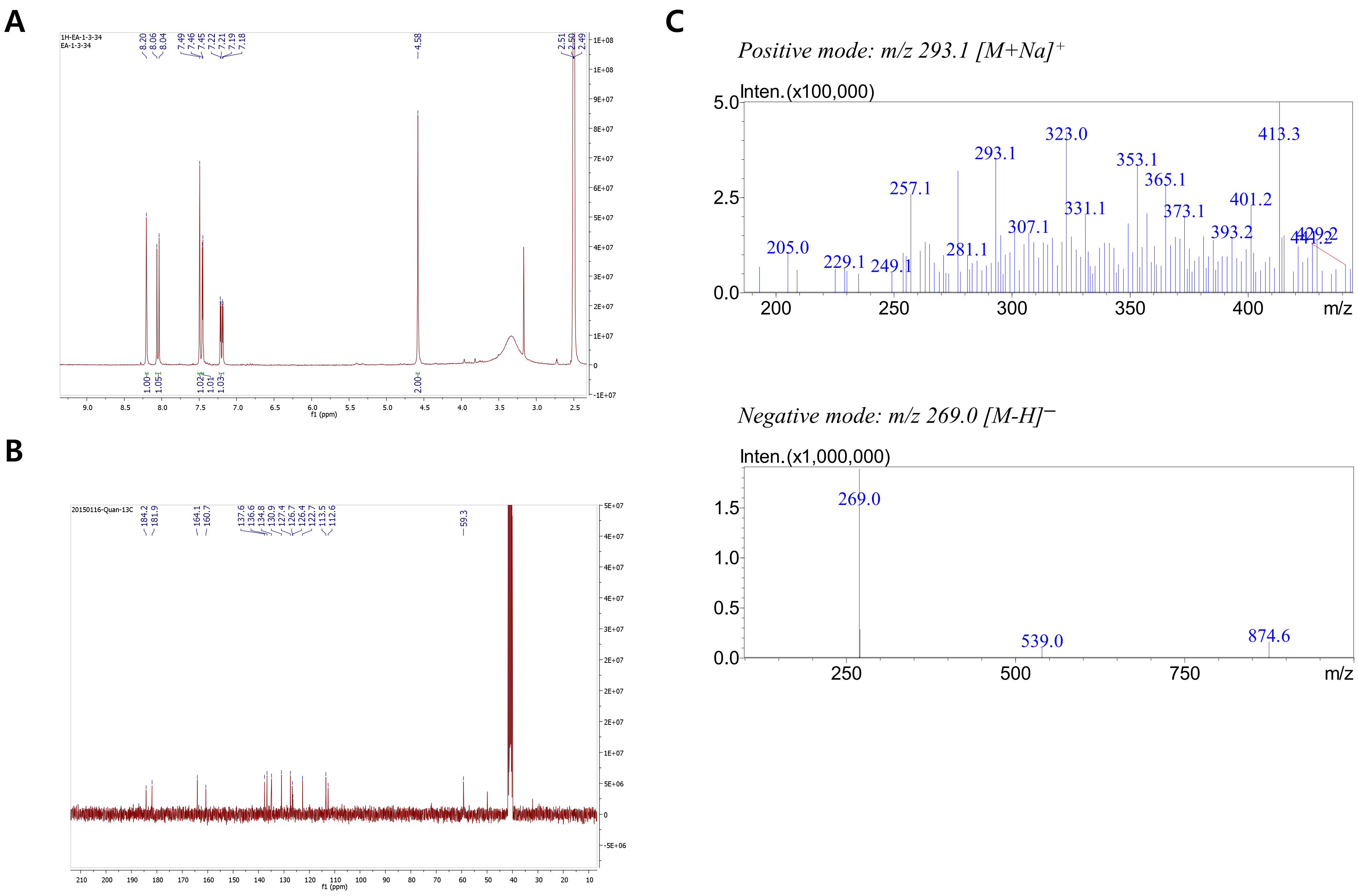

Supplementary Figure 7

A

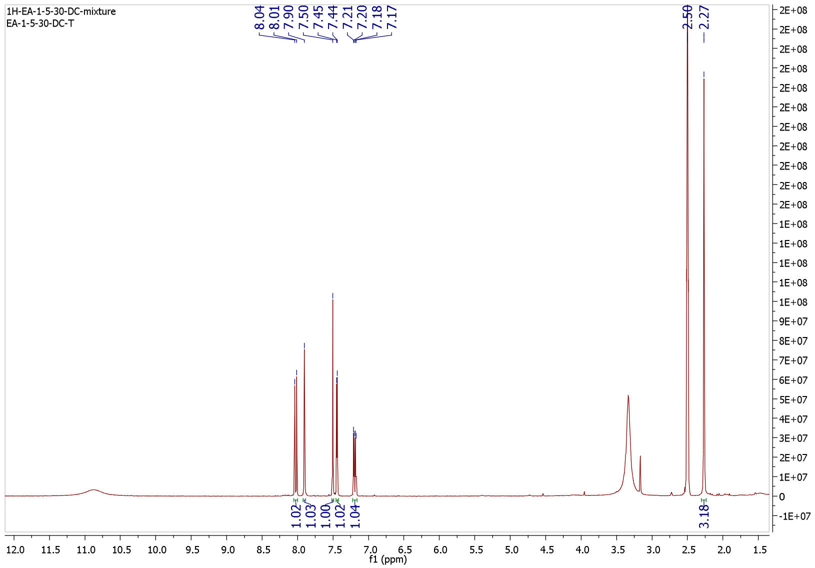

B

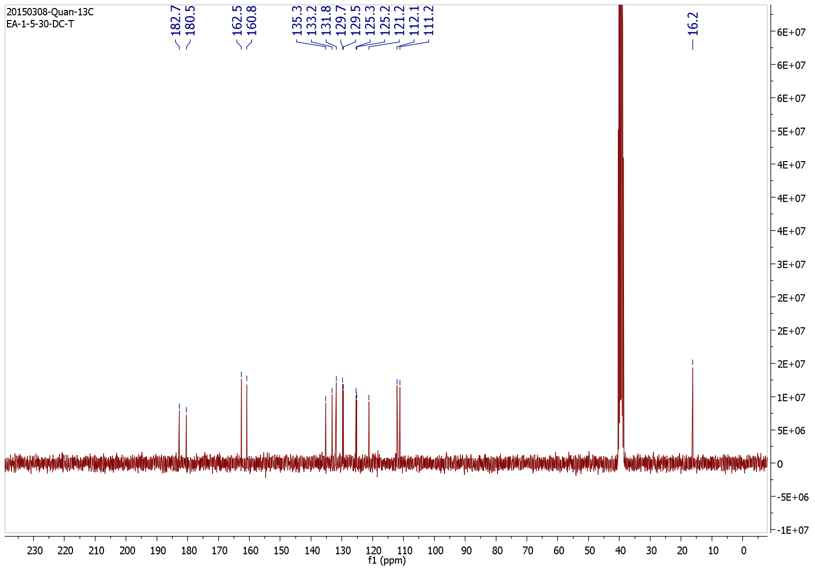

Supplementary Figure 8

A

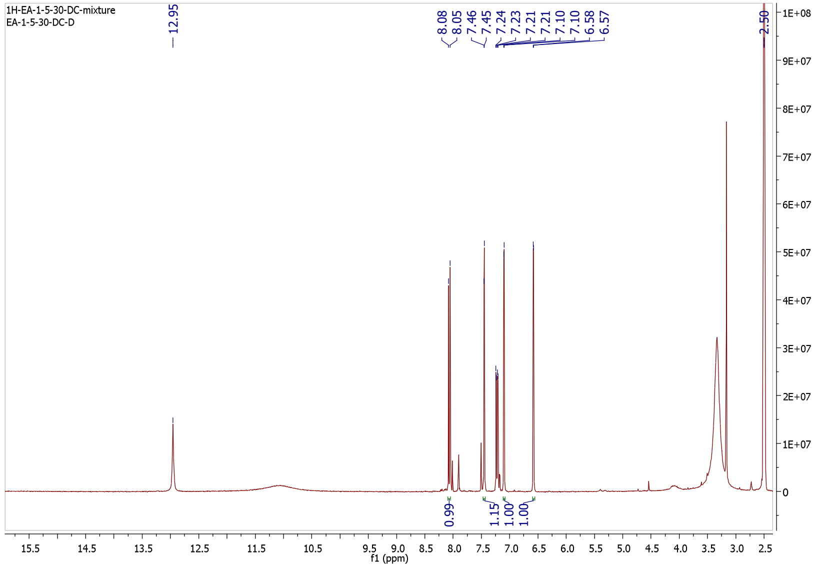

B

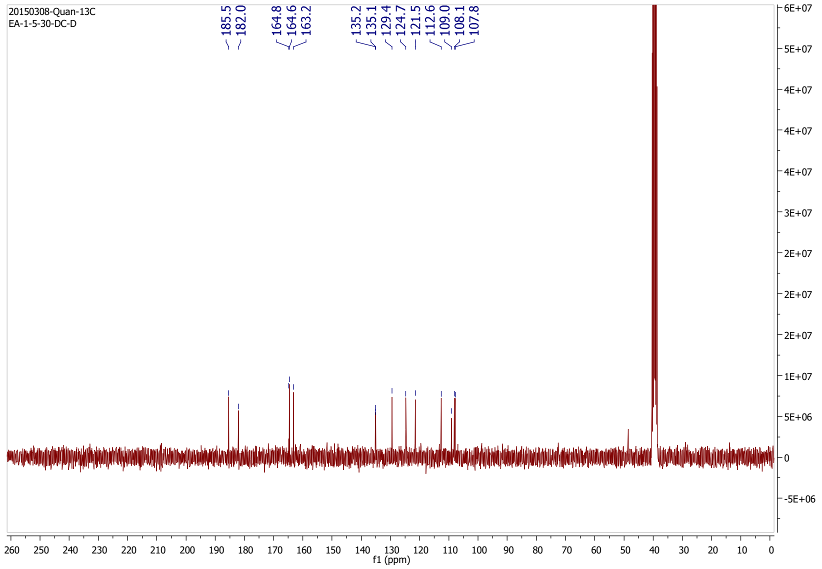

C

Negative mode: m/z 255.1 [M-H]⁻

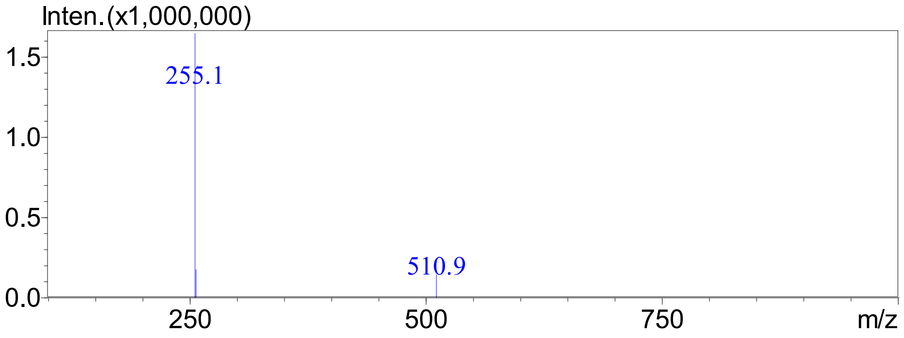

Supplementary Figure 9

A

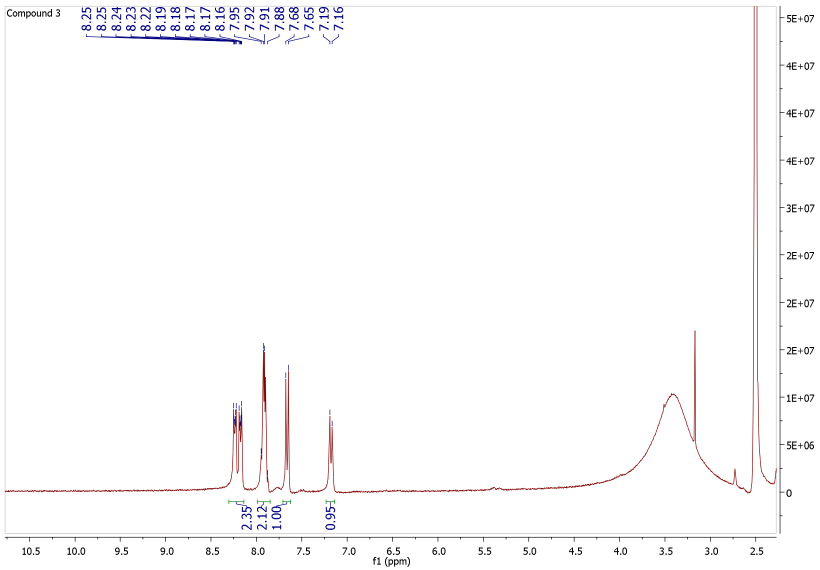

B

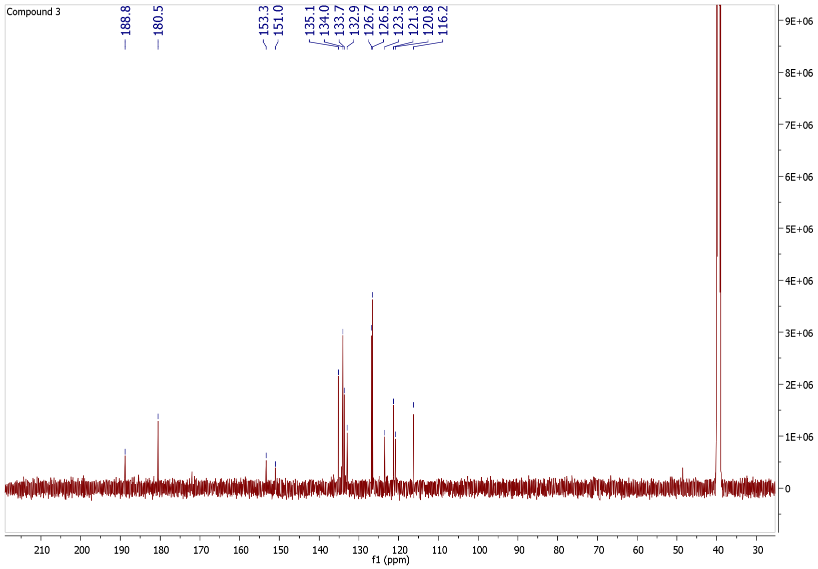

C

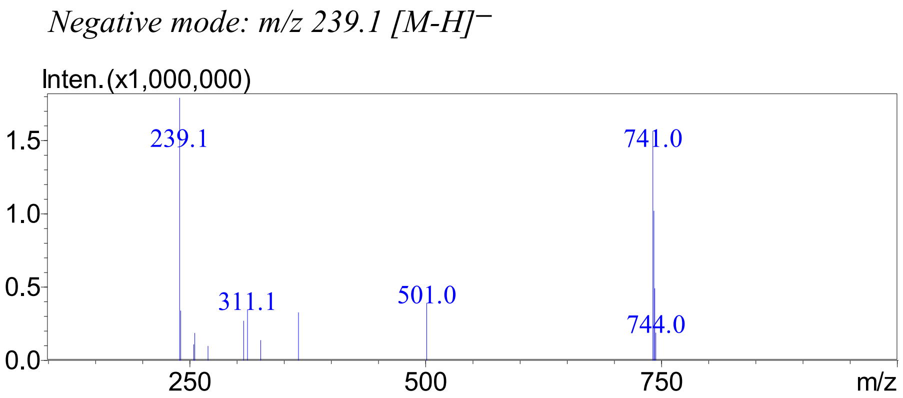

Supplementary Figure 10

A

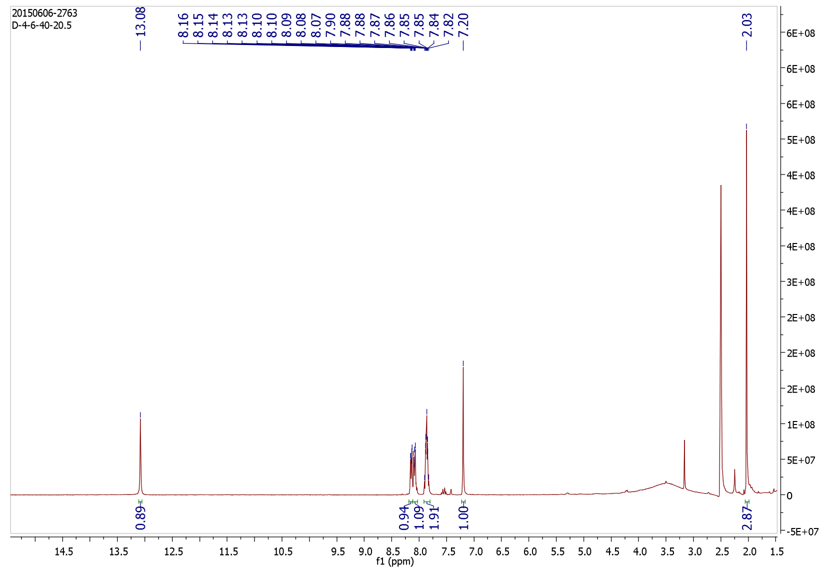

B

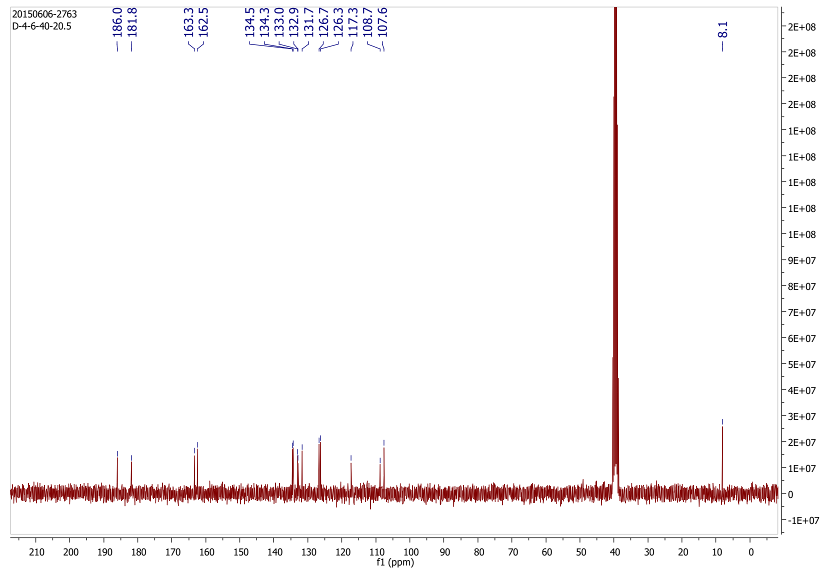

Supplementary Figure 11

A

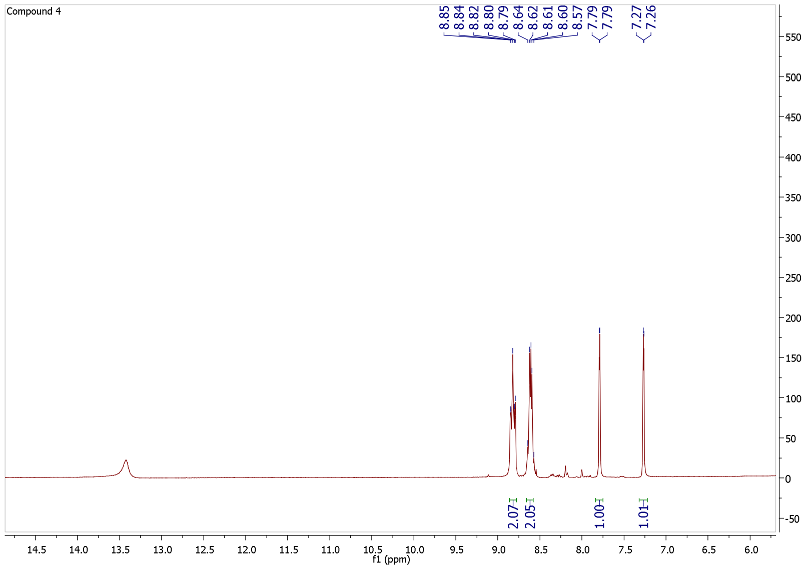

B

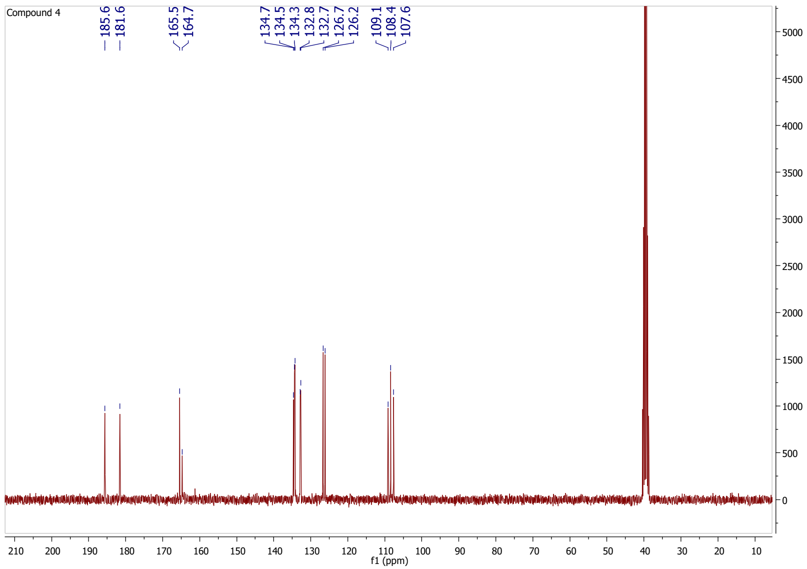

C

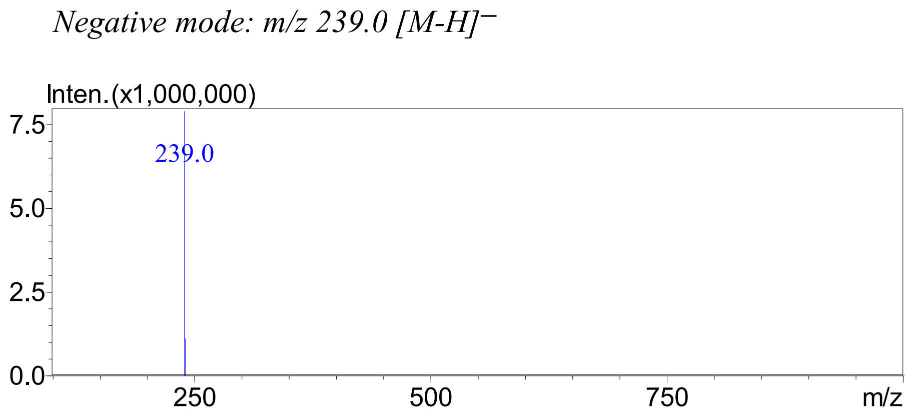

Supplementary Figure 12

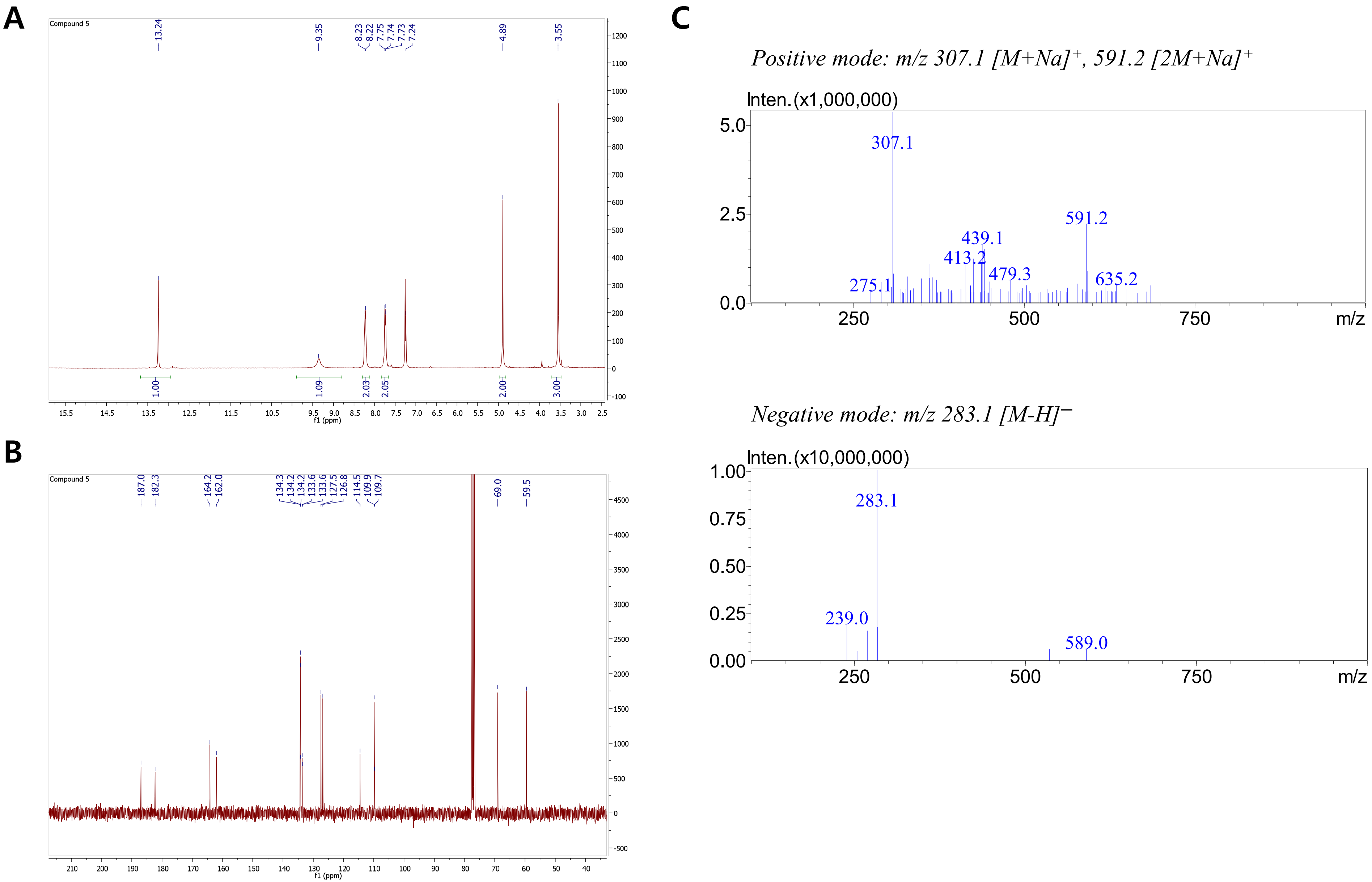

Supplementary Figure 13

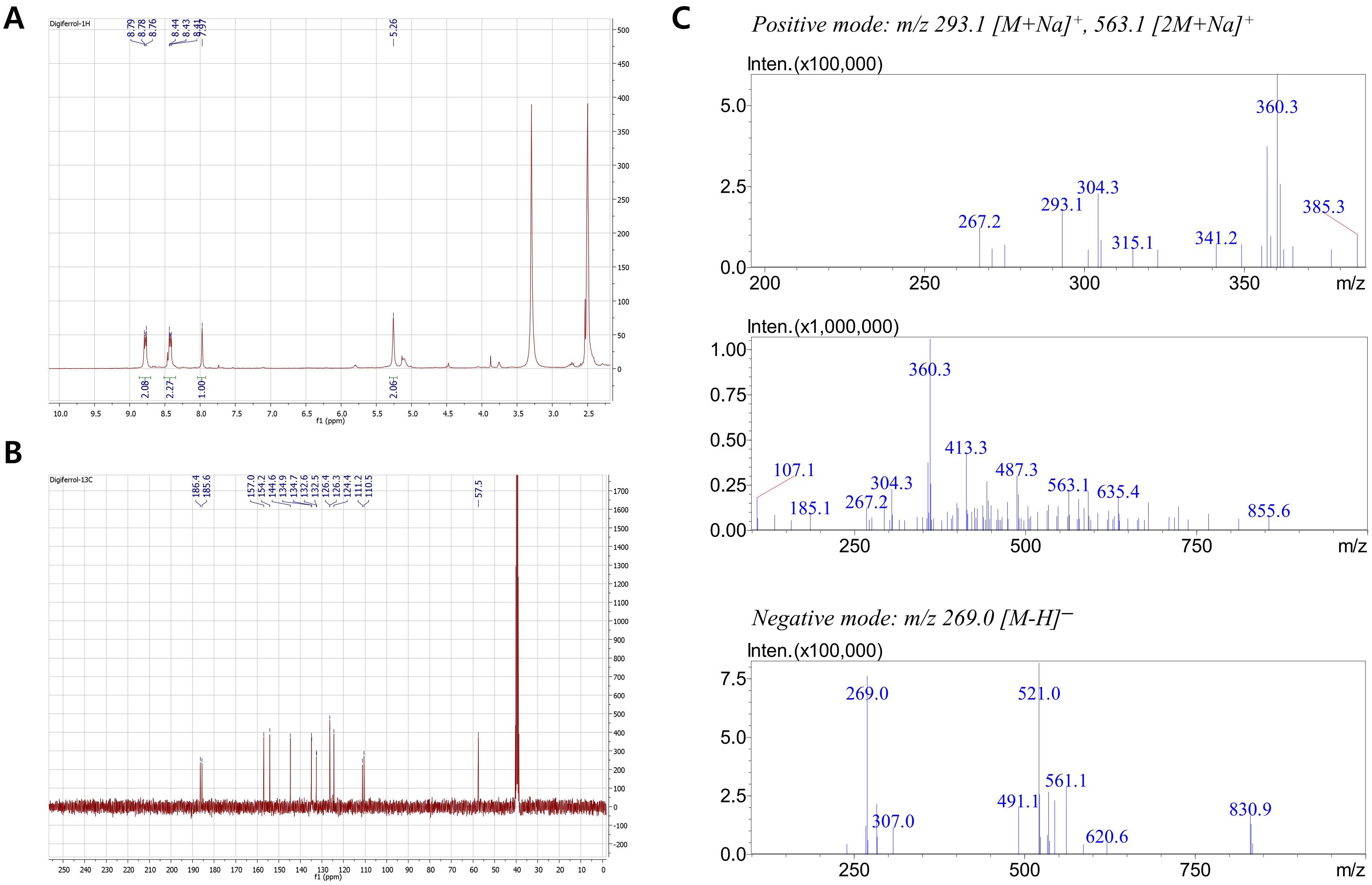

Supplementary Figure 14

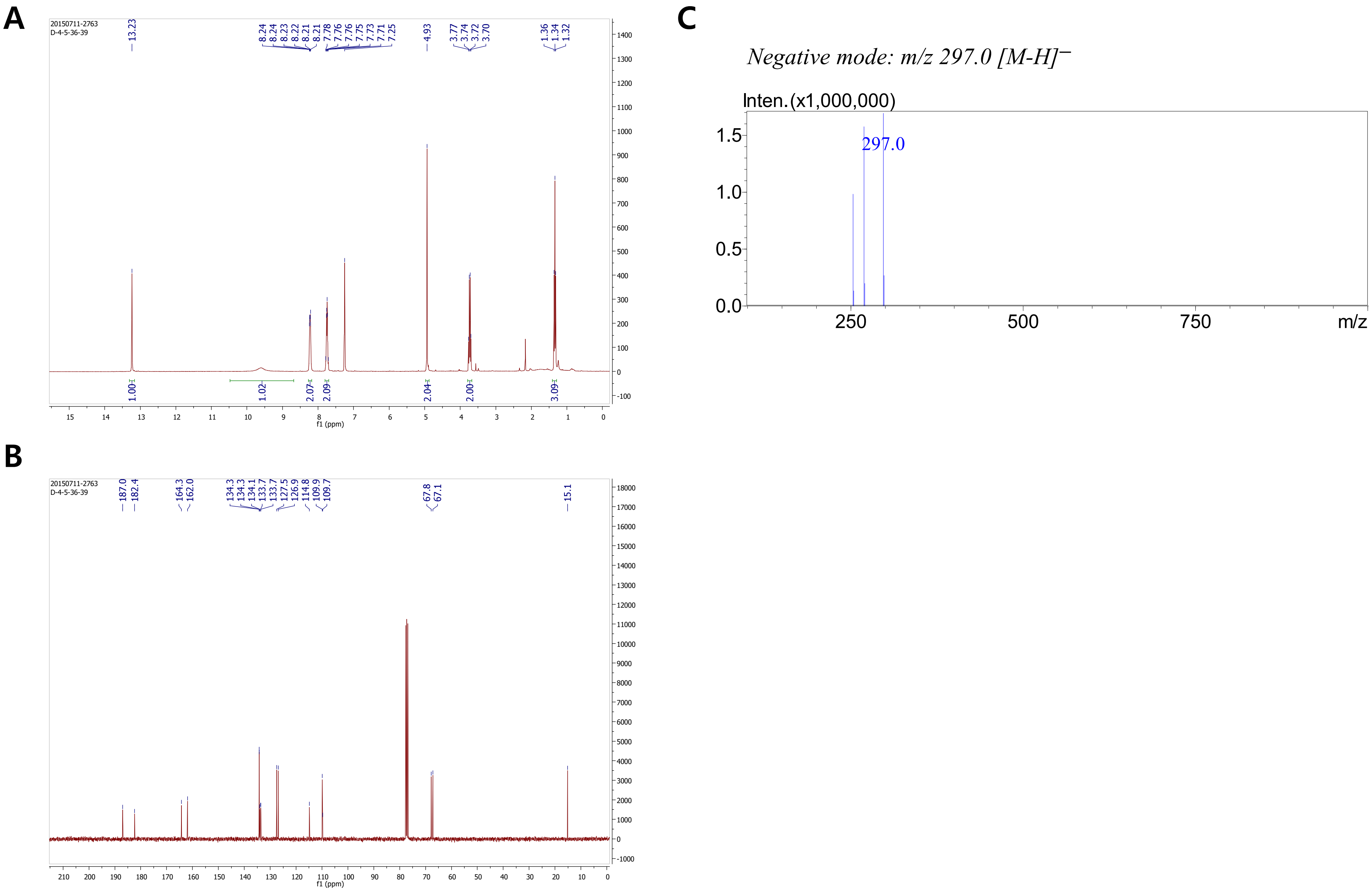

Supplementary Figure 15

A

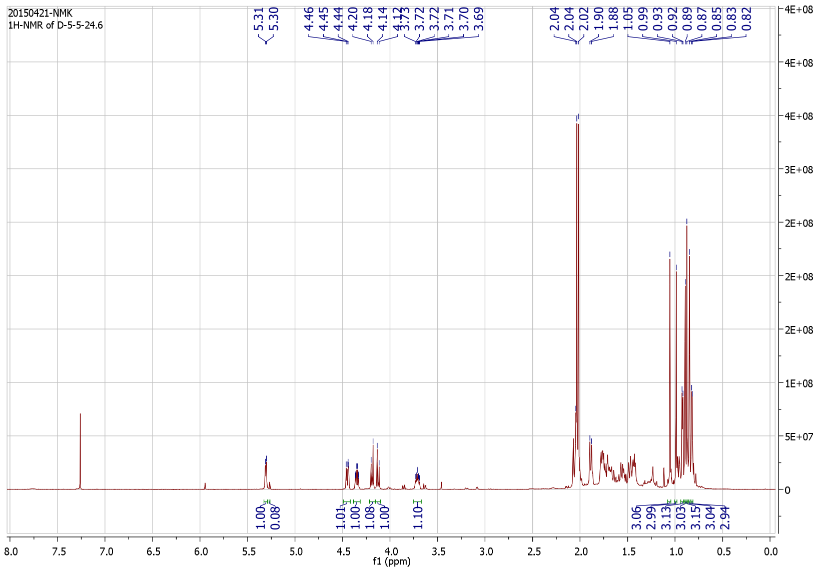

B

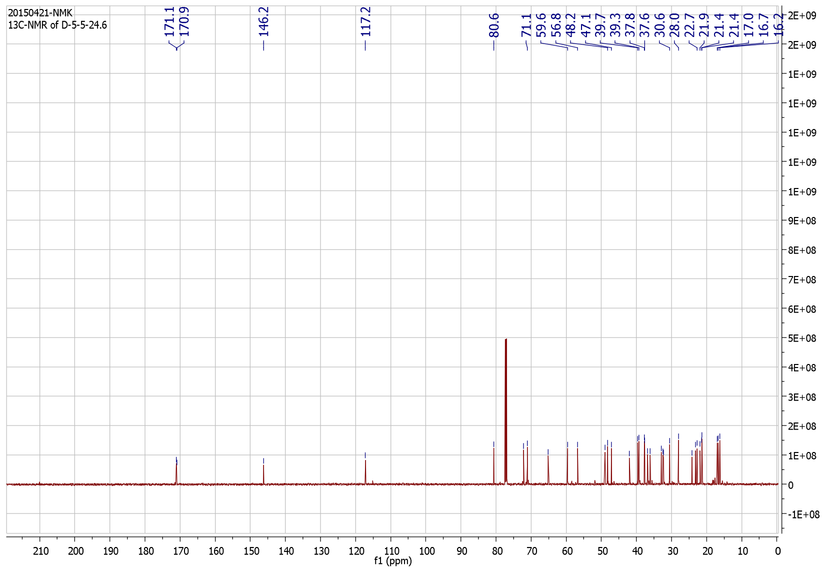

Supplementary Figure 15 (Continued)

C

Positive mode: m/z 581.3818 [M+Na]$^{+}$

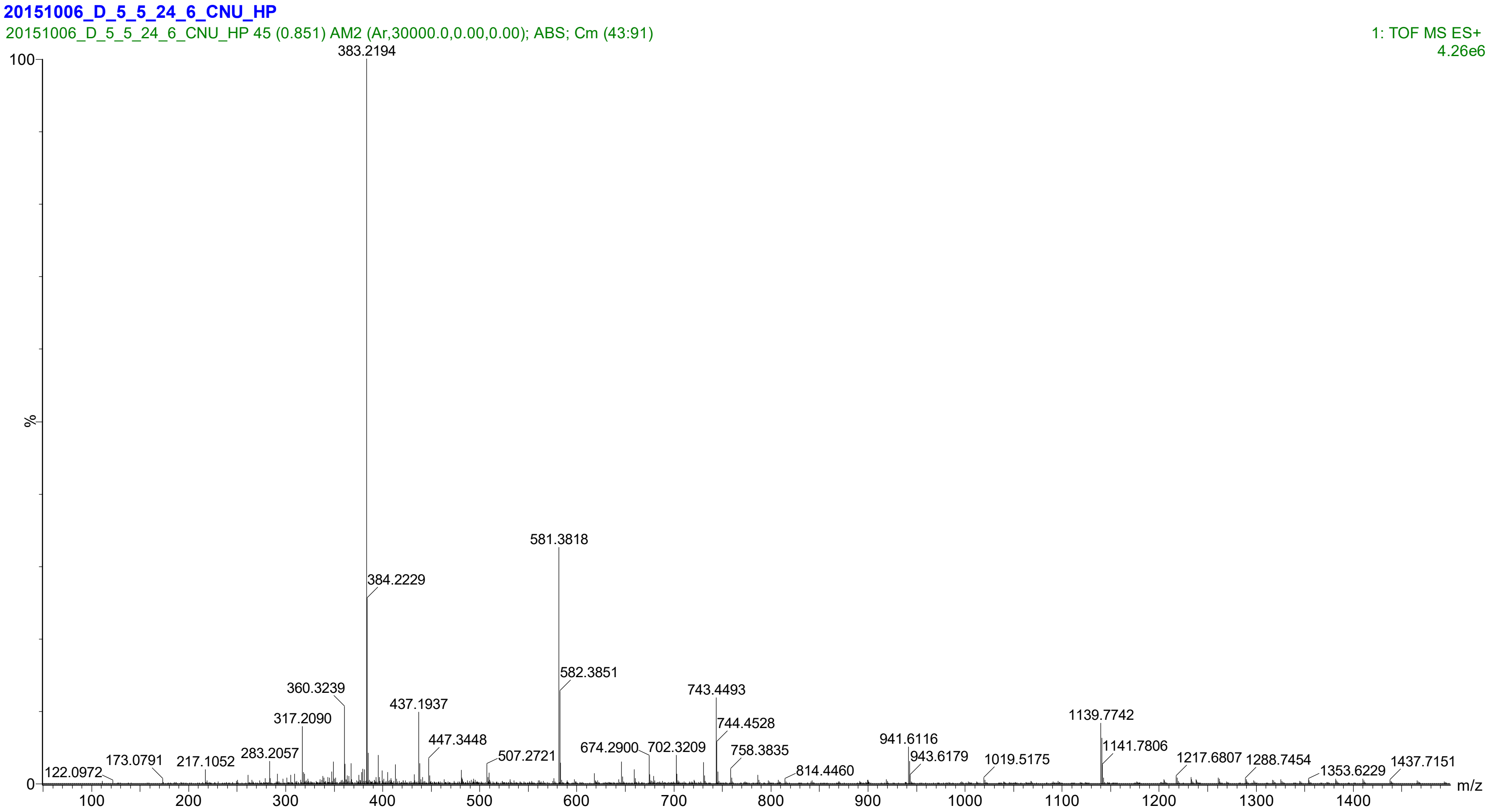

Supplementary Figure 16

A

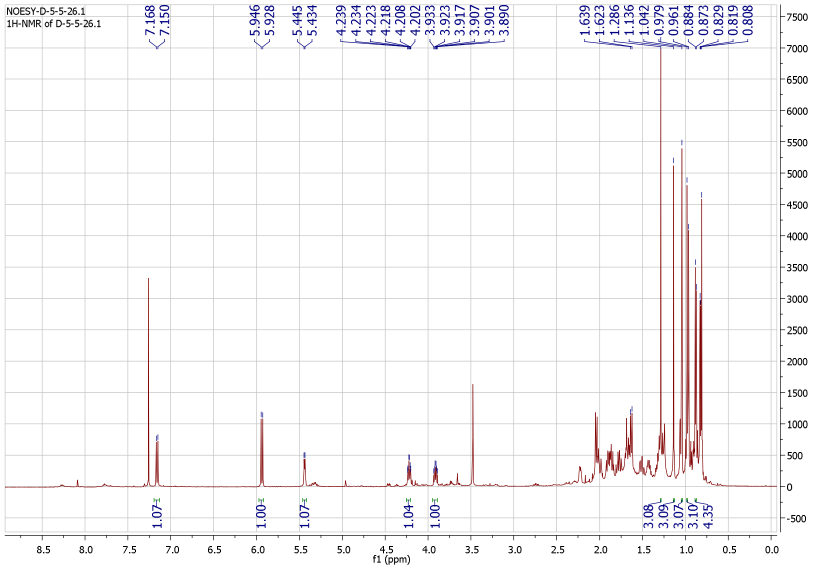

B

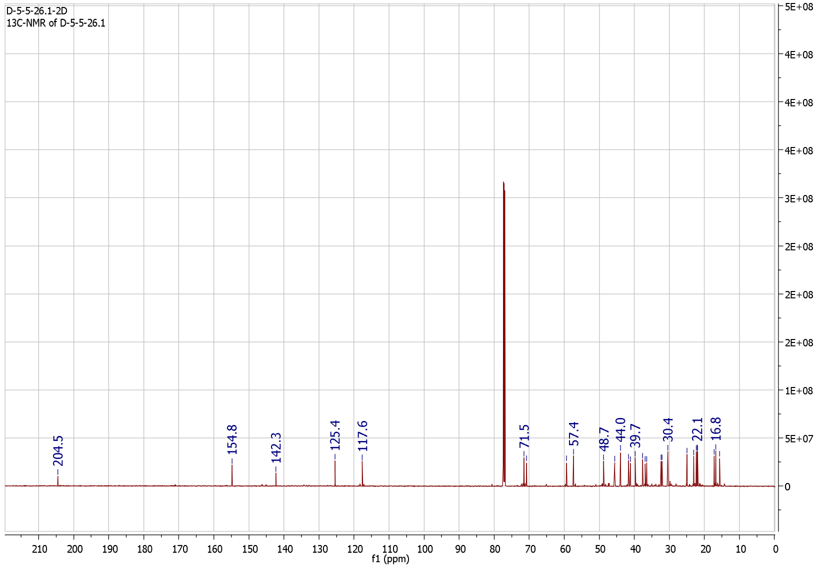

Supplementary Figure 16 (Continued)

C

Positive mode: m/z 477.3339 [M+Na]$^+$

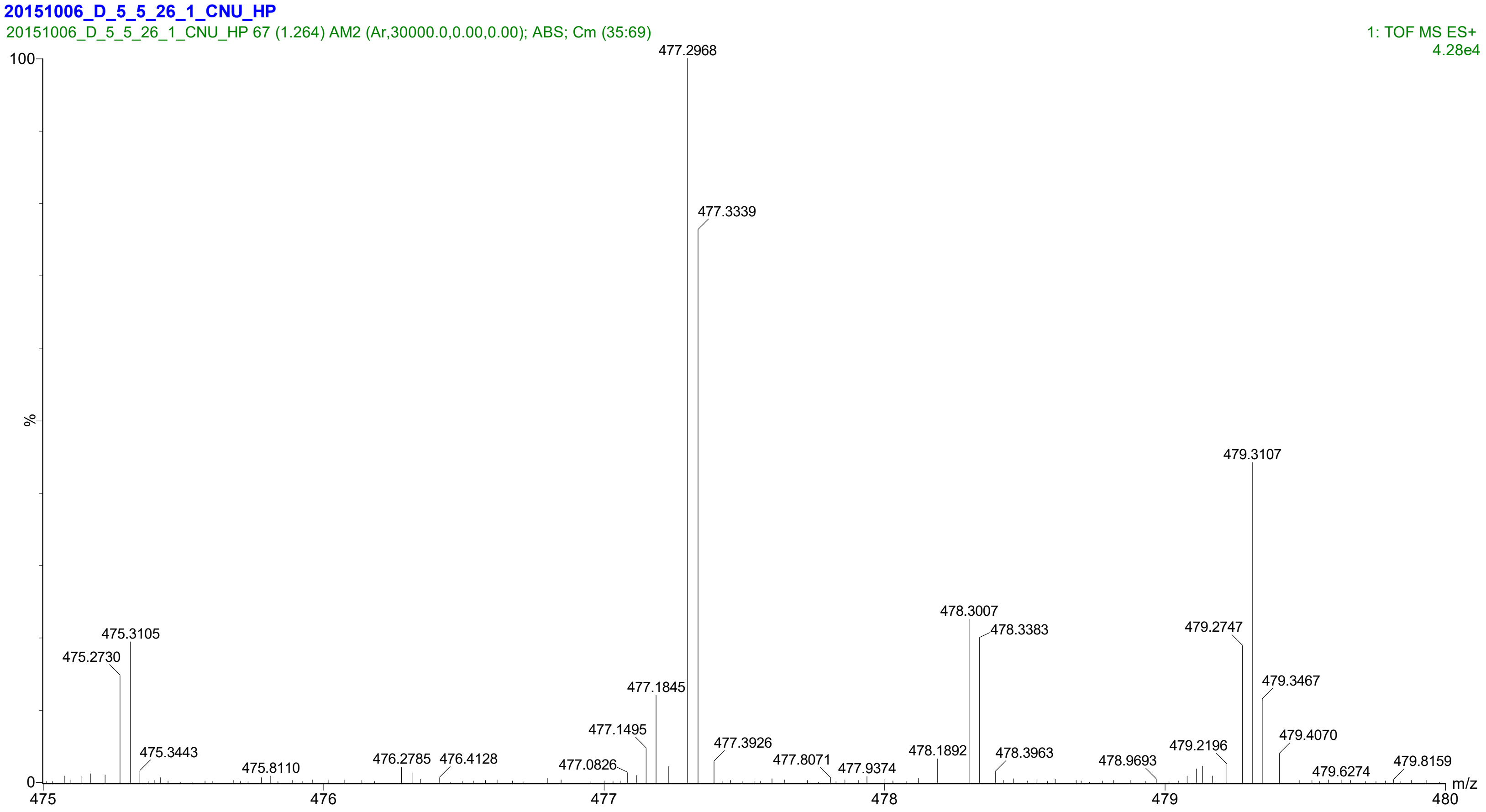

Supplementary Figure 17

A

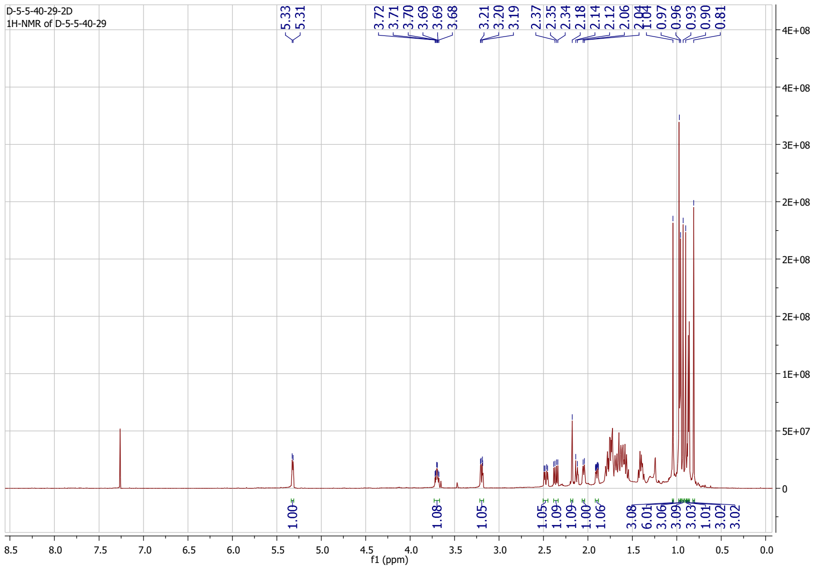

B

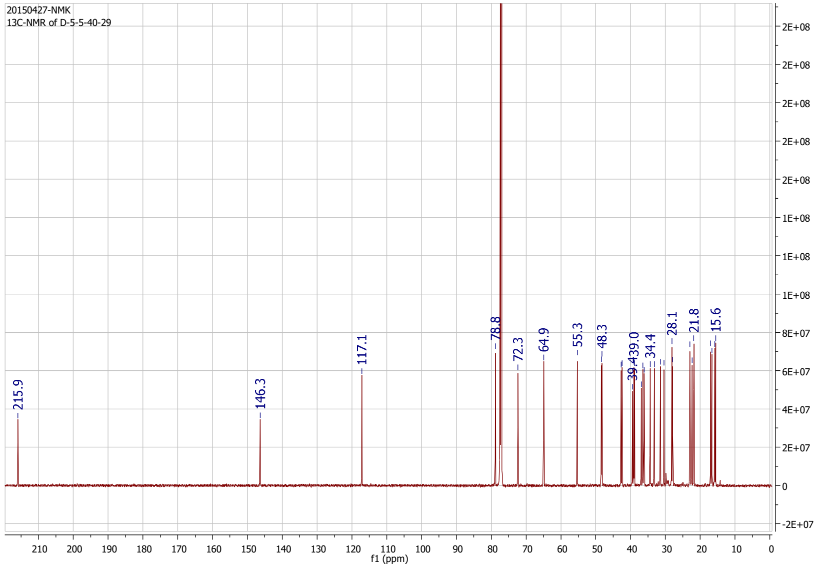

Supplementary Figure 17 (Continued)

C

Positive mode: m/z 479.3499 [M+Na]$^+$

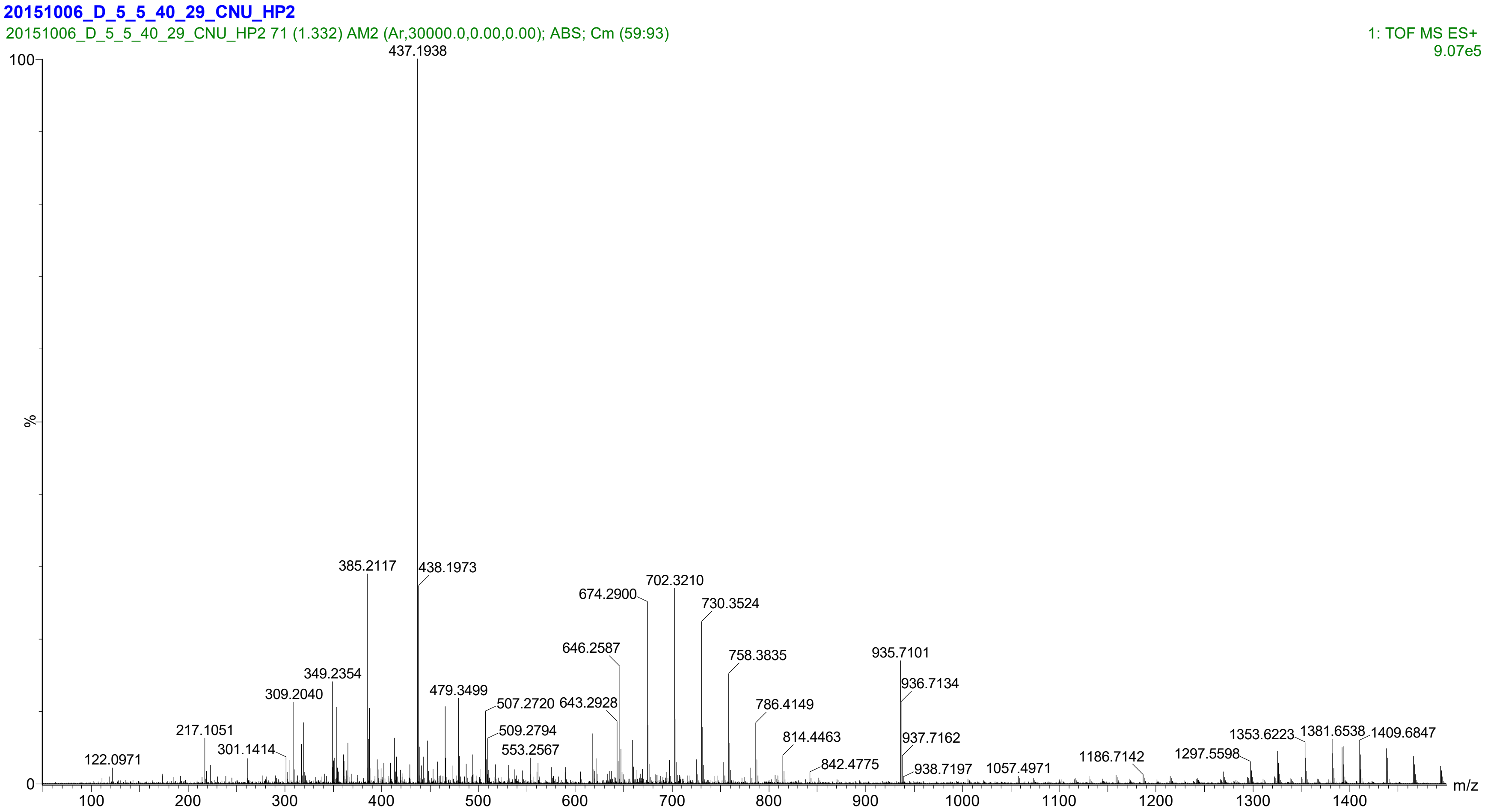

Supplementary Figure 18

A

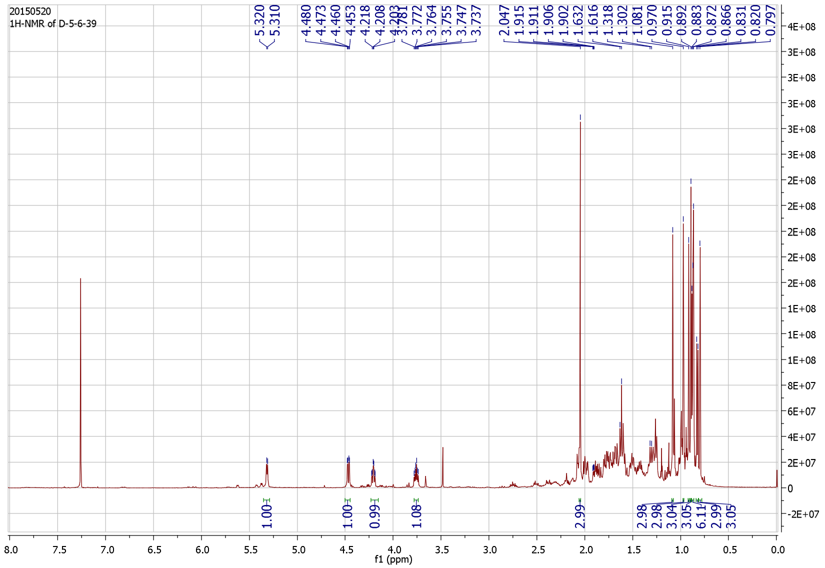

B

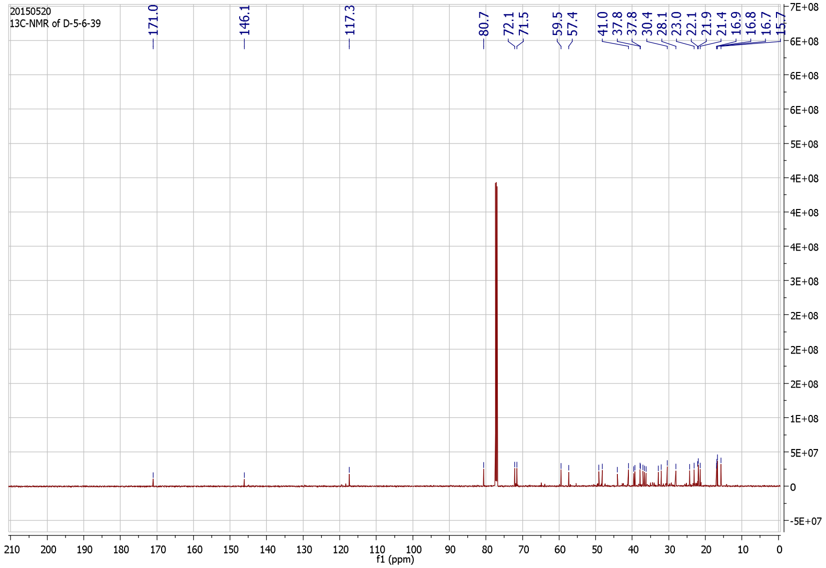

Supplementary Figure 18 (Continued)

C

Positive mode: m/z 523.3756 [M+Na]⁺

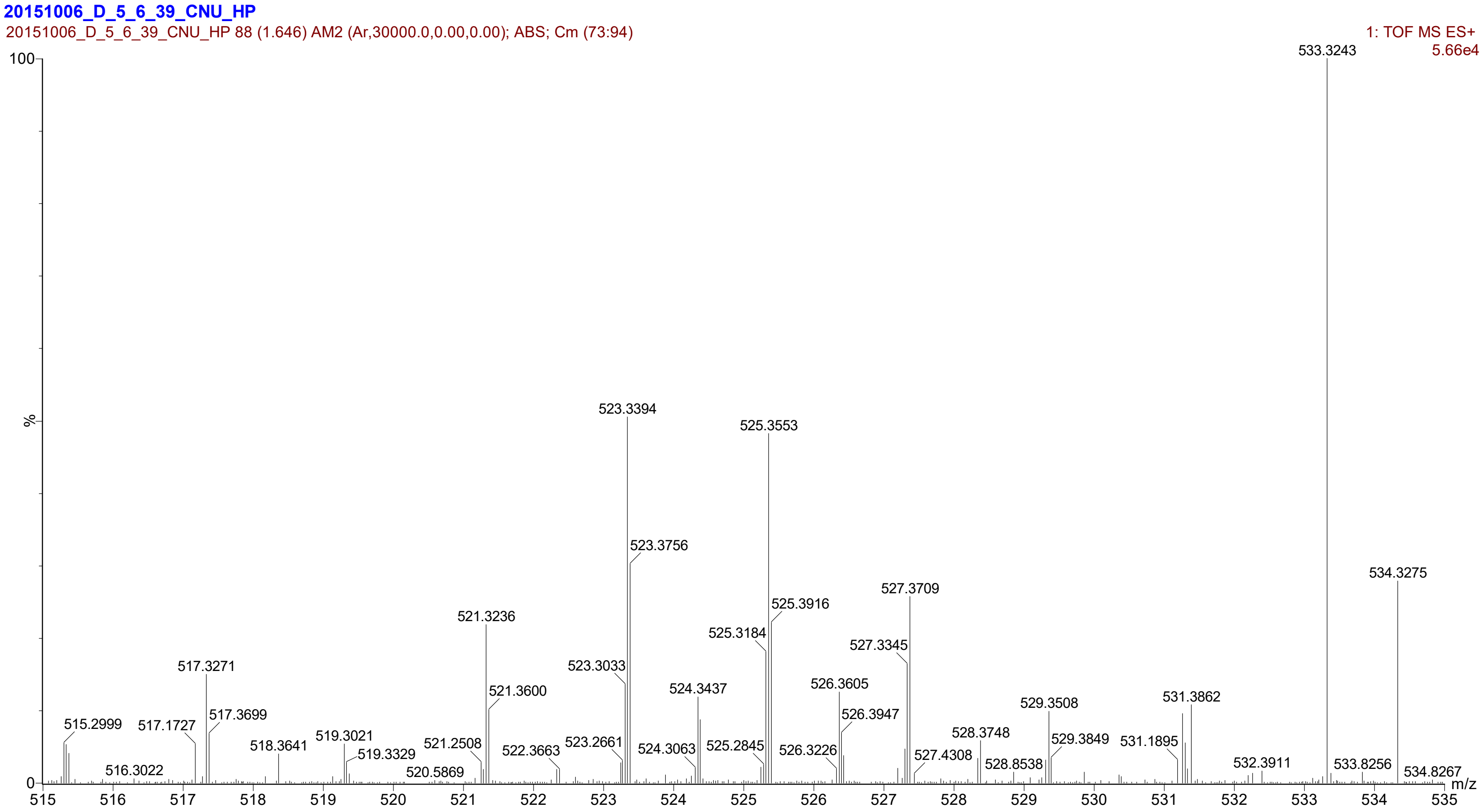

Supplementary Figure 19

A

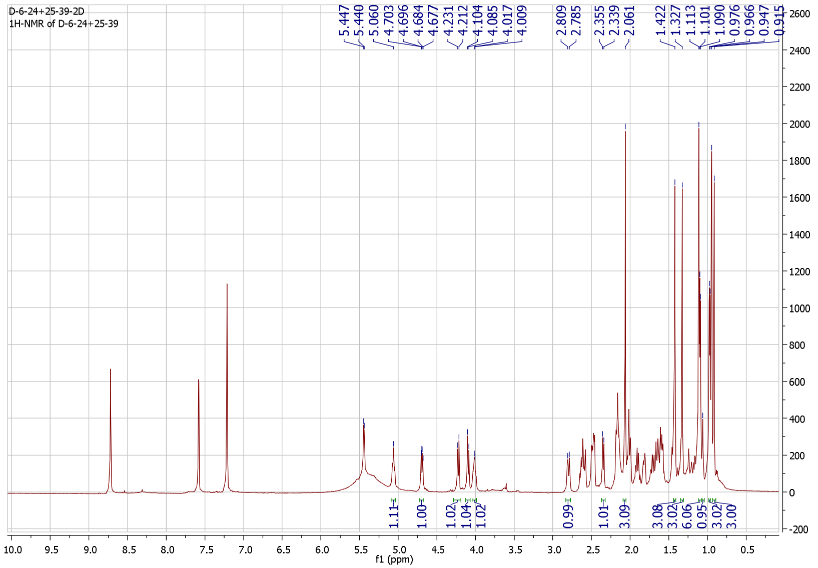

B

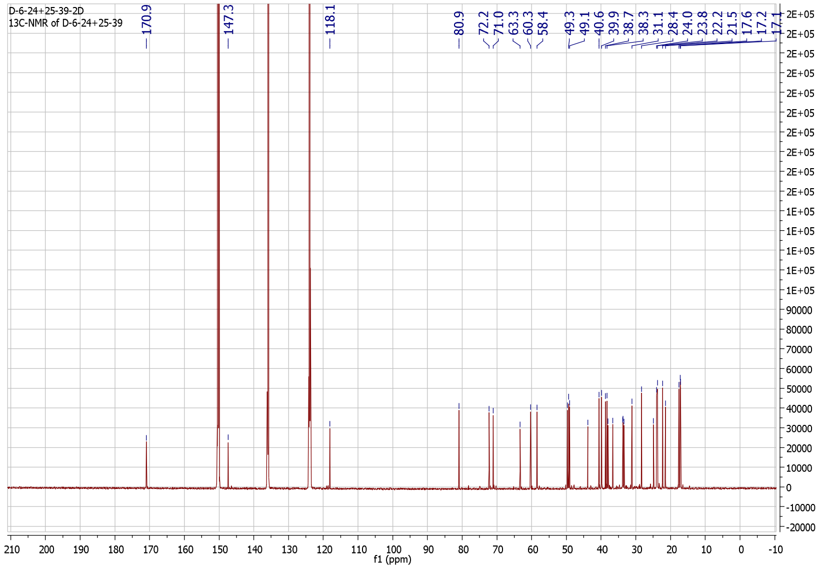

Supplementary Figure 19 (Continued)

C

Positive mode: m/z 539.3711 [M+Na]$^+$

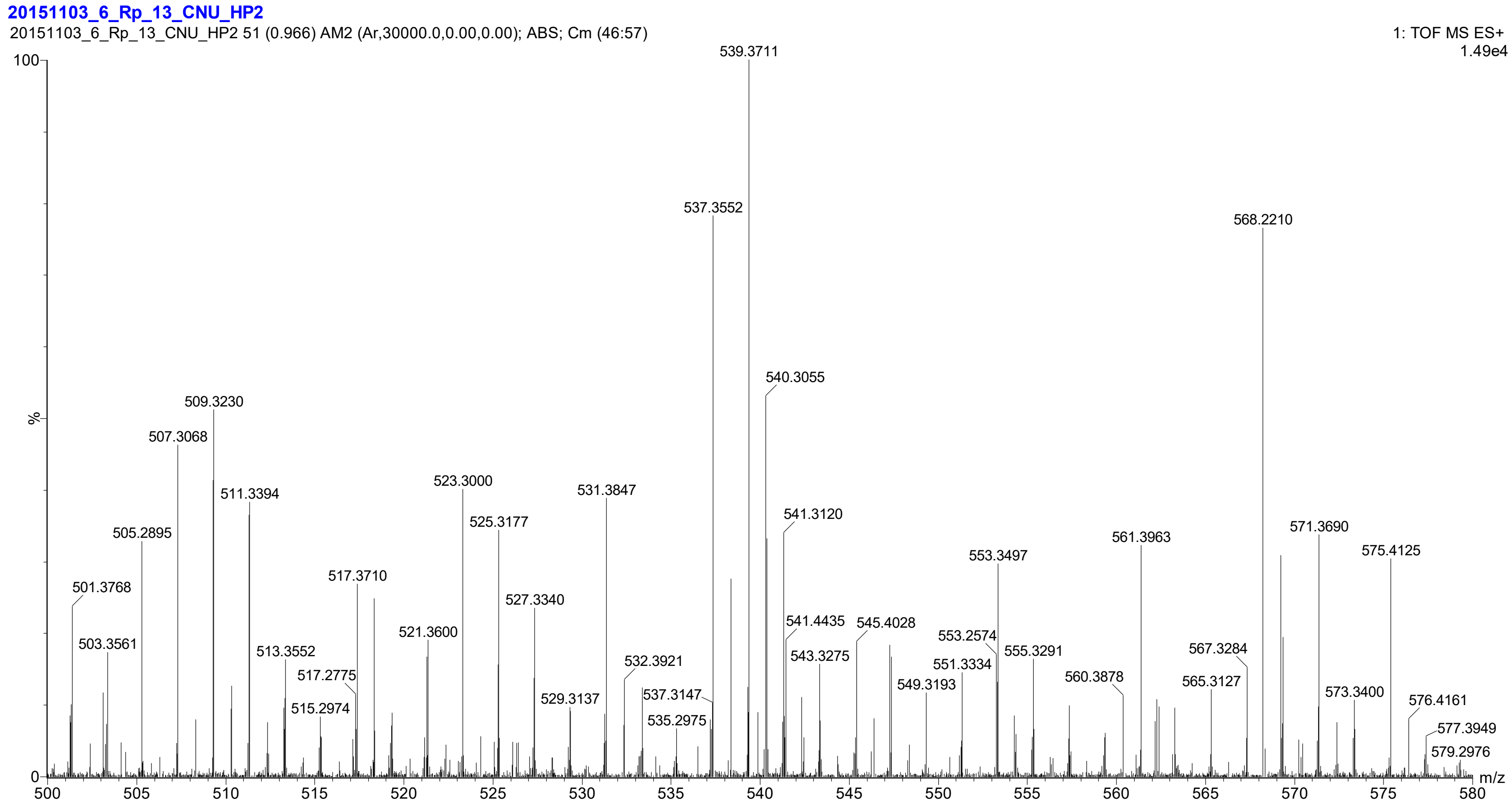

Supplementary Figure 20

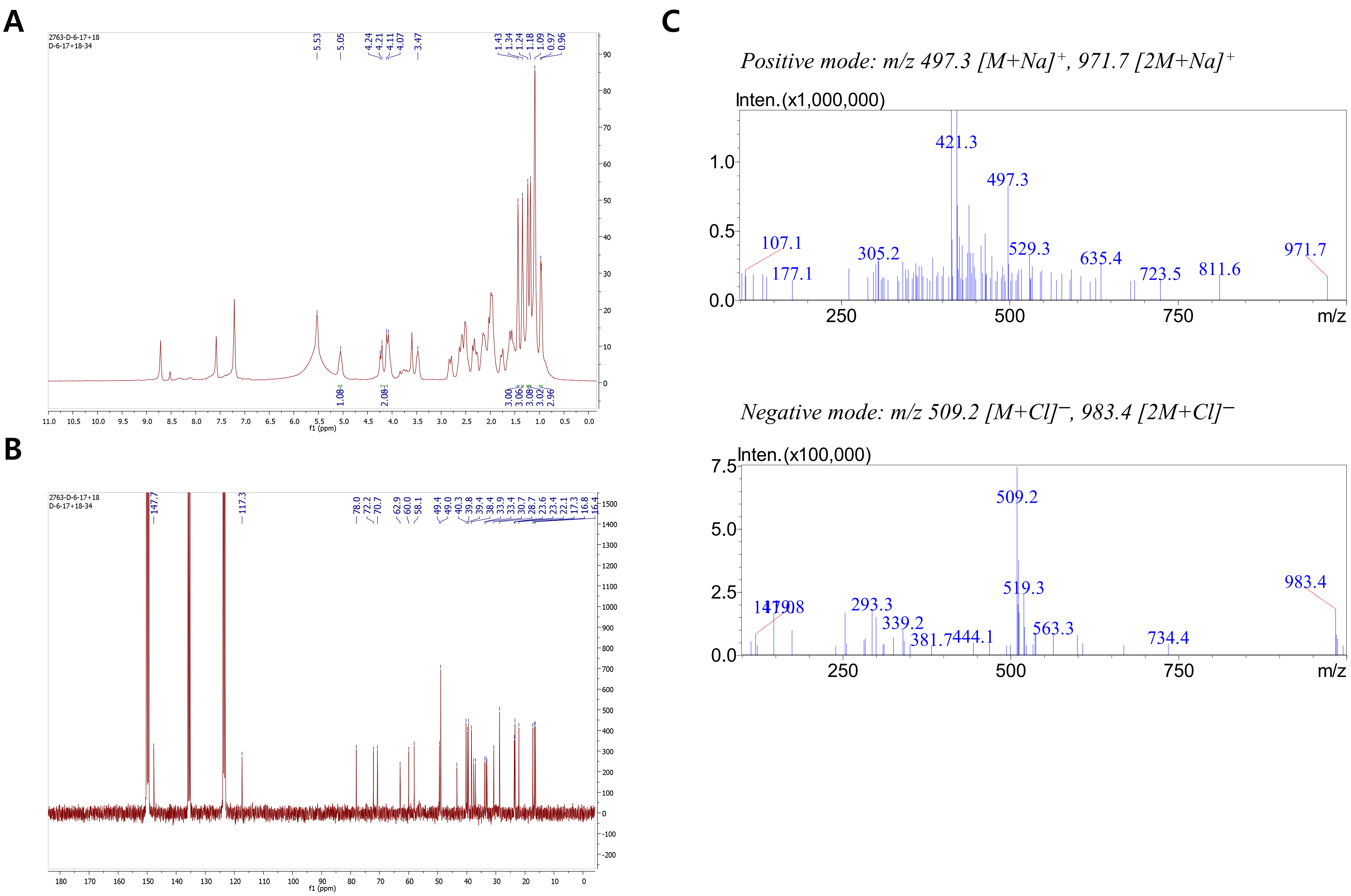

Supplementary Figure 21

A

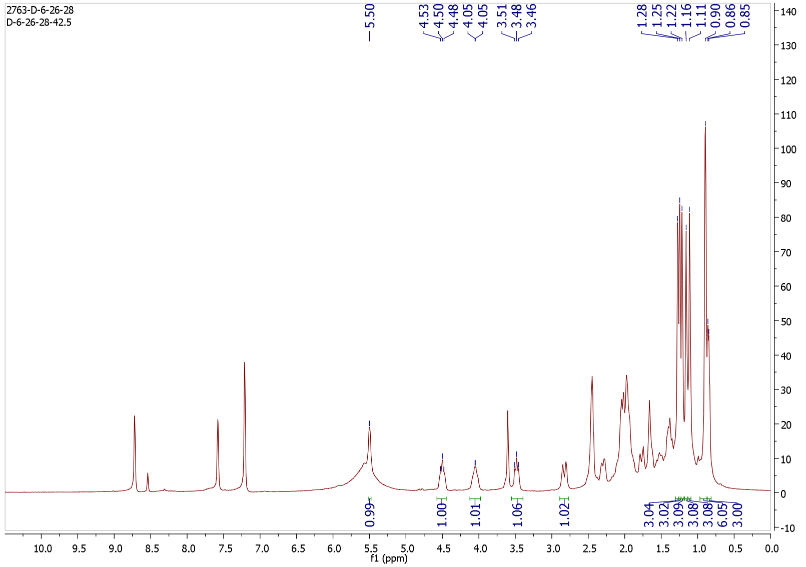

B

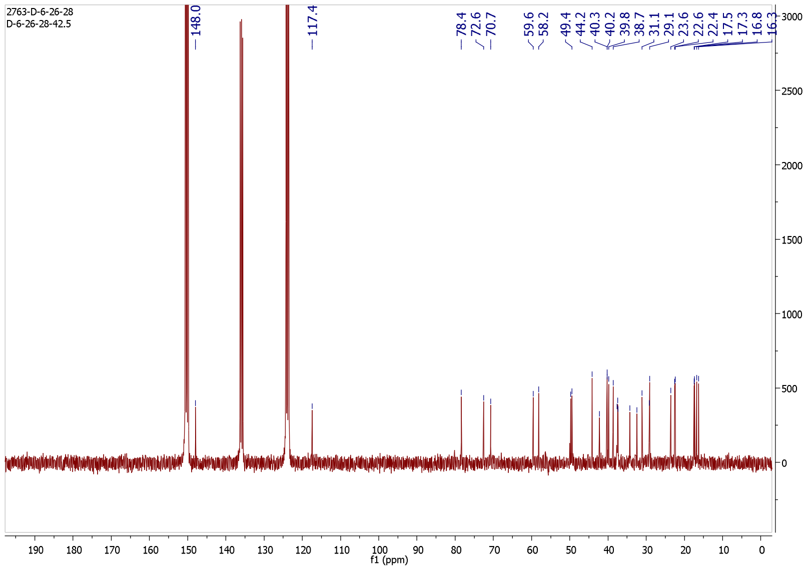

Supplementary Figure 22

A

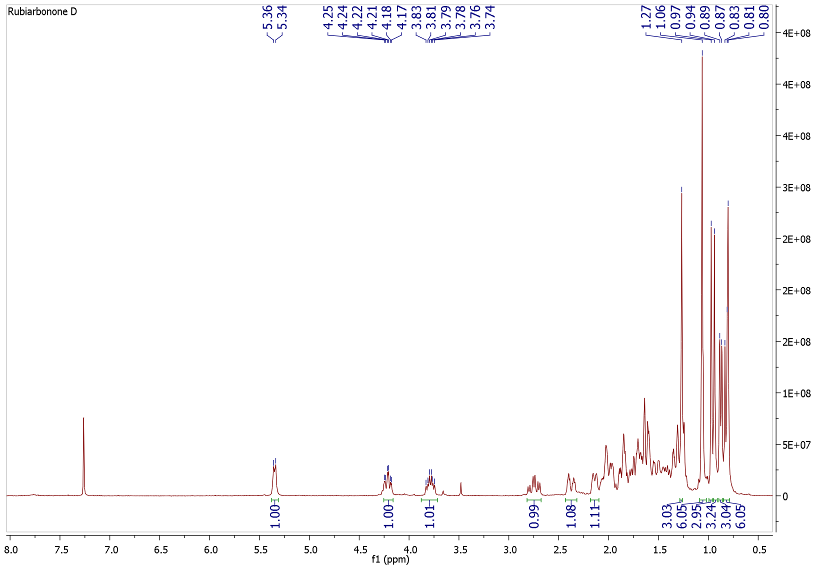

B

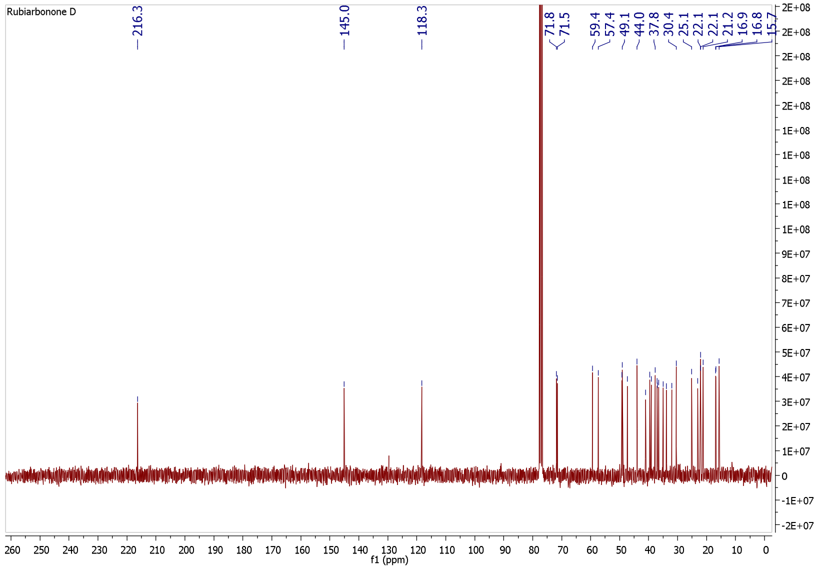

C

Positive mode: m/z 479.3 [M+Na]$^+$, 935.6 [2M+Na]$^+$

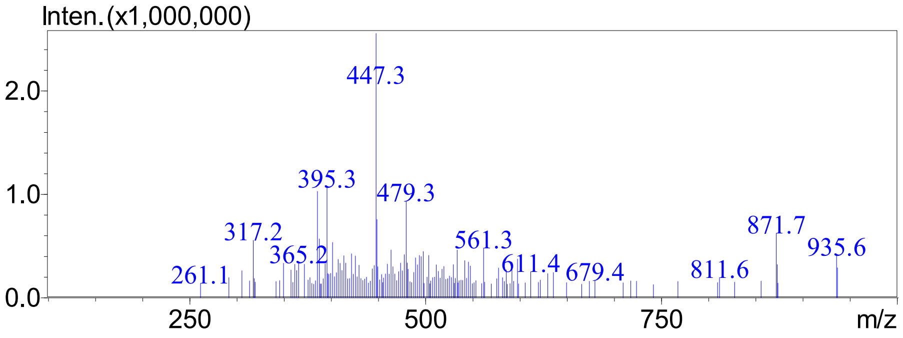

Negative mode: m/z 491.3 [M+Cl]$^-$

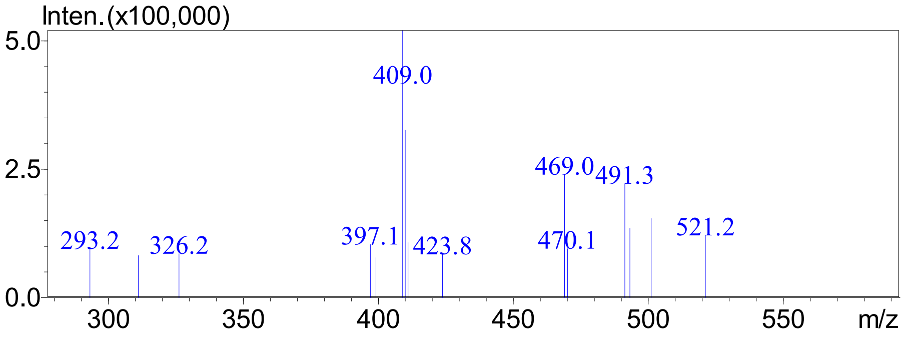

Supplementary Figure 23

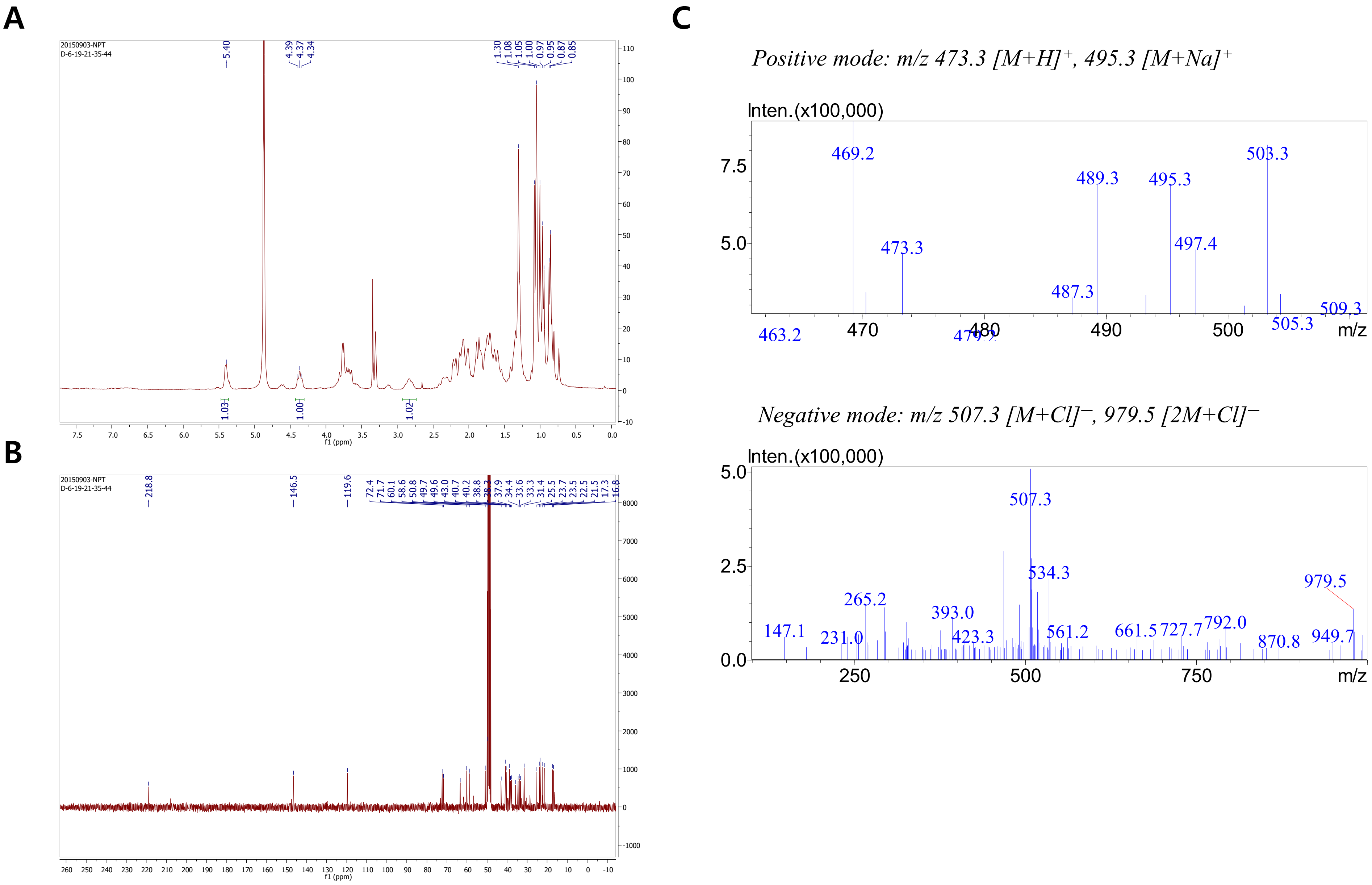

Supplementary Figure 24

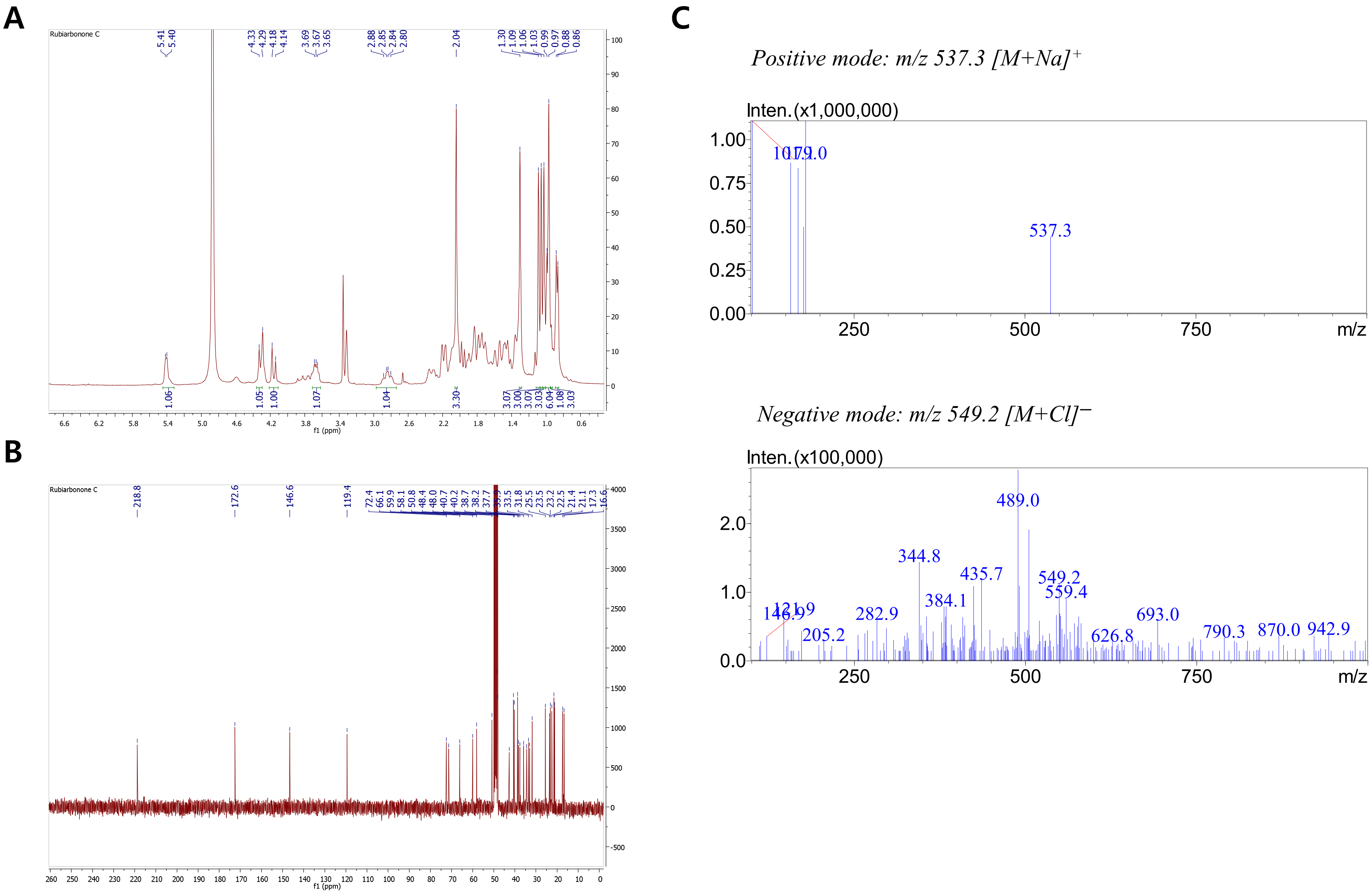

Supplementary Figure 25

A

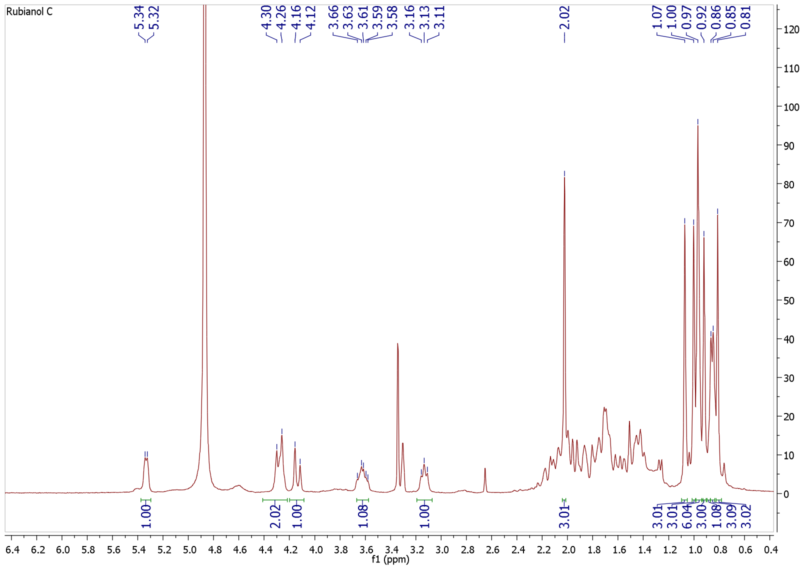

B

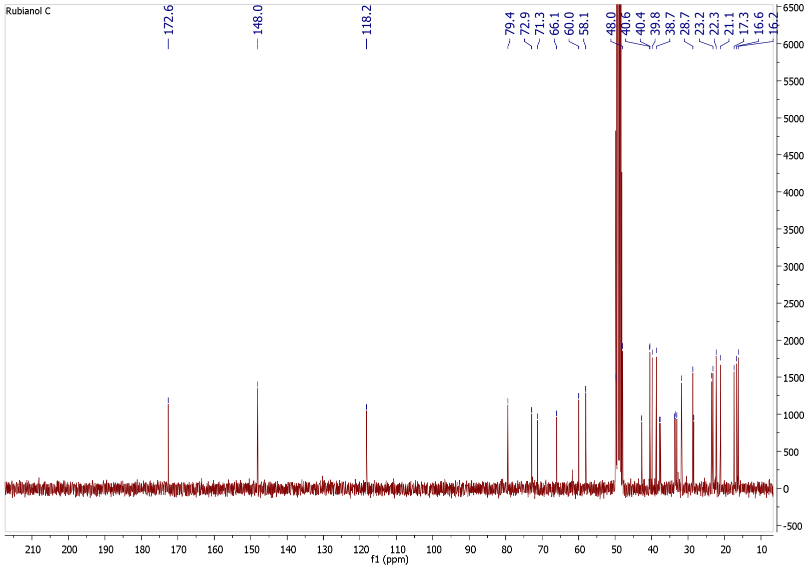

C

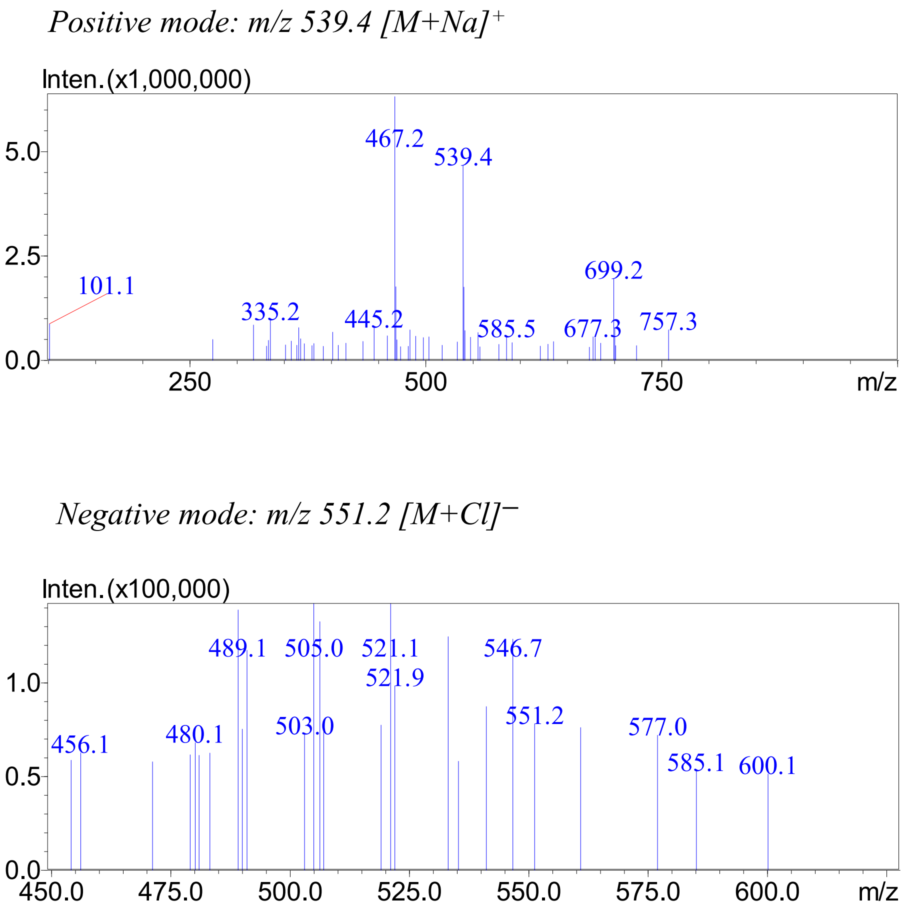

Supplementary Figure 26

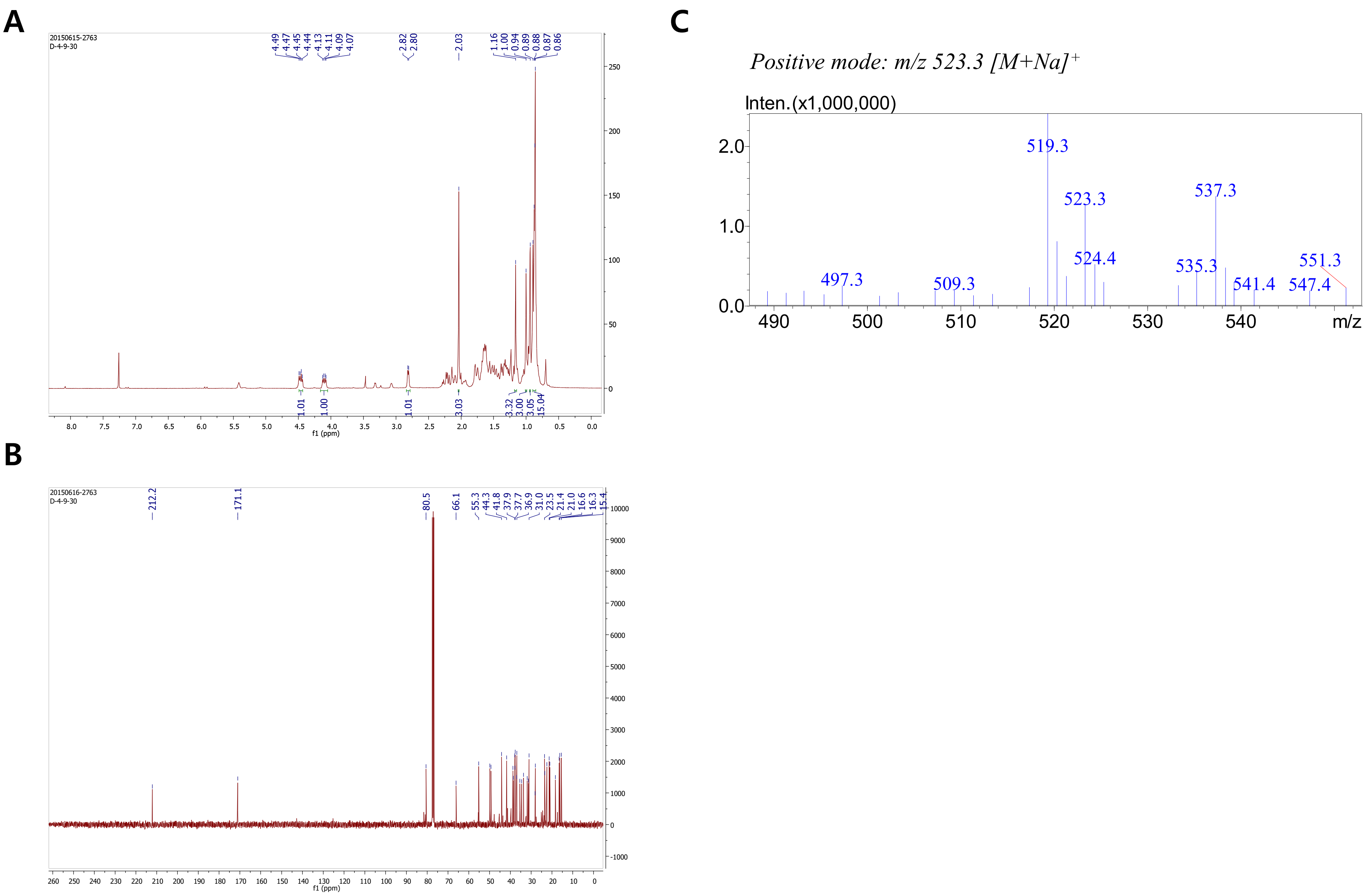

Supplementary Figure 27

A

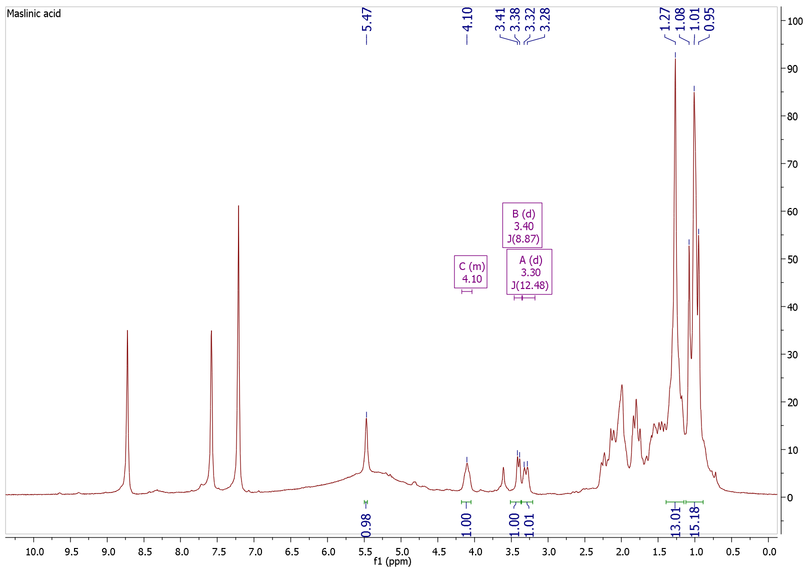

B

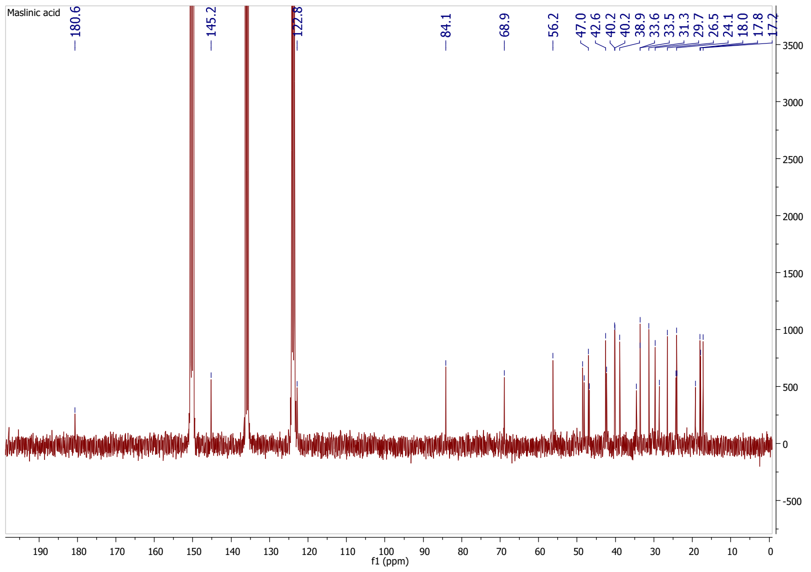

C

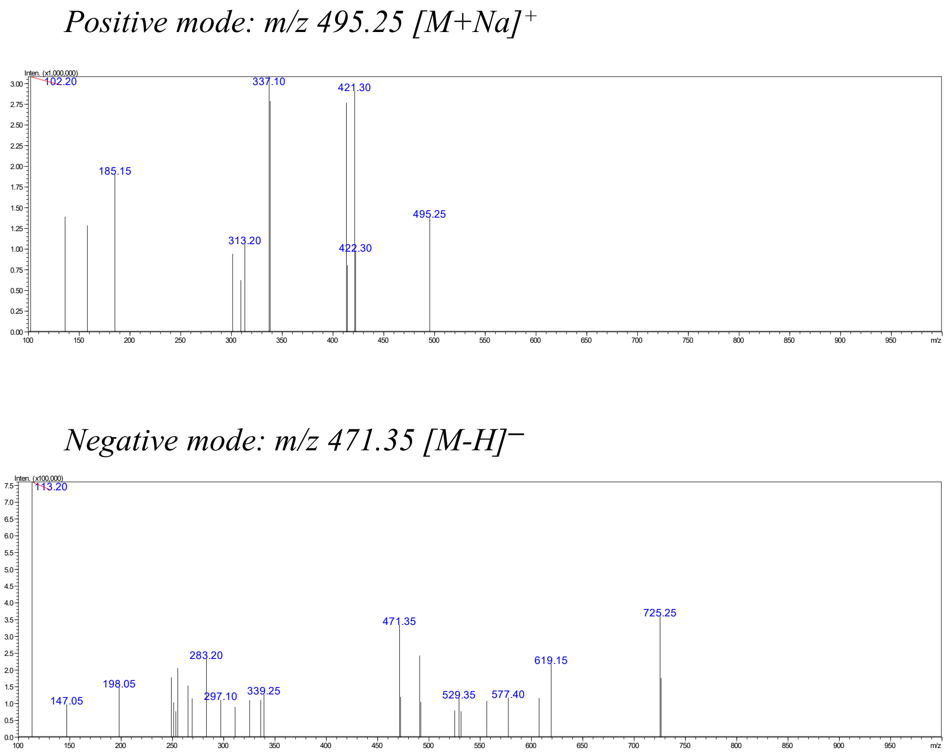

Supplementary Figure 28

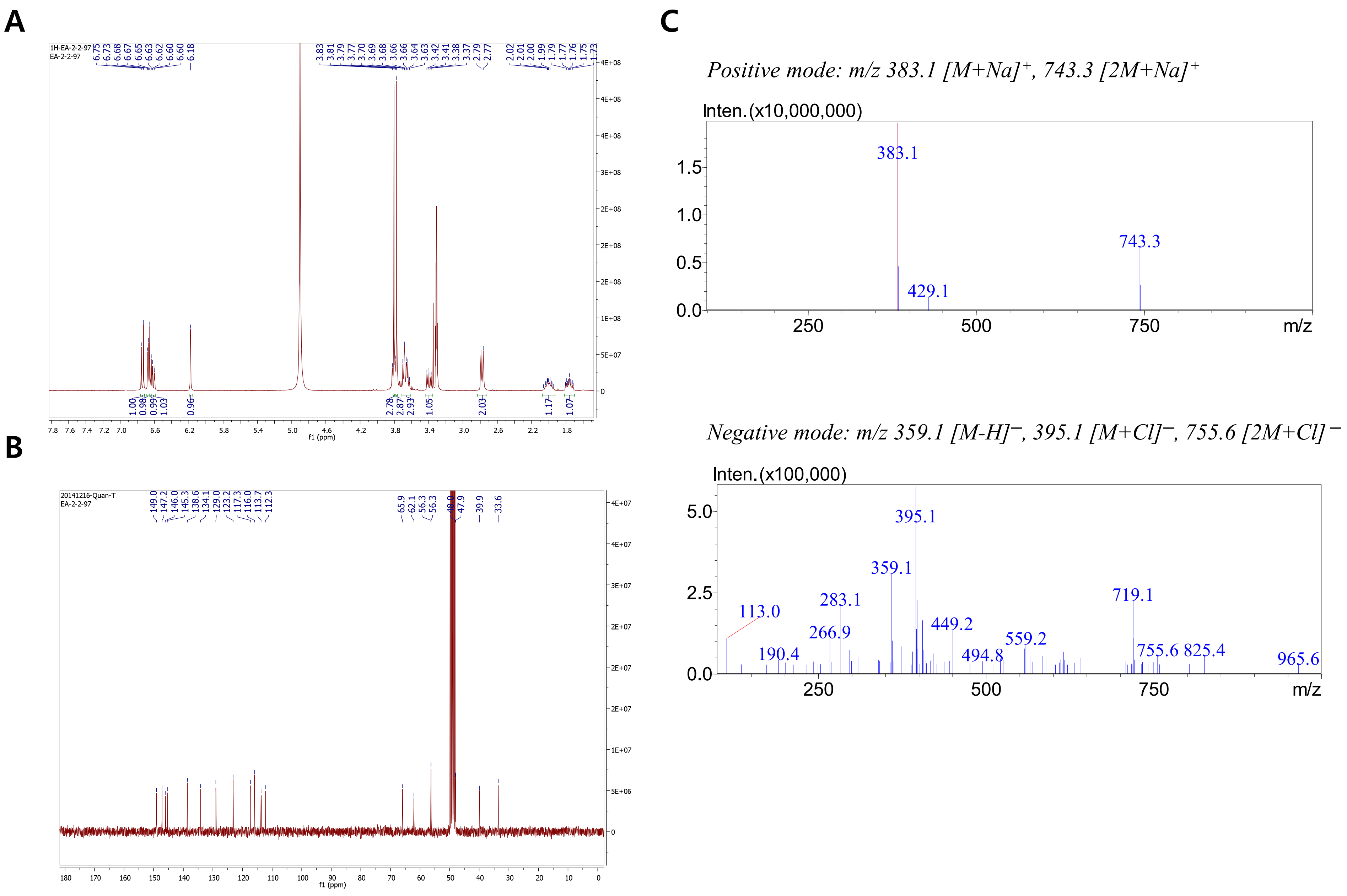

Supplementary Figure 29

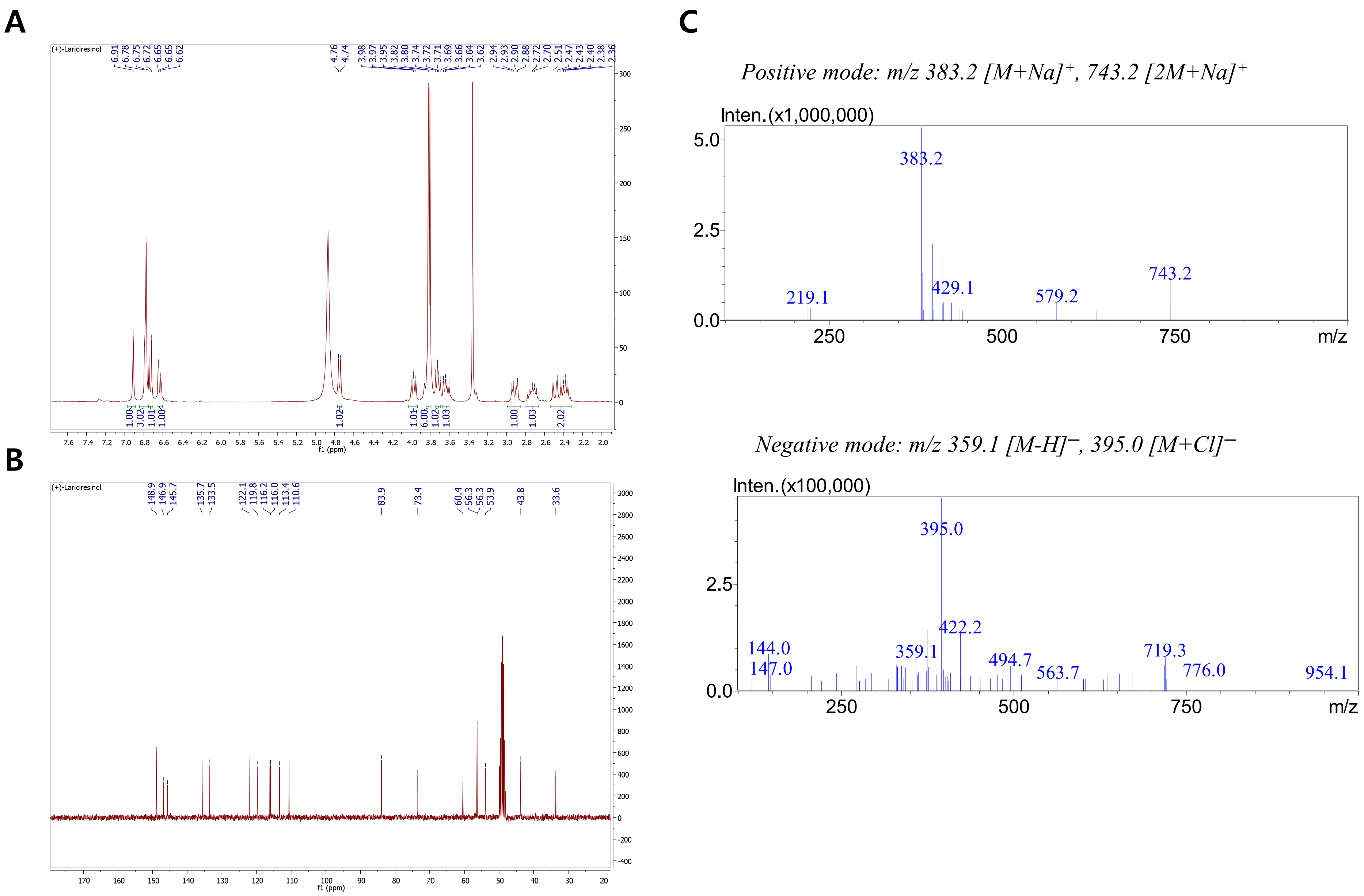

Supplementary Figure 30

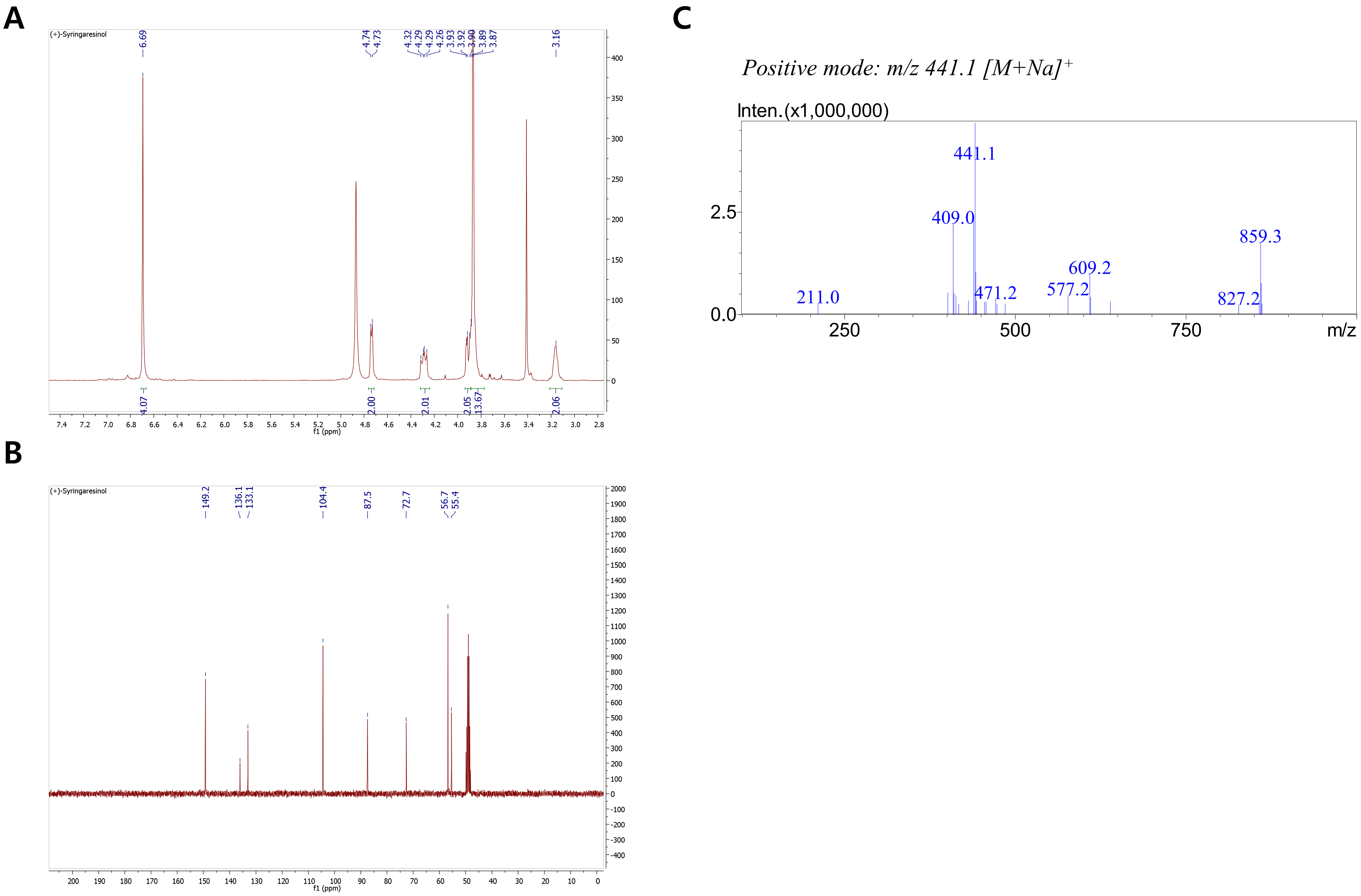

Supplementary Figure 31

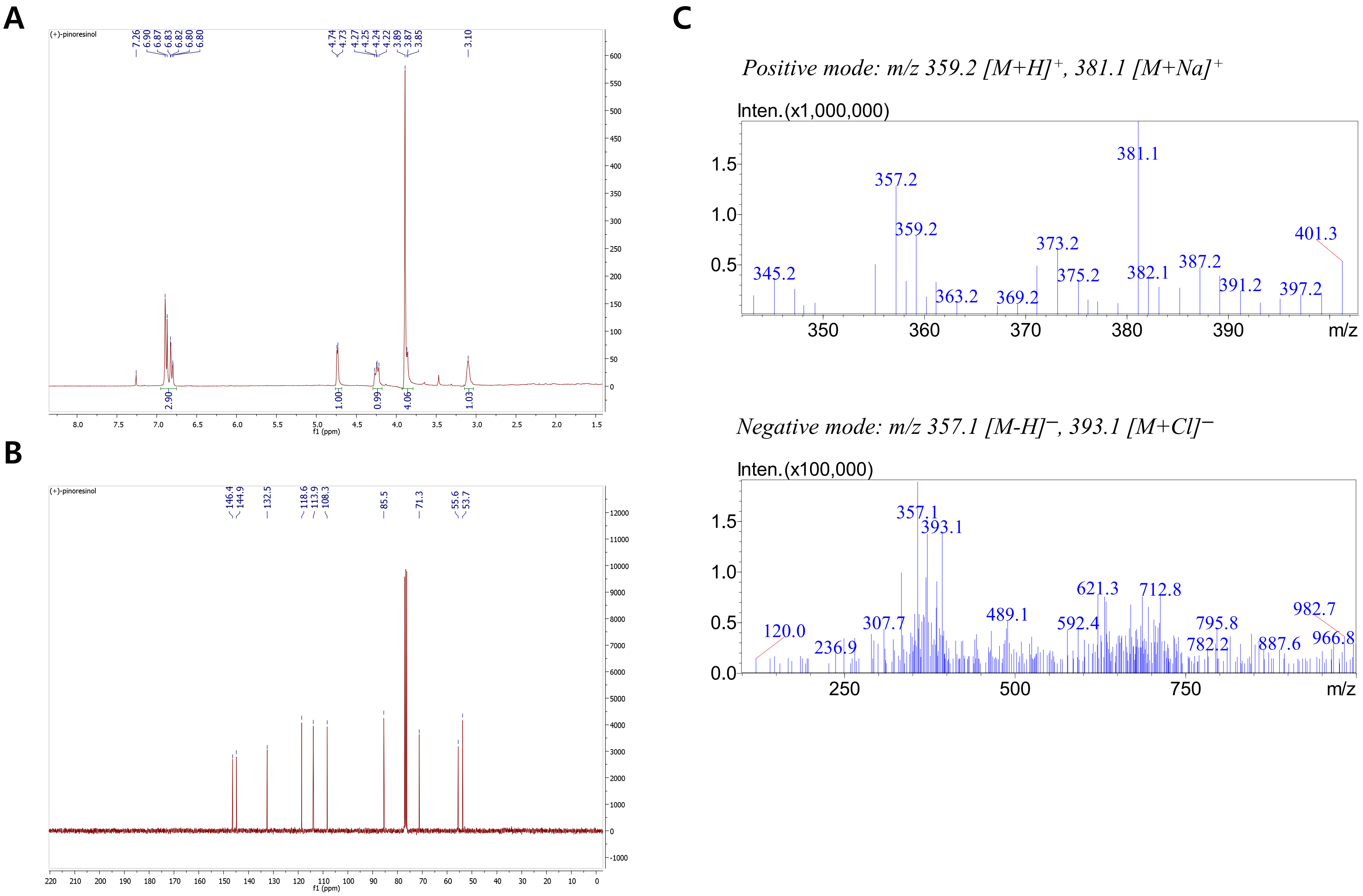

Supplementary Figure 32

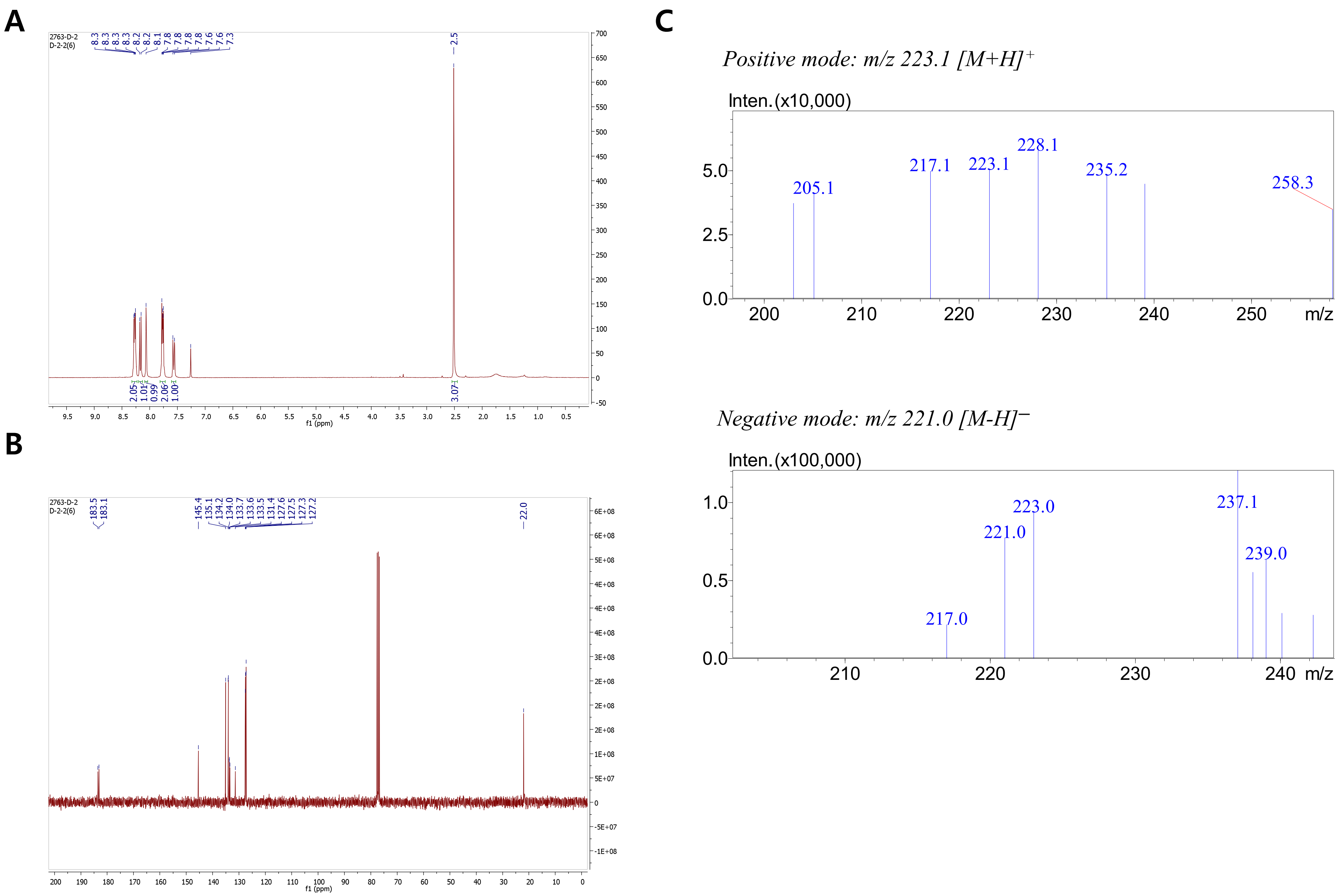

Supplementary Figure 33

A

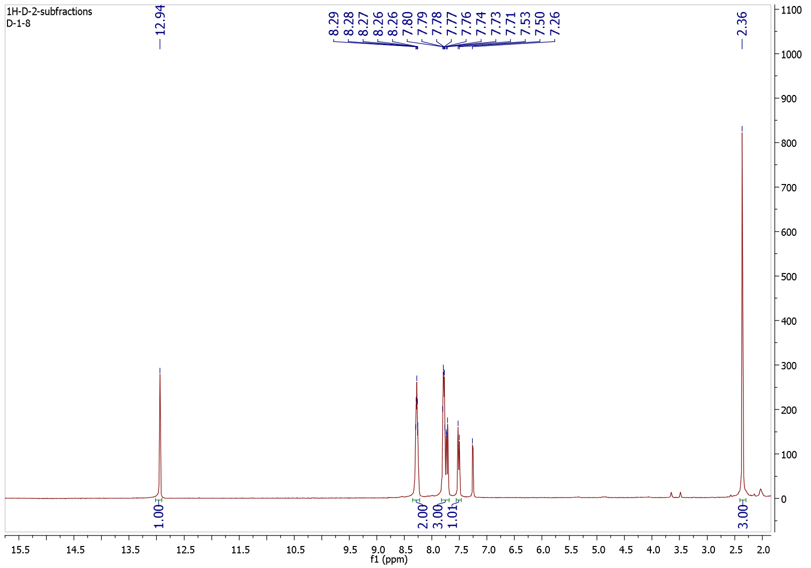

B

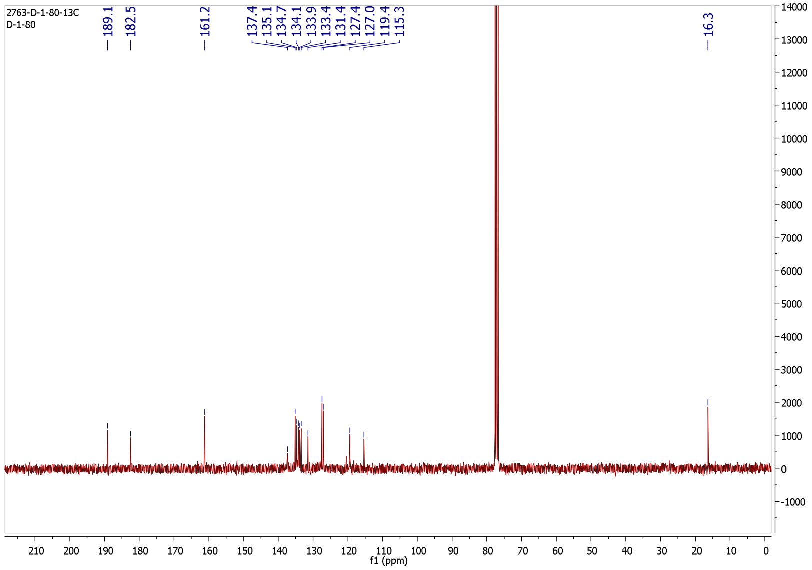

C

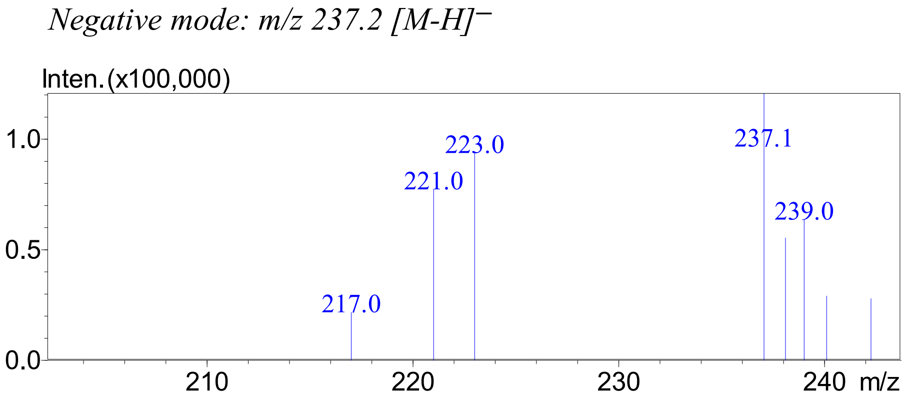

Supplementary Figure 34

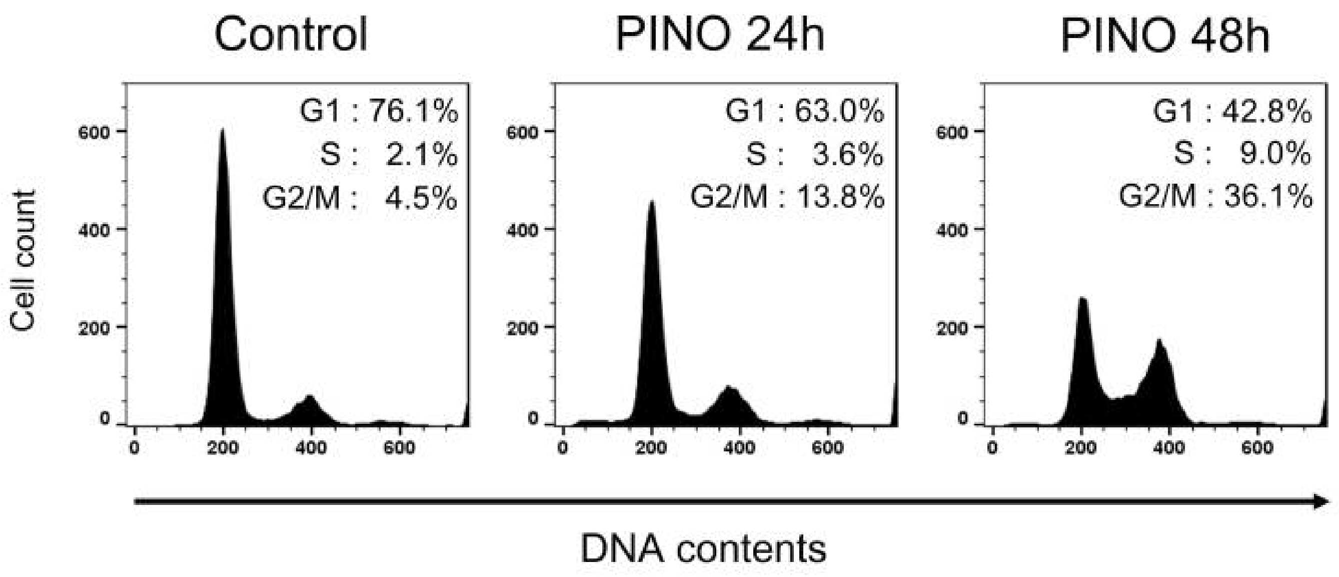

Supplementary Figure 35

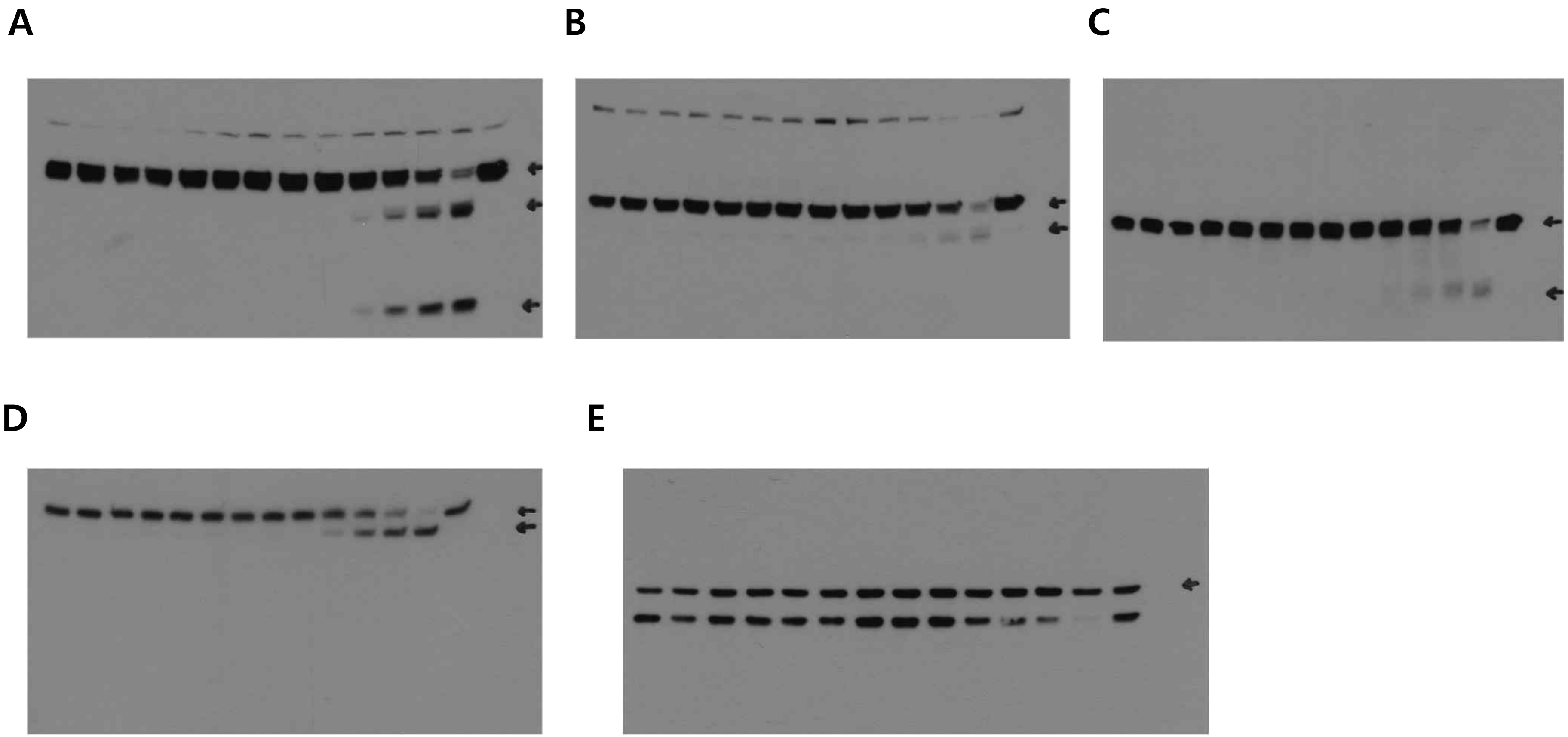

Supplementary Figure 36

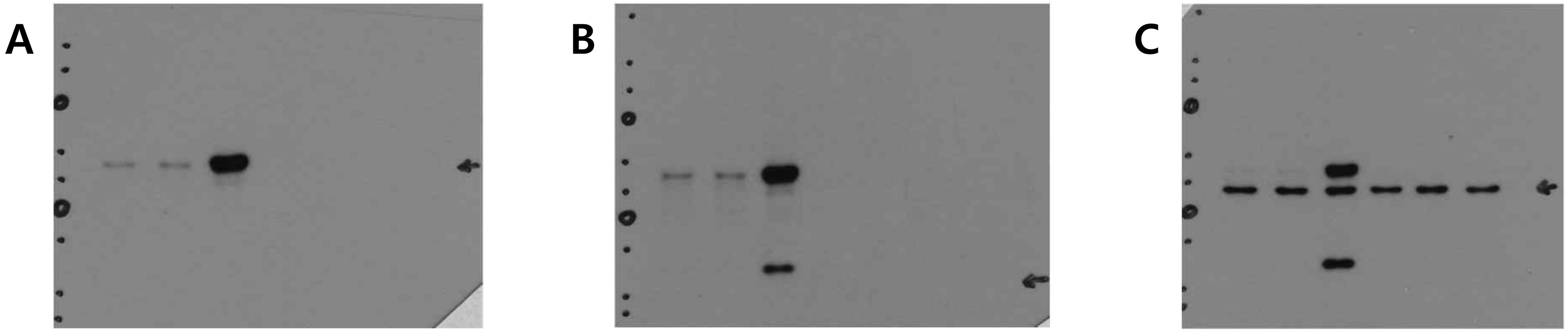

Supplementary Figure 37

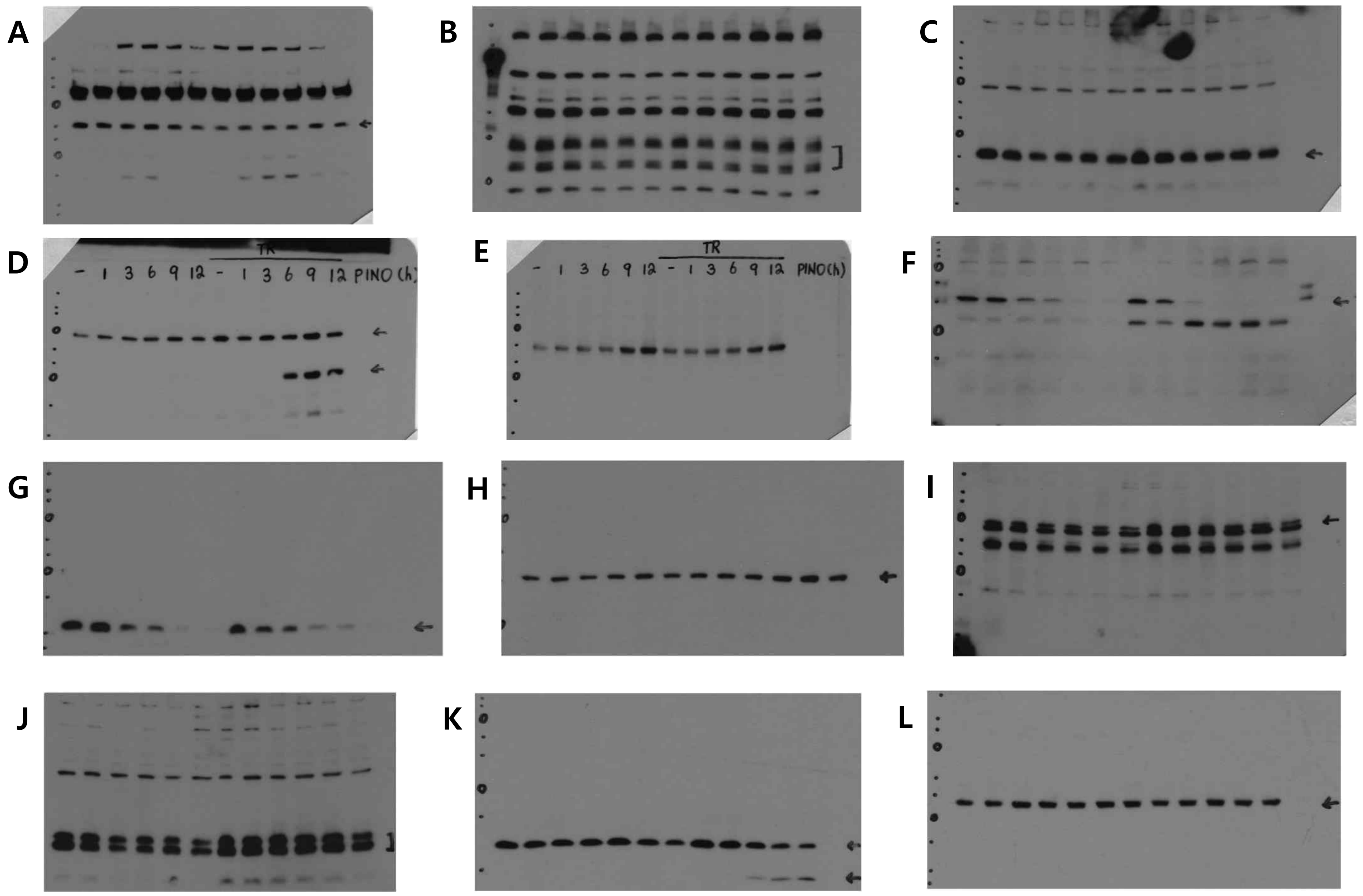

Supplementary Figure 38

A

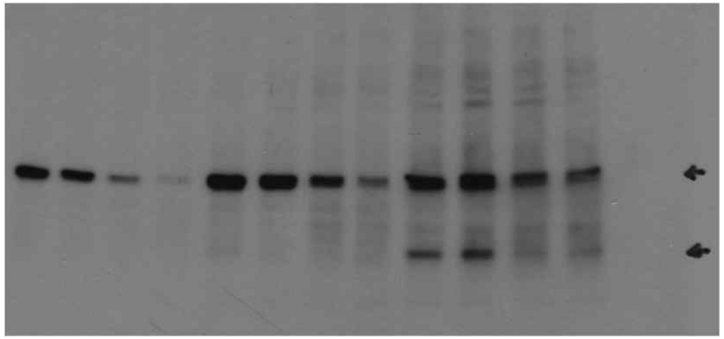

B

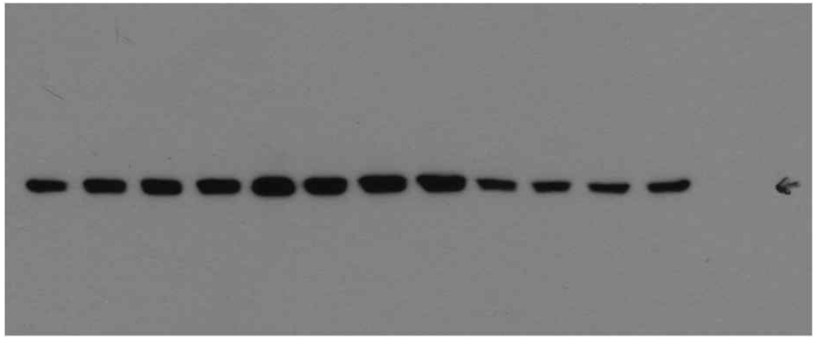

Supplementary Figure 39

A

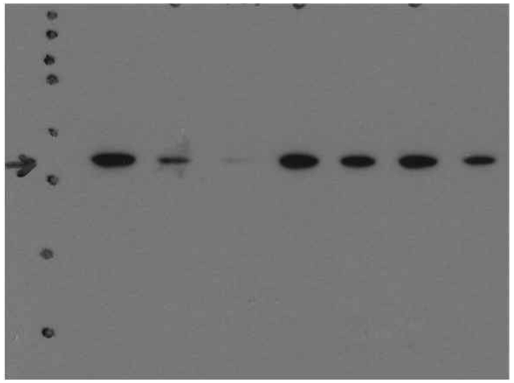

B

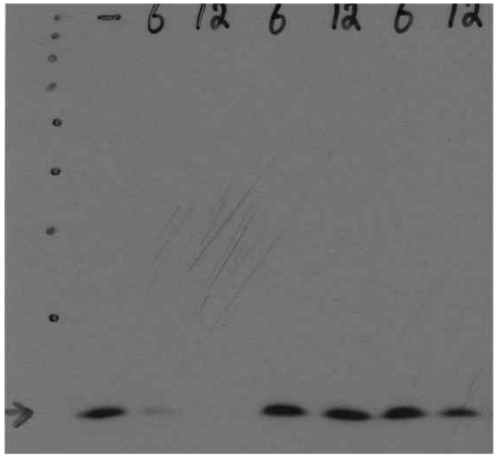

C

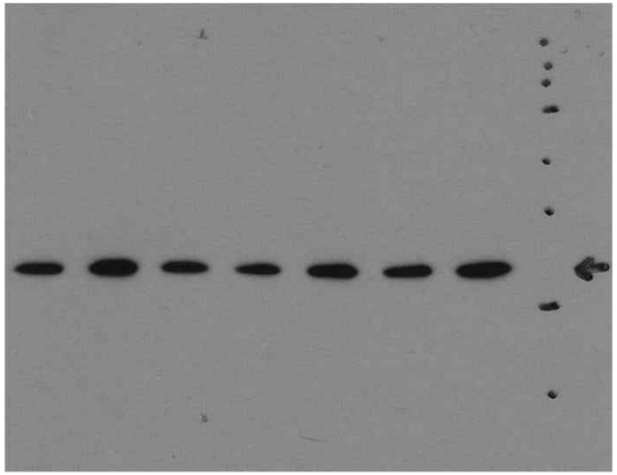

Supplementary Figure 40

A

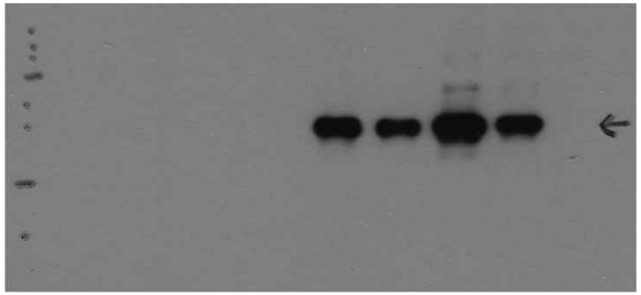

B

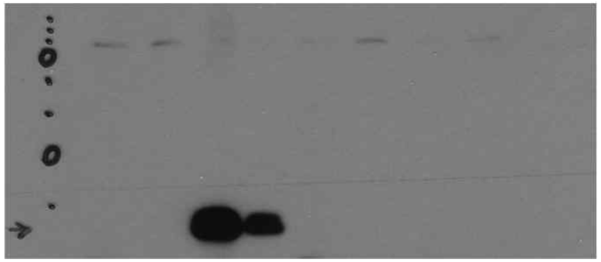

C.

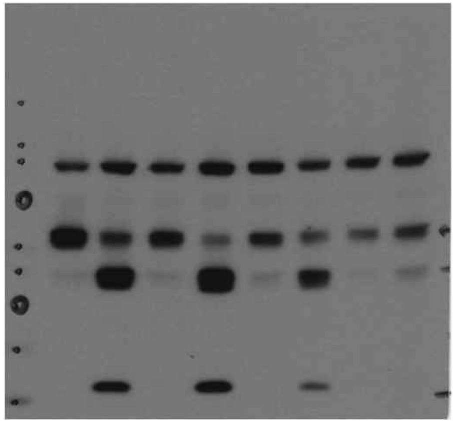

D

Short exposure

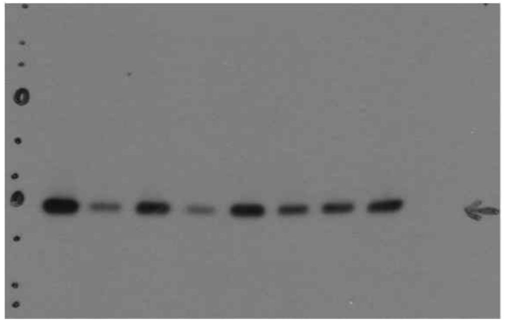

Long exposure

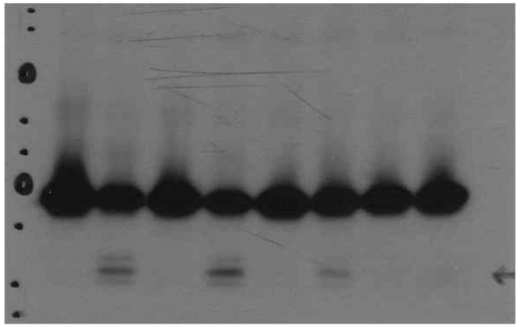

E

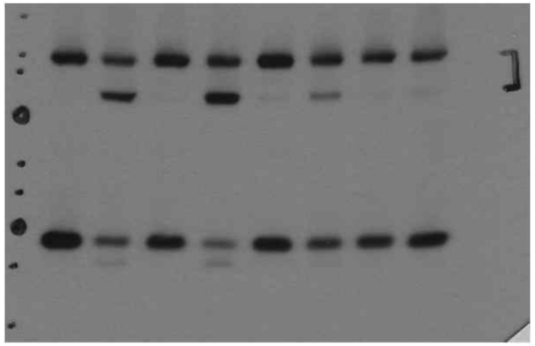

F

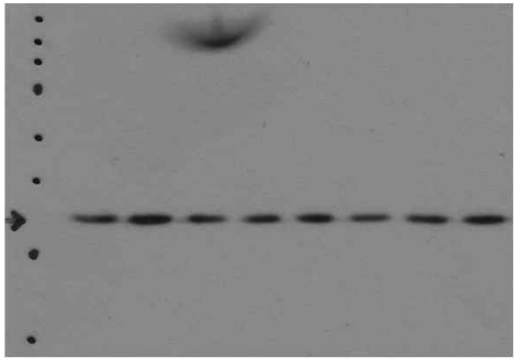

Supplementary Figure 41

A

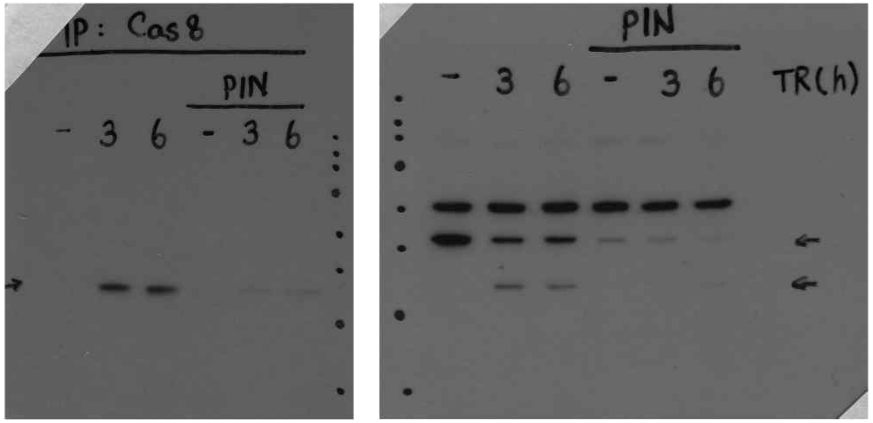

B

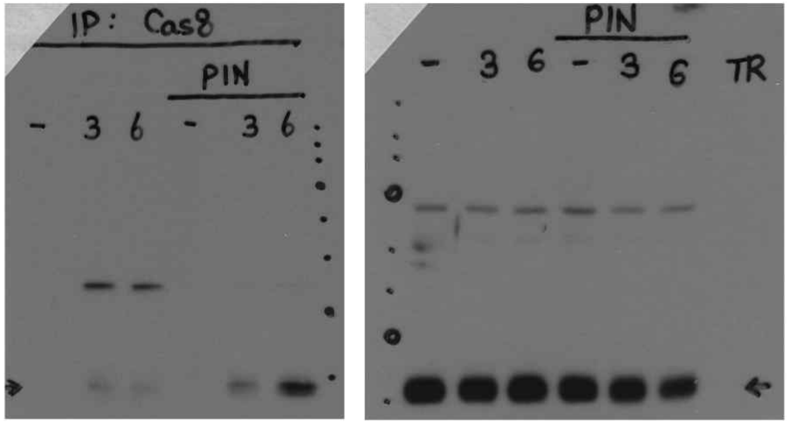

C

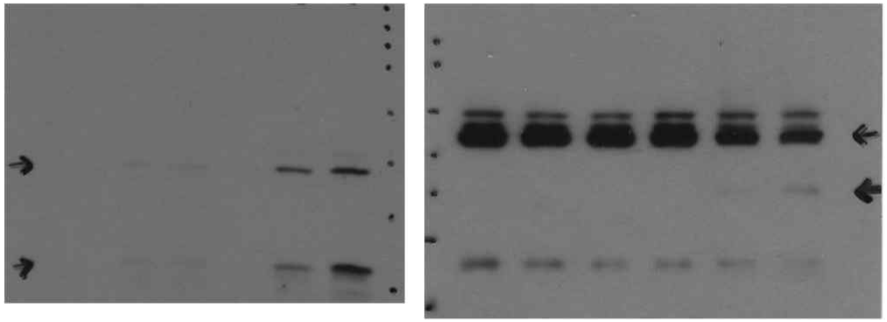

D

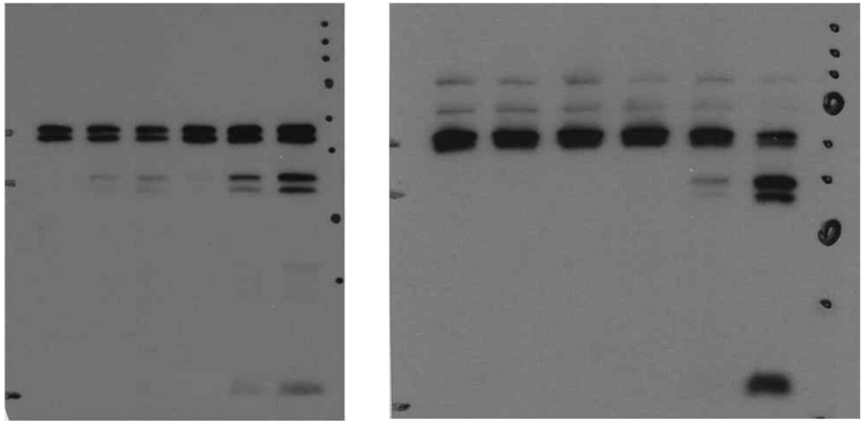

E

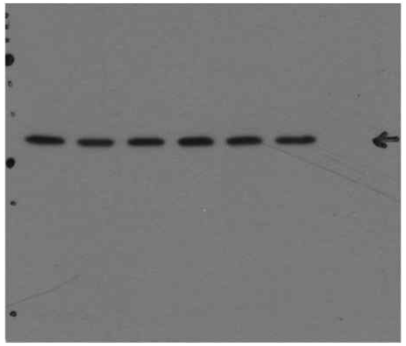

Supplementary Figure 42

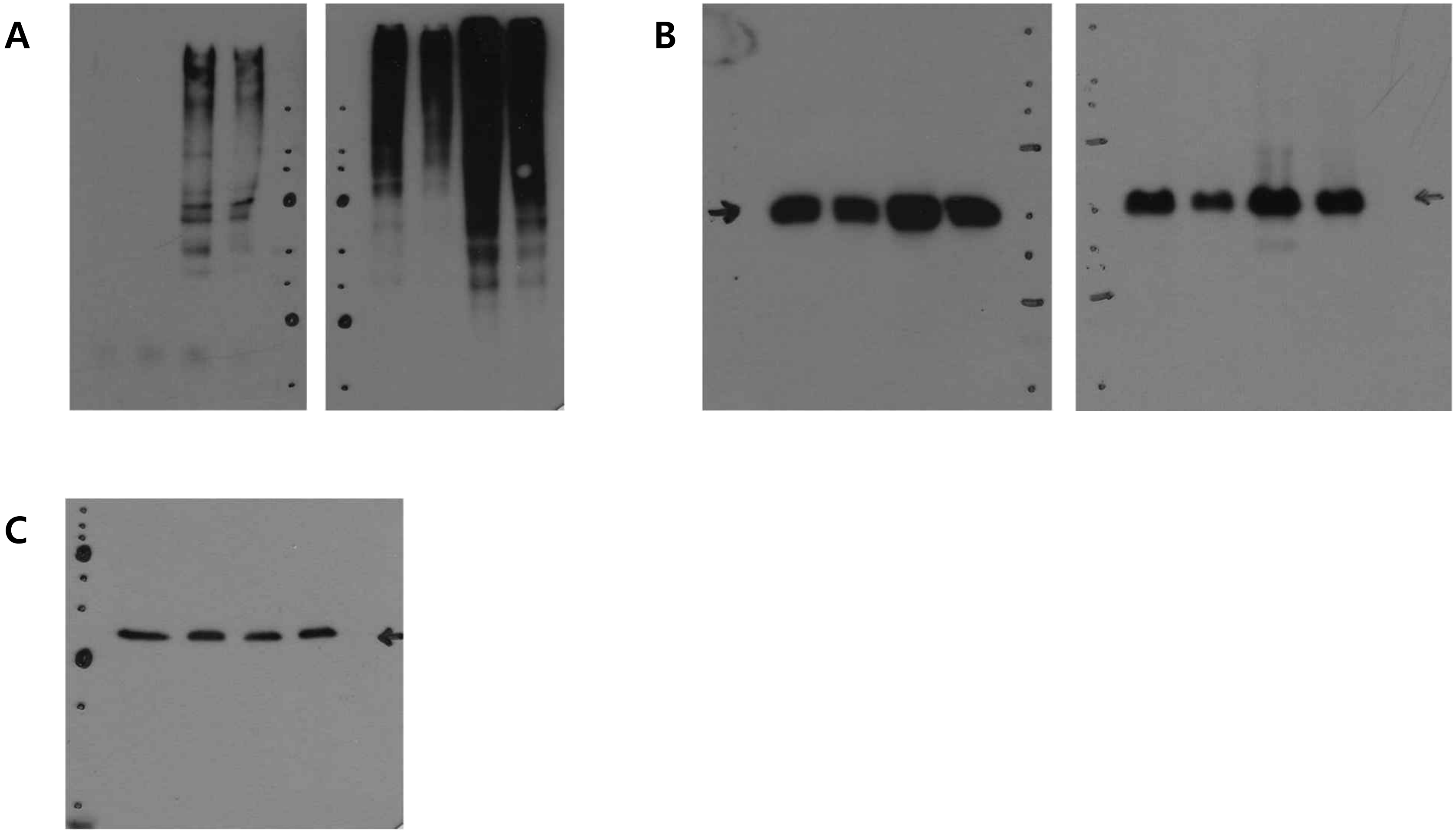

Supplementary Figure 43

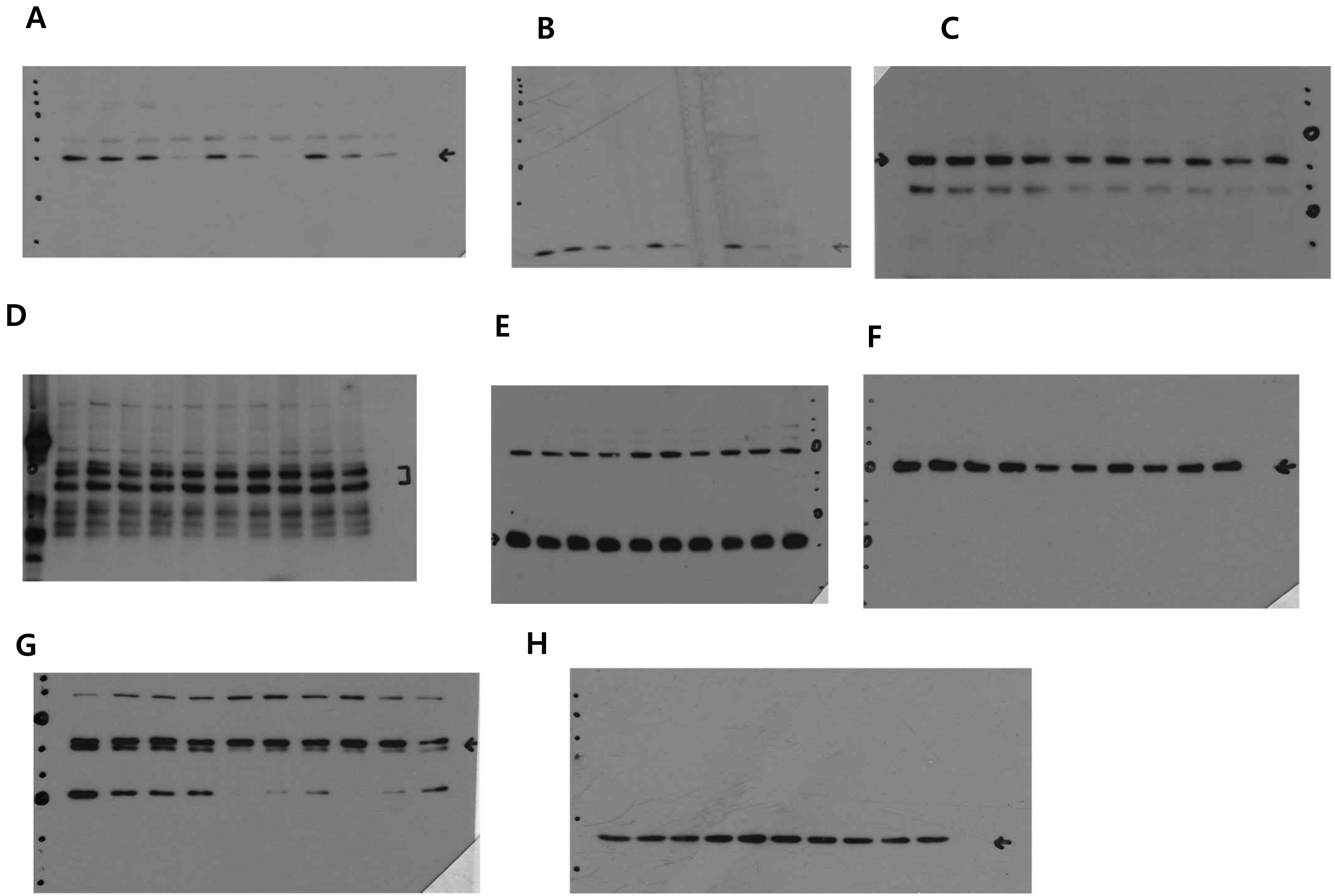

Supplementary Figure 44

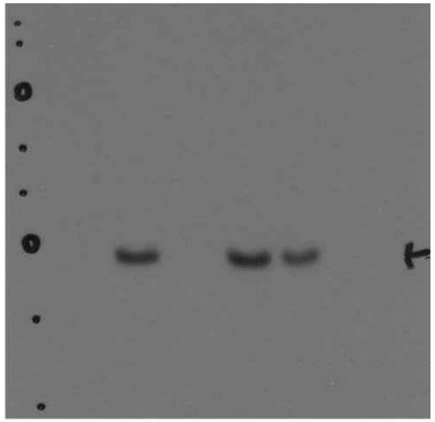

Supplementary Figure 45

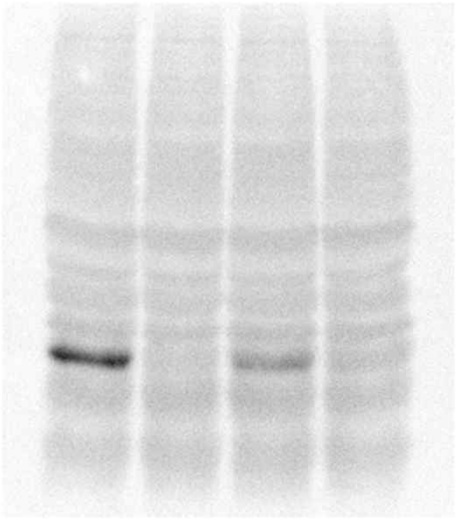

Supplement: Supplementary file 1 — Supplementary Informarion [file 41598_2019_49909_MOESM1_ESM.pdf]
